# Structural basis for protein-free catalysis by ribonuclease P ribozyme

**DOI:** 10.1038/s41467-026-71597-4

**Published:** 2026-04-15

**Authors:** Yun-Tzai Lee, Maximilia F. S. Degenhardt, Ilias Skeparnias, Szu-Yun Chen, Bapurao A. Bhoge, Sergey G. Tarasov, Marzena A. Dyba, Jinwei Zhang, Jason R. Stagno, Yun-Xing Wang

**Affiliations:** 1 Protein-Nucleic Acid Interaction Section, Center for Structural Biology, National Cancer Institute, Frederick, MD USA; 2 Laboratory of Molecular Biology, National Institute of Diabetes and Digestive and Kidney Diseases, Bethesda, MD USA; 3 Biophysics Resource, Center for Structural Biology, National Cancer Institute, Frederick, MD USA

**Keywords:** Cryoelectron microscopy, Biocatalysis, Catalytic RNA

## Abstract

Ribonuclease P (RNase P) is an essential metallonuclease found in all three domains of life. However, the structural basis for the ancient RNase P RNA component acting alone as a ribozyme and catalytic metal-ion chemistry remains unknown. We report a series of cryo-EM structures, at resolutions of 2.8–3.5 Å, of the *Geobacillus stearothermophilus* RNase P aporibozyme (apoE) in various states of the catalytic cycle. The formation of both the tetraloop/tetraloop-receptor interaction and the interdigitated double T-loop motif in the substrate-specificity domain facilitates substrate binding. The apoE uses two metal ions for catalysis, suggesting a catalytic mechanism and evolutionary importance of the RNase P ribozyme to function without its protein component. Together, our data portray the regulatory RNA-RNA interfaces, dynamic structures, and cation traffic that confer function to a *trans-acting* ribozyme.

## Abstract (web summary)

Ribonuclease P (RNase P) is an essential metallonuclease found in all three domains of life. Here, the authors present cryoEM structures of *Geobacillus stearothermophilus* RNase P aporibozyme (apoE) in various states of the catalytic cycle.

## Introduction

Ribonuclease P (RNase P) is an essential endoribonuclease for the maturation of transfer RNAs (tRNAs) across all three domains of life. In bacteria and some archaea, the RNA component of RNase P (RPR) exhibits intrinsic catalytic activity in vitro, even without its protein subunit^1,2^, although the mechanism behind this protein-free ribozyme activity and its evolutionary significance are unclear. Archaea RPRs from methanobacteria, *Thermococci*, and extreme halophiles can process precursor tRNA (pre-tRNA) to produce mature tRNA (mat-tRNA) in the absence of their protein components, but this has only been demonstrated under very high ionic strength conditions in vitro^2^. In contrast, the eukaryotic RPRs require multiple protein subunits for catalytic activity both in vitro and in vivo^3–5^. Regardless, the RNase P protein components are considered essential for RNase P to exert its full-fledged function in all organisms.

Despite recent progress, no study has investigated the catalytic cycle longitudinally by probing the structures of RNase P in different reaction states at a resolution sufficient to elucidate its enzymatic mechanisms. Such information is important to fully understand the structural basis of the catalysis at each stage of a complete enzymatic cycle, i.e., substrate binding, cleavage, and product release. Moreover, while the critical roles of Mg^2+^ ions are known for stabilization of RPR structure and catalysis, the exact number and precise coordination of metal ions in the active site remain unclear, and with mixed interpretation, among the various RNase P structures reported at relatively low resolution^6–10^, none of which elucidated the structural details of those ions at different reaction states. Lastly, the structures of apoE complexed with pre-tRNA or mat-tRNA from any organism have never been determined. Such structures may shed light on an ancient protein-independent catalytic mechanism of RPR, the only naturally occurring *trans*-acting ribozyme, and provide insight into the evolutionary origins of RNA as a catalyst and its subsequent acquisition of ancillary protein subunit(s).

We report here the apoE structures of *Geobacillus stearothermophilus* (*Gst* RNase P) RNA and its complexes with pre-tRNA (apoES) and product mat-tRNA (apoEP) at resolutions of 2.8–3.5 Å.

## Results

### Structures of RNase P RNA throughout the catalytic cycle

**Fig. 1:**
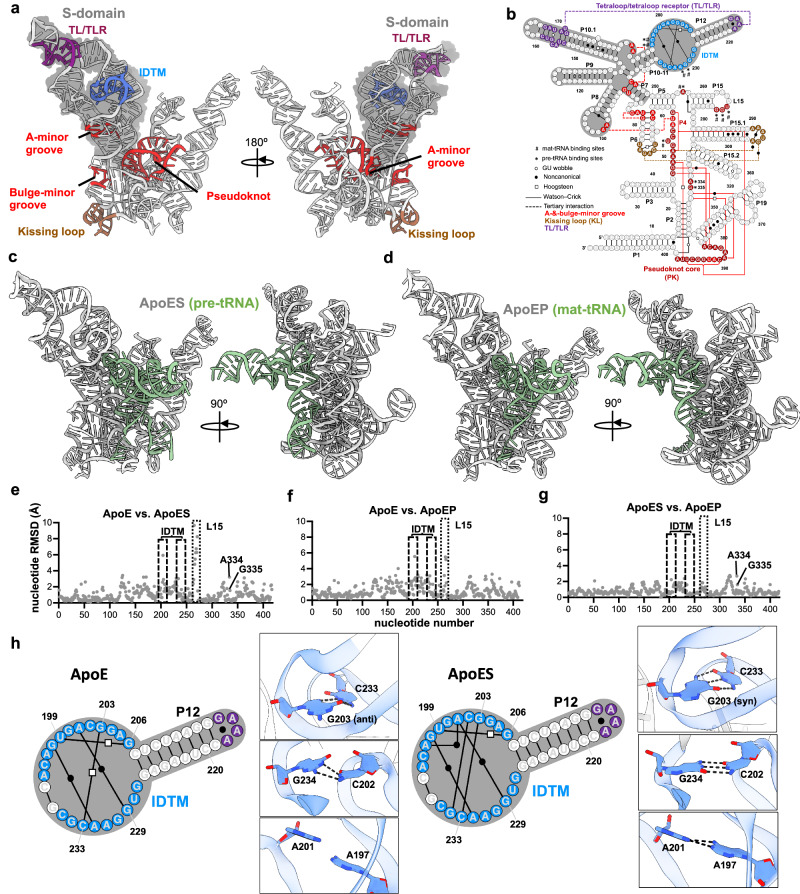
Structures of RNase P aporibozyme (apoE) and in complex with pre-tRNA (apoES) and mat-tRNA (apoEP). **a** The structure of RPR in the apoE state, with key structural elements and tertiary interactions highlighted. The gray-shaded region is the substrate-specificity (S-) domain. The rest is the catalytic (C-) domain. **b** The detailed secondary structure of RPR as it exists in complex with pre-tRNA (apoES), with the same color scheme mapping on the apoES three-dimensional structure shown in (**a**). All long-range interactions are noted with red lines connecting interacting nucleotides or regions. The A-minor groove interaction involving two consecutive adenine bases (A99 and A100) in P8 of the S-domain and the minor groove of P4 in the C-domain is shown as a red broken line. **c**, **d** Two orthogonal views of the structures of apoES and apoEP complexes, respectively. **e**–**g** Per-nucleotide root-mean-square-difference (RMSD) between permutative pairs of the structures, apoE vs. apoES, apoE vs. apoEP, and apoES vs. apoEP, respectively. The dotted boxes highlight regions where significant conformational changes occur. **h** Conformational changes shown in both the secondary and tertiary interactions in IDTM of RPR upon binding of pre-tRNA.

We conducted a systematic structural analysis of apoE by deriving eight cryo-EM structures under different divalent cation conditions (5 or 10 mM Ca^2+^ or Mg^2+^) (Supplementary Fig. 1) and in complex with various pre-tRNAs or mat-tRNA, featuring a well-characteristic divalent metal ion framework for each state (Supplementary Fig. 2). Each of these structures contains a full-length RNase P RNA (RPR), enabling complete and accurate mapping of detailed secondary structure and divalent metal ions in each context. With the three primary structures of apoE and its complexes with pre-tRNA^Gly^ substrate (apoES) and mat-tRNA (apoEP) at 5 mM Ca^2+^ (Fig. 1a, c, d, Supplementary Table 1, Supplementary Figs. 1 and 2), we illustrate the structure of RPR throughout the full catalytic cycle. At the average overall resolution of ~3 Å amongst these structures, all key structural elements and long-range interactions are well defined. The catalytic (C-) domain of RPR exhibits the highest local resolution of ~2.5 Å, whereas the substrate-specificity (S-) domain shows greater mobility and lower local resolution ranging from 3 to 6 Å (Supplementary Fig. 1a), consistent with its dynamic motions described previously^11^. Importantly, the entire S-domain could be modeled for all cryo-EM volumes in this study (Supplementary Fig. 1a, b). In the context of apoE, this is particularly significant, as no complete structure of any RNase P RNA alone has ever been reported. The structure of the S-domain was determined in isolation but was mostly unresolved in the full-length apoenzyme structure^12,13^. In other bacterial apoE structures, the S-domain could not be resolved^13–15^. Even at 5 mM Ca^2+^ (or Mg^2+^), apoE shows structural variability in the S-domain, as well as P19, but its structure could be sufficiently represented as three distinct subclasses (Supplementary Fig. 3a).

In general, RPR maintains a similar overall fold and secondary structure in all three states (apoE, apoES, and apoEP) of the reaction cycle (Fig. 1a–d). This includes several well-defined noncanonical base pairings and tertiary interactions present in all structures (Supplementary Fig. 3b). Moreover, all three structures exhibit the A-minor groove interaction involving two consecutive adenine bases (A99 and A100) in the S-domain and the minor groove of P4 in the C-domain (Fig. 1a). As this is the only direct tertiary interaction between the two domains, the fact that it is observed in the absence of substrate is significant. In our previous study at 1 mM Mg^2+^, this interaction was absent in some conformers of apoE^11^, suggesting that it may be transient in nature and potentially stabilized upon substrate binding. Despite the similar global architecture of RPR in each reaction state, the presence of tRNA (pre-tRNA or mat-tRNA) induces functionally significant structural changes, namely in L15, the loop region harboring key residues A334 and A335, and the interdigitated double T-loop motif (IDTM) (Fig. 1e–h**)**. When substrate is bound, the IDTM exhibits stronger base-pairing interactions, including a rotation in G203 from anti to syn conformation to form a Watson-Crick pair with C233 (Fig. 1h). The IDTM structure, together with the tetraloop/tetraloop-receptor (TL/TLR) interaction, illustrates the critical role the S-domain has in substrate binding and complex stability. This is evidenced by notable improvements in both local resolution and Q-score for the S-domain in the structures of tRNA-bound complexes relative to those of apoE alone, even at 10 mM Mg^2+^ (Supplementary Fig. 1).

### Substrate recognition via six toeholds

**Fig. 2:**
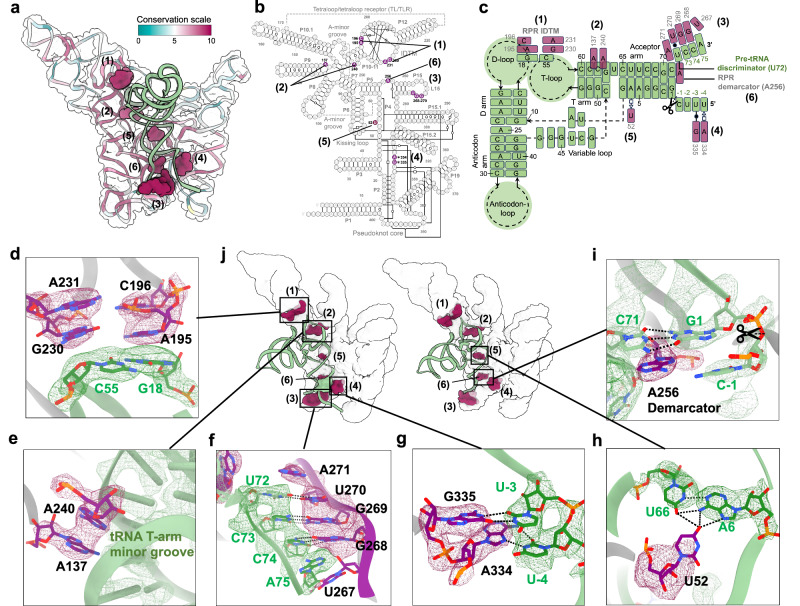
Substrate recognition by RNase P aporibozyme. **a** The locations of the six contacts (toeholds) (red-purple space-filled models) between the substrate (green ribbon diagram) and RPR (semi-transparent gray volume with a ribbon diagram). The regions highlighted in red-purple are highly sequence-conserved among 114 bacterial type-B RNase P sequences. **b** Secondary structure schematic of RPR with nucleotides involved in the six toeholds highlighted in red-purple. **c** Secondary structure schematic of pre-tRNA with nucleotides involved in the six toeholds annotated. The structural details of the six toeholds: RPR IDTM interaction with the pre-tRNA elbow region (**d**); RPR bulge interactions (A137 and A240) with the minor groove of the T-arm of pre-tRNA (**e**); Base-pairing interactions between RPR L15 and the pre-tRNA 3′-trailer and acceptor arm, including discriminator base U72 (**f**); Base-pairing interactions between RPR nucleobases A334 and G335 and the pre-tRNA 5′-leader (**g**); Base-triple interaction involving RPR U52 and the A6:U66 base-pair in the pre-tRNA acceptor arm (**i**); Base-stacking interaction between the demarcator base (A256) of RPR and the nucleobase of the last nucleotide (C71) of pre-tRNA (**j**). Density maps in all panels are shown at 10 standard deviations.

**Fig. 3:**
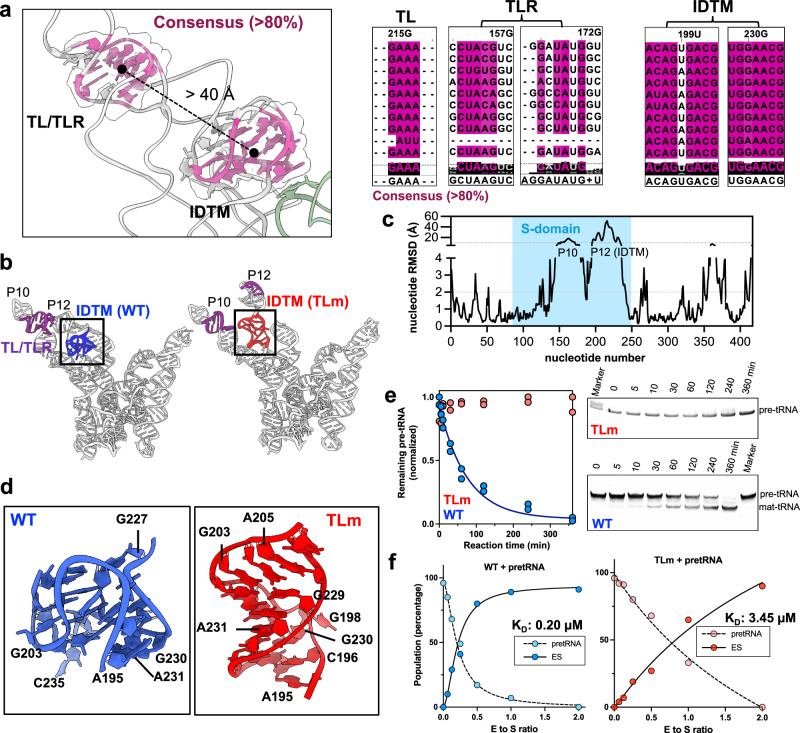
Role of the tetraloop/tetraloop-receptor (TL/TLR) interaction on RNase P structure and ribozyme activity. **a** The structure (left) and sequence conservation among the B-type RNase P RNAs (right) of the TL/TLR and IDTM regions, according to the Rfam database (RF00011). Note that the two structural elements are separated by ~40 Å (left). **b** The structures of wild-type (WT) (left) and TLm (right) apoE. The mutation in TLm abolishes the TL/TLR interaction, resulting in the separation of P10 and P12, which comprise the S-domain, and improper folding of the IDTM. **c** Per-nucleotide RMSD of WT vs. TLm apoE structures, quantifying the large structural differences in the S-domain. **d** Structural switch in the IDTM from the functional well-folded structure in WT (left) to a non-functional helical structure in TLm (right), a result of the disruption of the TL/TLR interaction. **e** Time-course experiments of the hydrolysis of pre-tRNA^Gly^ for WT and TLm apoE, showing that the tetraloop mutation nearly abolishes ribozyme activity. Data are duplicates from independent experiments, *n* = 2. The remaining pre-tRNA substrate bands on a denaturing polyacrylamide gel with pre-tRNA marker (29.7 kDa) were used to determine the hydrolysis rate for RNase P apoE. **f** The thermodynamic population of the enzyme-substrate complex (ES), determined by mass photometry at varied E/S ratios. The dissociation constants (*K*_D_) of ES for WT and TLm RPR are labeled above the inset panels.

**Fig. 4:**
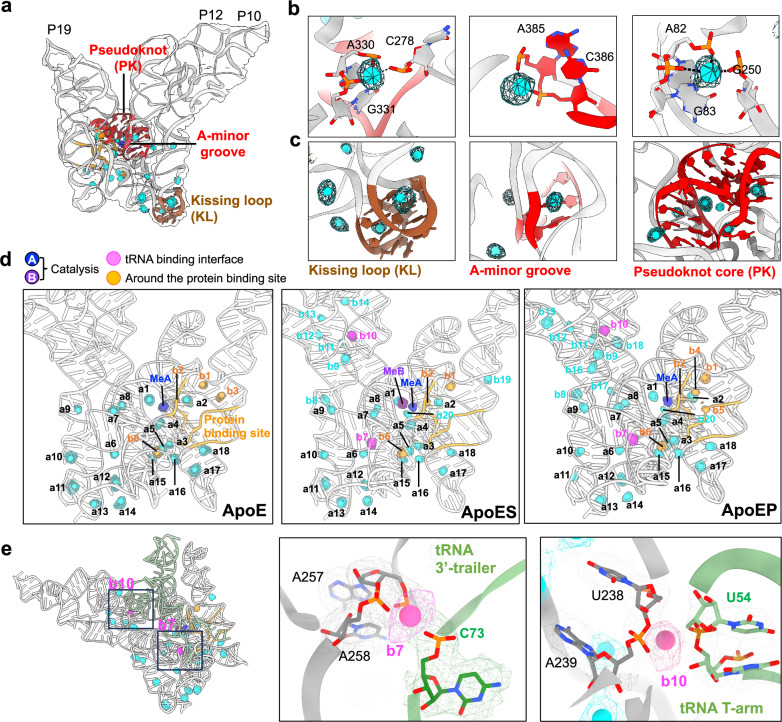
Divalent metal ions observed in the three catalytic states. **a** Overall divalent metal ion distribution in the apoE structure. The divalent metal ions (cyan spheres) shown have an electron density peak height of ≥10 *σ* and a Q-score ≥0.7, and include ions directly coordinated with the RPR backbone phosphates as well as hydrated ions found in the deep major grooves of RPR. **b** Examples of three well-defined ions coordinated by the RPR backbone phosphates. The ion density maps are shown at 10*σ*. **c** Examples of three well-defined hydrated divalent metal ions found stabilizing key tertiary interactions and the pseudoknot core of RPR. The ion density maps are shown at 10*σ*. **d** Divalent metal ions are categorized based on their functional roles. “a” (observed in all states); “b” (observed only in certain states); orange (observed at the rnpA binding interface); magenta (observed only in the presence of pre- or mat-tRNA); blue (the catalytic metal ion A, observed in all states); purple (catalytic metal ion B, observed only in apoES). **e** The two critical divalent metal ions (b7 and b10) involved in the stabilization of the substrate in the apoES and apoEP complexes. ApoE and pre-tRNA are colored in gray and green, respectively. Ions b7 and b10 (magenta) stabilize the tRNA 3′-CCA trailer and T-arm in both complexes, respectively (right panels). The ion density maps are shown at 10*σ*.

Substrate recognition constitutes the first step of the catalytic reaction cycle in any biomolecular reaction system. Our study provides a comprehensive and complete portrait of how this ancient ribozyme recognizes its substrate. In the apoES complex, the pre-tRNA is bound to the RPR through six “toeholds” (Fig. 2a–c): (1) the base-stacking of G18 (D-loop) and C55 (T-loop) in the pre-tRNA elbow region with C196, A195, and G230 in the IDTM in the RPR S-domain (Fig. 2d) a well-known module for tRNA recognition^16^; (2) RPR bulges (A137 and A240) and minor groove interaction with the T-arm of pre-tRNA (Fig. 2e); (3) two Watson-Crick base pairs between C73 and C74 of the pre-tRNA 3′-trailer and G269 and G268 in RPR L15, respectively, and a base pair between pre-tRNA discriminator U72 and RPR U270 via a non-canonical stretched U-U wobble (Fig. 2f); (4) a wobble pair between U-3 in the 5′-leader of pre-tRNA and G335 of RPR (Fig. 2g), and a Hoogsteen pair between U-4 and A334; (5) the insertion of the RPR U52 nucleobase into the major groove of the pre-tRNA acceptor arm, forming a base triple with A6:U66 (Fig. 2h); and (6) the base stacking of A256 in RPR with the last nucleotide (C71) in the pre-tRNA acceptor arm, which acts as a demarcator (Fig. 2i) by segregating the potential base pairing behind C71. Thus, both A256 of RPR and U72 of pre-tRNA play a crucial role in the precise location of phosphodiester bond cleavage. Although three of these toeholds have been known to be critical for RNase P activity^16–20^, the contacts (2), (4), (5), and (6) have never been reported, in part due to the lack of structures at a resolution sufficient to delineate these interactions. The residues comprising the six toeholds are in highly sequence-conserved regions (Fig. 2a), all of which reside in bulges, loops, or internal loops, and none in duplexes (Fig. 2b). In contrast, the interacting nucleotides in the pre-tRNA are located in the D-loop, the acceptor-stem duplex, or in single-stranded regions (Fig. 2c). In apoEP, loss of the interaction between the 5′-leader and A334 and G335 of RPR (toehold 4) likely facilitates the release of the mat-RNA for a complete turnover (Fig. 2a, j).

Interestingly, in the cryo-EM volumes of the apoES complex, we observed a portion of a second pre-tRNA molecule, whose 5′-leader was annealed to that of the first pre-tRNA via sequence complementarity in the region (−14 to −6) that extends beyond the RPR binding interface (Supplementary Fig. 4a, b). To ensure that the structure of the complex is not distorted due to the presence of the second pre-tRNA, we designed two pre-tRNAs that either prevented annealing (non-complementary) or promoted self-annealing (loop-back) in this upstream region (Supplementary Fig. 4c). The complex structures with either pre-tRNA variant showed complete agreement with the 5′-leader conformation (up to −5) in the pre-tRNA complex structure (Supplementary Fig. 4d). These structures visualize how the RNase P could access and cleave partially structured regions between juxtaposed pre-tRNAs on bacterial polycistronic transcripts^21^.

### Roles of the tetraloop/tetraloop-receptor interaction and IDTM

The RPR S-domain includes two key structural elements: the TL/TLR interaction and the IDTM (toehold 1) (Fig. 2). Although the function of the S-domain has been discussed^22^, the structural basis for its regulatory role in RNase P activity has not been demonstrated. The sequences of both the TL/TLR and IDTM regions are highly conserved in B-type RPRs (Fig. 3a), and the IDTM is structurally conserved across many organisms^8–10^ (Supplementary Fig. 5). Despite its conservation and role in stabilization of IDTM in the S domain^12,22^, the TL/TLR does not make direct contact with the substrate (Fig. 2a), and the structural interplay between the IDTM and TL/TLR for regulating substrate recognition is not clear. To test the significance of the TL/TLR interaction in IDTM formation, and the direct impact these key features have on global RPR structure and catalysis, we replaced the tetraloop (GAAA) with “UUUU” (TLm) in P12, abolishing the TL/TLR interaction (Fig. 2b). In the structures of apoE-TLm, the two structural elements P10 and P12, which comprise the S-domain, are completely separated and can adopt multiple conformations (Supplementary Fig. 14, Supplementary Table 2), whereas the remainder of the RPR structure, including the catalytic site^11,23^, remains unchanged (Fig. 3b, c). Abolishing the TL/TLR interaction results in large (>20 Å) movements of P10 and P12 of the S-domain (Fig. 3c), and precludes proper formation of the IDTM. Instead, the IDTM residues adopt a quasi-helical structure with no base-pairing interactions (Fig. 3d). Since the IDTM is the toehold that binds the pre-tRNA elbow region (Fig. 2d), its structure is critical for substrate recognition. Indeed, apoE-TLm does not form a complex with pre-tRNA under cryo-EM grid-preparation conditions, even at a 3-fold excess of substrate, and the TLm nearly abolishes cleavage activity by apoE (Fig. 3e). This is consistent with the pre-tRNA binding mesurements, supporting that the TL mutation weakens the RNase P RNA’s binding affinity for pre-tRNA by 17-fold (Fig. 3f). In summary, although the relative distance between TL/TLR and IDTM is greater than 40 Å (Fig. 3a), the TL/TLR interaction is intimately coupled with the proper folding of the IDTM. This coupling could be considered an allosteric switch for B-type RNase P substrate recognition^12,22^.

### Mapping divalent metal ions

The resolution of the cryo-EM volumes in our study allows us to unambiguously observe the coordination of divalent metal ions in the structures of apoE (Fig. 4a) and its complexes with pre-tRNA and mat-tRNA under various divalent metal ion conditions (Supplementary Fig. 2a). Several ions directly form coordinate bonds with backbone phosphates in the hinge region of RPR, which may play structural roles (Fig. 4b). The majority of the metal ions, however, are hydrated, and found in the RNA major grooves (Fig. 4c). Hydrated ions are those whose distances from the nearest atom in RPR are too far to be direct ion coordinations^24^. Nevertheless, they appear to play important stabilizing roles, particularly in maintaining tertiary interactions in RPR, such as kissing loops, A-minor groove interactions, and the pseudoknot (Fig. 4c). Some of the ions could not be identified unambiguously. However, given the much greater affinity of divalent ions and the fact that no ions showed any strong indication of being monovalent (Supplementary Table 3), all ions in the structures were modeled as Mg^2+^ or Ca^2+^, based on buffer conditions (“Methods”). Nevertheless, our results provide a map of the divalent metal ions in RPR, laying a foundation to decipher both their structural and catalytic roles.

For the detailed analysis of metal ions throughout the catalytic cycle, we include only those visible in the cryo-EM volumes at a contour level ≥10 standard deviations (SD) (Supplementary Fig. 2a) and with a Q-score ≥0.7 (Supplementary Fig. 2b), all of which are found in the regions with a local resolution better than 2.8 Å (Fig. 4d, Supplementary Figs. 1 and 2). This resulted in a total of 40 metal ions among all eight structures (Supplementary Fig. 2). Understandably, these ions are distributed mostly in the C- domain, where the structure is most rigid and well resolved **(**Supplementary Figs. 1 and 2). The metal ions were then classified into three groups (Fig. 4d). Group “a” ions (a1 to a18) are ubiquitously observed in all states; group “b” ions (b1 to b20) are observed only in certain states; and the two metal ions denoted MeA and MeB are the catalytic ions. MeA (Fig. 4d, blue sphere) is observed in all apoE, apoES, and apoEP structures. In contrast, MeB is observed only in the apoES complex (Fig. 4d, middle panel, purple sphere). The absence of MeB in apoEP suggests that this ion may stabilize and leave with the product strand. Ions b1 to b6 (Fig. 4d, orange spheres) are close to the protein(rnpA)-binding site. Ions b7 to b20, on the other hand, are seen only in the presence of tRNA (apoES or apoEP), many of which are involved in substrate binding (Fig. 4e, far-left panel). Two such ions, b7 and b10 (Fig. 4e, magenta spheres), located at the RPR-substrate interface, are important for stabilizing the apoES complex, as they form several direct coordinate bonds with RPR and C73 and U54 of the tRNA 3′ trailer and T-arm, respectively (Fig. 4e, right two panels).

### ApoE uses two metal ions for catalysis

**Fig. 5:**
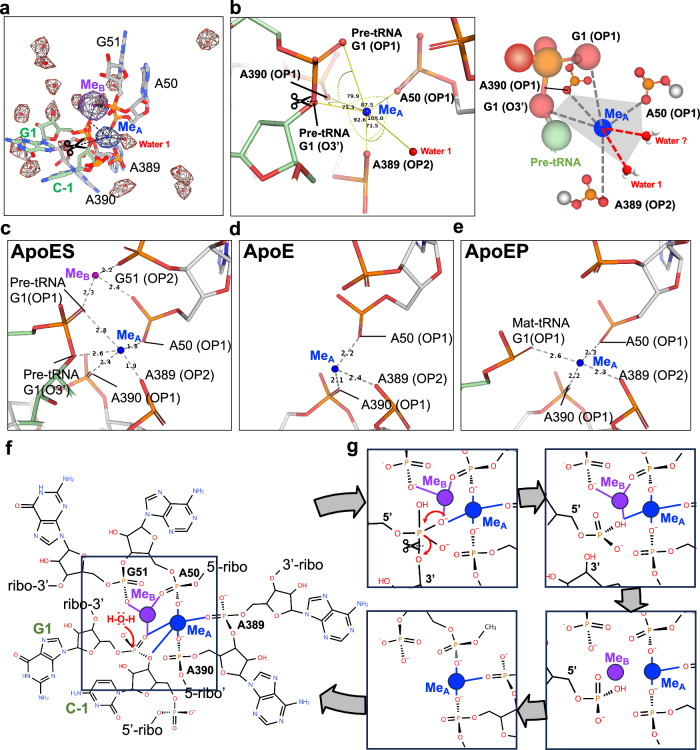
Metal-ion-mediated ribozyme catalysis. **a** Active-site coordination network facilitated by two metal ions (MeA, MeB), as observed in the catalytic site of apoES. Cryo-EM density for the two catalytic metal ions (MeA, MeB) and the water molecule is shown in mesh, contoured at 10 ${{{\boldsymbol{\sigma }}}}$. **b** Pentagonal bipyramid coordination geometry of MeA, consistent with that of Ca^2+^, as observed in the catalytic site of apoES (**a**). Comparison of catalytic metal ions observed in the active sites of apoES (**c**), apoE (**d**), and apoEP (**e**). MeB only appears when the pre-tRNA substrate is bound (**c**). **f** Two-metal-ion mechanism of RNase P aporibozyme proposed based on the apoES catalytic site structure. MeA (blue) coordinates RPR (A50, A389, A390) and pre-tRNA (G1) backbone phosphates. MeB (purple) coordinates RPR (A50 and G51) and pre-tRNA (G1) backbone phosphates. The ion-coordinated network induces a partial positive charge on the phosphorous atom of G1, facilitating nucleophilic attack by a water molecule to form a pentavalent transition-state intermediate (**g**). **g** Catalytic cycle, moving clockwise from (**f**), of the phosphodiester hydrolysis reaction, from the pentavalent transition-state intermediate stabilized by MeA and MeB (top left), to breaking of the phosphodiester bond, to dissociation of MeB along with the cleavage products, to the apoE initiation state (bottom left). All density maps are shown at 10*σ*.

RNase P catalysis involves tightly coordinated metal ions in the active site. Unlike many small and *cis*-acting ribozymes, where divalent metal ions play an indirect role^25–27^, the catalytic metal ions of the trans-activating RNase P ribozyme play a direct role in the nucleophilic substitution reaction during the hydrolysis of the phosphodiester bond at the cleavage site^28^. The structures presented in our work provide direct structural evidence that RNase P in the absence of its protein component utilizes two metal ions (Fig. 5a) for catalysis. Five oxygen atoms tightly coordinate MeA with a pentagonal bipyramidal coordination geometry, consistent with 5 mM Ca^2+^ used in this study to prevent catalysis (Fig. 5b). The equatorial plane is formed by RPR pseudoknot nucleotide backbone phosphates A50(OP1) and A389(OP2), and pre-tRNA G1(O3′). The pre-tRNA G1(OP1) and A390(OP1) occupy two axial positions. Two water molecules could occupy the remaining positions on the equatorial plane (Fig. 5b), one of which (denoted as water 1) was unambiguously identified in the sharpened cryo-EM volume of the highest resolution apoES structure of 2.78 Å (Fig. 5a, b and Supplementary Fig. 6). Water 1 is 5 Å from the scissile phosphorus. In the presence of Mg^2+^, however, which exhibits shorter bond distances and octahedral geometry, it is plausible that water 1 represents the reaction nucleophile. Moreover, MeA is present throughout the entire catalytic cycle and at different concentrations of divalent metal ions (Fig. 5c–e, Supplementary Fig. 2a, blue sphere), indicating that substrate is not required for recruitment or proper coordination of this ion. MeB, on the other hand, is observed only when pre-tRNA is bound (Fig. 5a, c, purple sphere), indicating that its proper coordination is substrate dependent. Indeed, MeB is coordinated by the nucleotide backbone phosphate of G1(OP1) of pre-tRNA, and by G51(OP2) and A50(OP2) of RPR (Fig. 5c). According to the two-metal-ion mechanism, the cleavage of the phosphodiester scissile bond at the pre-tRNA cleavage site occurs only when both metal ions are in position. After the cleavage reaction, MeB dissociates from the active site with the cleavage products, as demonstrated by its absence in all apoE and apoEP structures (Fig. 5d, e). This suggests that the three-bond coordination geometry observed in apoES is important for stabilizing MeB (Fig. 5c), which becomes labile after pre-tRNA cleavage.

We then compared the two catalytic metal ions in apoES (B-type RNase P) from this study with those in the structure of bacterial A-type RNase P in complex with rnpA protein, mat-tRNA, and the 5′-leader (holoEP), which is the only other bacterial RNase P structure available with modeled catalytic ions^8^. MeB is observed in both contexts with similar coordination (Supplementary Fig. 7a, b). Interestingly, however, MeA in the A-type RNase P holoEP structure was not identified in the original report, even though there is a significant peak of electron density at the exact position of MeA after our reexamination (Supplementary Fig. 7b)^8^. Furthermore, we found both MeA and MeB at similar positions in the catalytic sites of protein ribonucleases, such as protein-only RNase P (PROP)^29,30^, RNase H^31,32^, and DNA exonuclease^33^ (Supplementary Fig. 7c, d). These analyses not only corroborate our findings but also suggest an evolutionary consensus role for these two catalytic metal ions in both primordial RNA and contemporary protein contexts. Taken together, the comparative analysis indicates that RNase P uses two metal ions (MeA and MeB) for catalysis in the absence of RNase P protein component, rnpA, and likely a third metal ion (MeC) in the presence of rnpA, which was identified in the A-type complex structure (Supplementary Fig. 7b). This may explain, in part, the enhanced activity that the protein provides and why the apoenzyme is so inefficient, despite the substrate and active site being in the proper configuration.

In the context of the apoenzyme, MeA appears to bind with very high affinity, as this ion was observed in the catalytic site across all structures presented in this work (Supplementary Fig. 2a). In the presence of substrate (apoES), MeA directly coordinates the O3′ atom of the phosphodiester scissile bond of pre-tRNA to stabilize the cleavage site (Fig. 5b, c). This, together with the fact that RNase P activity is dependent on both pH and magnesium concentration^11,34^, suggests that the substrate-dependent MeB plays the more direct catalytic role in the one-step nucleophilic substitution (S_N_2) hydrolysis via a water molecule (Fig. 5f). The recruitment of MeB in the catalytic site is thus a rate-limiting step for the complete cleavage reaction cycle (Fig. 5g) and, in tandem with MeA, lowers the activation energy to form the pentavalent transition-state intermediate. In the context of the RNase P holoenzyme, the activation energy is possibly lowered even further through the stabilizing effects of the rnpA protein and the recruitment of a third metal ion, MeC.

## Discussion

Although it has been more than 40 years since the RNase P RNA was demonstrated to be the catalytic component of this ribozyme^1,35^, no complete structure of the aporibozyme alone or in complex with a substrate has ever been reported. The structures of apoE and its complexes (apoES and apoEP) described here provide insights into the structural basis for protein-independent catalysis by a *trans*-acting ribozyme, unlike *cis*-acting ribozymes^36,37^. Since RNase P is the only *trans*-acting ribozyme identified in nature, this finding is both intriguing and significant. It suggests a possible ancestral, protein-independent two-metal-ion mechanism for substrate-assisted catalysis.

Studies of DNA polymerase^38–40^ and protein nucleases^29^ provided structural evidence of a third catalytic metal ion. Additionally, three-metal-ion mechanisms for catalysis have been proposed for DNA polymerase^38–40^ and Group I and II introns^28,41,42^. However, it is still unclear whether the use of three ions for catalysis is universal among these families of enzymes. In the case of RNase P holoenzyme, the ancillary protein-enhanced activity possibly evolved by introducing a third metal ion. Although a very labile third metal ion could be involved in apoE activity, no such metal was detected in any of our cryo-EM volumes, at an active-site resolution of approximately 2.5 Å.

Both RPR and the ribosomal RNA large subunit (rRNA LSU) are naturally occurring catalytic components of ribonucleoprotein (RNP) complexes that can recognize tRNA^1^. The similarity between these two systems lies in their reliance on RNA–RNA recognition principles. Both utilize a combination of structural complementarity, base stacking, hydrogen bonding, and electrostatic interactions to recruit and position the tRNA substrates accurately. Both RPR and rRNA LSU recognize tRNA molecules by exploiting the highly conserved L-shape of tRNA structure. The L1-stalk in rRNA LSU recognizes the E-site tRNA *via* base stacking interactions resembling the interactions between the IDTM in RPR and the tRNA elbow region (Fig. 2d and Supplementary Fig. 8a, b). In addition, the 3′-CCA trailer of A-site tRNA pairs with the highly conserved GG dinucleotide on the P loop in the peptidyl transferase center (PTC) in rRNA LSU, similar to how it pairs with L15 in RPR (Fig. 2f and Supplementary Fig. 8c). This common strategy highlights an intriguing case of convergent evolution of tRNA recognition mechanisms employed by ribozymes that catalyze distinct chemical transformations, suggesting that the quintessential structural features of tRNA have been conserved and may have, in turn, shaped multiple fundamental cellular processes associated with tRNA and amino acid metabolism. Altogether, our results illustrate the structural basis for RPR-central catalysis. The common structural features in both RPR and rRNA LSU appear consistent with the central hypothesis of the ancient RNA world, in which RNA molecules alone catalyzed biochemical reactions.

## Methods

### RNase P RNA sample preparation

RNase P RNA was transcribed in vitro in transcription buffer (20 mM potassium-HEPES buffer, pH 7.5, 25 mM MgCl_2_, 1 mM Dithiothreitol) for 3 h with recombinant T7 phage RNA polymerase and double-stranded DNA template amplified by polymerase chain reaction (PCR) from a full-synthesized plasmid pUC18. This plasmid encodes the full-length RNase P RNA sequence from bacterial strain *Geobacillus stearothermophilus* (GenBank access number: M19021.1) with an upstream T7 RNA polymerase promoter sequence, GGATCCAGCTCGAAATTAATACGACTCACTATA. After in vitro transcription (IVT), the magnesium pyrophosphate precipitation was removed by centrifugation at a spin rate of 16,600 × g for 10 min using a high-speed benchtop centrifuge. The final concentration of 200 mM sodium chloride (NaCl) was then added to the IVT solution for overnight refolding at 4 °C before further purification. The refolded RNA was subjected to a Fast Protein Liquid Chromatography (FPLC) apparatus (GE HealthCare ÄKTA™ pure) and purified by a nondenatured method^43^ through size-exclusion chromatography (SEC) column (HiLoad^TM^ 16/600 Superdex^TM^ 200 increase). The column was equilibrated with the SEC elution buffer (25 mM Tris-HCl, pH 7.5, 100 mM NaCl, 5 mM CaCl_2_ or MgCl_2_) before purification, and the monomeric RNase P RNA molecules were eluted with a flow rate of 0.5 mL/min and separated from aggregation species. Eluting RNase P RNA was detected by absorbance at 280 and 260 nm; peak fractions were collected based on the SEC chromatogram and stored at 4 °C.

### Mutagenesis for RNase P tetraloop mutation

The DNA template of the RNase P tetraloop mutant was generated by site-directed mutagenesis. The two primers designed for mutagenic PCR are GGAGCTCTAAGGTTTTCCTTAGAGGTGG (sense) and CCACCTCTAAGGAAAACCTTAGAGCTCC (anti-sense), for the replacement of the GAAA tetraloop with four consecutive pyrimidine bases, UUUU, which prevents the tetraloop/tetraloop receptor interaction. The mutagenesis PCR was performed using high-fidelity *Taq* DNA polymerase (Platinum®, Invitrogen Thermo Fisher Scientific), with a 95 °C denaturation step for 1 min, followed by 30 amplification loop-back cycles (95 °C denaturation for 40 s, 62 °C annealing for 40 s, and 72 °C elongation for 3 min). The PCR product was transformed into TOP10 *Escherichia coli* competent cells (Invitrogen Thermo Fisher Scientific) for the colony screening. The DNA sequence of the RNase P tetraloop mutation was confirmed by capillary electrophoresis DNA sequencing (Quintarabio, USA).

### Precursor and mature tRNA sample preparation

Bacterial cognate precursor (pre-) tRNA^Gly^ DNA template harboring an upstream T7 RNA promoter, followed by a 14-nucleotide-long 5′-leader sequence and a 6-nucleotide-long 3′-trailer, CCAAUA, was fully synthesized by Ultramer^TM^ service (Integrated DNA Technologies, USA). PCR generated a double-stranded DNA template for in vitro transcription, producing a 92-nucleotide-long pre-tRNA. Production of mature tRNA^Gly^ (mat-tRNA) that lacks the 5′-leader sequence follows the same expression scheme. The in vitro transcribed pre-tRNA^Gly^ and mat-tRNA were natively purified using a size-exclusion chromatography (SEC) column (HiLoad^TM^ 16/600 Superdex^TM^ 200 increase) with 1 mM Ca^2+^ or Mg^2+^ buffer (25 mM Tris-HCl pH 7.5, 100 mM NaCl, 1 mM CaCl_2_ or MgCl_2_), depending on the purpose of the experiments.

The non-complementary (nc) and loop-back (LB) pre-tRNA variants are designed to prevent the hybridization of the 5′-leader sequence (Supplementary Fig. 4c). The 5′-leader of nc-pre-tRNA is GGCUCUUAACUUUC in place of the cognate 5′-leader sequence, which can prevent the 5′-complementary pairings between the two pre-tRNAs.

Following a similar rationale, a loop-back hairpin sequence, GGAUCCGGAUCCUUUUGGAUCCGGAUCCCUUUC, in LB-pre-tRNA, was designed to prevent the 5′-complementary pairings between the two pre-tRNAs via intramolecular hairpin formation within the 5′-leader.

### Enzymatic assay of 5′ processing of precursor tRNA

The activity assays for RNase P ribozyme (apoE) were conducted at a final enzyme concentration of 2 μM. The purified pre-tRNA^Gly^ was incubated with apoE at an enzyme-to-substrate molar ratio of 1:2. The reactions were conducted at 37 °C in the presence of 25 mM Tris-HCl, pH 7.5, 100 mM NaCl, and 5 mM MgCl_2_.

For the time-course experiments of hydrolysis of pre-tRNA^Gly^ by RNase P (Supplementary Fig. 9), the reaction at each time point was terminated by adding the denaturing gel loading buffer and boiled at 85 °C for 5 min. Samples were analyzed on an 8 M urea Tris-borate-EDTA (TBE) denaturing preparative 8% polyacrylamide (29:1 acrylamide: bis-acrylamide) gel and stained with SYBR^TM^ Gold (Invitrogen Thermo Fisher Scientific). 5′-processing of pre-tRNA^Gly^ via RNase P RNA produces a 78-nucleotide-long mature tRNA^Gly^ product that can be separated from remaining pre-tRNA^Gly^ by denaturing polyacrylamide gel electrophoresis (PAGE). Quantification was performed using the ImageJ software suite (2.14.0), and the data were analyzed using GraphPad Prism (Version 10.6.1). All the experiments were performed in duplicate.

### Liquid chromatography-mass spectrometry

Liquid chromatography-mass spectrometry (LC–MS) experiments were performed on a 6520 Accurate-Mass Q-TOF LC/MS system equipped with a dual electrospray source, operated in positive-ion mode. Samples included 2 μM of the 5′-leader sequences of pre-tRNA and the loop-back (LB) pre-tRNA after RNase P ribozyme digestion. The 5′-leaders were buffer-exchanged in RNase-free double-distilled water solution to remove the buffer salt. The 20 μL supernatant was transferred to glass injection vials for LC–MS analysis. 15 μL supernatant was injected for LC–MS, performed with a TSQ Quantiva triple quadrupole mass spectrometer (Thermo Fisher Scientific) operating in selected reaction monitoring mode with negative electrospray Ionization (ESI) and with a Shimadzu 20AC-XR system using a 2.1 × 100 mm^2^, Charity 2.6 µm Oligo-MS 100 A Phenomenex® C18 HPLC column (Part number: 00D-4479-AN). Data acquisition and analysis were performed using a Mass Hunter Workstation (v.B.06.01). Mass Hunter Qualitative Analysis software (v.B.07.00) with Bioconfirm Workflow was used for presenting and deconvoluting mass spectra. The different ionization states of the deconvoluted mass distribution were shown in Supplementary Fig. 10.

### Cryo-EM sample preparation and data acquisition

RNase P RNA to pre-tRNA molar ratio for the apoES complex formation is 1:3 to the final concentration of 4 mg/mL; the complex was formed with 30-min incubation at 4 °C in the buffer condition containing 25 mM Tris, pH 7.5, 100 mM NaCl, 5 mM CaCl_2_. RNase P RNA to mat-tRNA molar ratio for the apoEP complex formation is 1:4; the complex was formed with 30-min incubation at 4 °C in the same buffer condition.

Quantifoil Au grids (R1.2/1.3, 300 mesh) were glow-discharged on each side for 60 s at 25 mA. Four microliters of sample, ranging from 2.8 to 4 mg/mL, were applied to the carbon side of the grid. After 10 s, the grids were blotted for 3 s in 100 % humidity at 6 °C and plunge-frozen into liquid ethane using a Vitrobot Mark IV (Thermo Fisher Scientific). All data were collected using a Talos Arctica G2 (200 kV) microscope equipped with an X-FEG and a Gatan BioQuantum imaging filter. The movies were recorded in super-resolution mode using a Gatan K3 direct electron detector at a magnification of 100 K (pixel size of 0.405 Å), with 2.5-s exposures at a dose rate of ~15 e^-^/Å^2^/s, resulting in a total dose of ~57 e^-^/Å^2^.

To ensure that the structure and orientation of the 5′-leader are not distorted due to this interaction, we determined the structure of apoE complexed with a pre-tRNA containing a non-complementary (nc) 5′-leader sequence to prevent the annealing of the two pre-tRNA molecules (Supplementary Fig. 4c). In this case, the last eight nucleotides of the leader sequence could not be resolved, which is expected for a single-stranded region with no stabilizing interactions (Supplementary Fig. 4d, right panel). However, the structure of the 5′-leader up to −5 is identical to that of the native pre-tRNA 5′-leader (Supplementary Fig. 4d). Intrigued by the potential stabilizing effect such annealing could have on complex stability and catalysis, we also designed a modified pre-tRNA (LB pre-tRNA), where the native 5′-leader sequence is replaced by a loop-back (LB) sequence that self-anneals in this region to prevent intermolecular base pairing (Supplementary Fig. 4c) while also maintaining any stabilizing effect.

### Cryo-EM data processing

All data processing was performed in cryoSPARC (v4.7.1)^44^. Movies were patch-motion-corrected with a Fourier-crop factor of 0.5, followed by patch Contrast Transfer Function (CTF) estimation. Curated micrographs were denoised, and particles were selected using a template of 2D classes. The particles were extracted and binned to a pixel size ranging from 2.4 to 3.2 Å and subjected to several iterative rounds of reference-free 2-dimensional (2D) classification. The 2D-cleaned particles were used to generate 1–3 ab initio volumes, the best of which was used for non-uniform refinement to align the full stack of particles for 3-dimensional (3D) reconstruction. The particles were then pruned in 3D using one or more iterative rounds of heterogeneous refinement against the refined volume and three or more decoy volumes. The cleaned particle stack was re-extracted with a box size of 512 pixels and a pixel size of 1.03 Å and was split into 2–3 subclasses by 3D classification (without alignment). In most cases, the subclasses only showed significant fluctuations in P19 and, in part, the substrate specificity (S) domain, which could be further subclassified (Supplementary Fig. 11). For simplicity, however, the particle stacks in these cases were kept aligned to a single consensus volume. For all structures complexed with tRNA, the particles containing precursor tRNA (pre-tRNA) or mature tRNA (mat-tRNA) were sorted from those without by 3D classification using a focus mask around the tRNA (Supplementary Figs. 12 and 13).

Applying the same processing workflow as for the wild-type (WT) RNase P apoE (Supplementary Fig. 11), the RNase P RNA tetraloop mutant (apoE-TLm), which lacks the tetraloop/tetraloop-receptor interaction in the S-domain, appears to be more structurally heterogeneous than WT. In this case, 3D classification was applied to subclassify the particles into three different conformer structures, where the two structural elements of the S-domain, P10 and P12, adopt different positions (Supplementary Fig. 14).

The final particle stacks in the aforementioned cases and their corresponding exposures were clustered and split into exposure groups according to beam shift. These particles were then subjected to further non-uniform refinement, including optimization of the per-particle scale, defocus, and per-exposure-group CTF parameters at each iteration. The resulting particle stacks, volumes, and masks were then used for reference-based motion correction (RBMC). In some cases, further pruning could be achieved by heterogeneous refinement (with decoys) and/or 3D classification after RBMC. In these cases, the improved particle stacks and volumes were further refined and used for a subsequent round of RBMC. The particle stacks from RBMC were used for a final round of non-uniform refinement, followed by local refinement using the output mask.

### Model building and refinement

The initial structural model of *Gst* RNase P RNA component (RPR) was built using RNA homology modeling program ModeRNA (2.6)^45^ with the two initial crystal structures (PDB IDs: 2A64^13^ and 1NBS^12^). The full-length *Gst* RPR structure was fitted into the cryo-EM volume, refined, and regularized in real space using Coot (0.9.8.96)^46^. After model fitting and regularization, SimRNA (3.2)^47^ and ISOLDE^48^ were used to minimize the global and local energies of the RNA structures.

To build *Gst* RPR in complex with its tRNA substrate and protein component, the structures of precursor tRNA substrates and mature tRNA products were built with an initial structural model of the bacterial tRNA^Gly^ (PDB ID: 4MGN^49^, chain B). The missing regions of both tRNA^Gly^ and RNase P RNA were modeled and real-space-refined together with *Gst* RPR structure using Coot (0.9.8.96)^46^. The final models of *Gst* RPR and other enzymatic states were refined and validated against their respective unsharpened cryo-EM volumes using Phenix (1.21.2)^50^. Structure and validation statistics, including overall correlation coefficients (C.C.), per-nucleotide C.C. (Supplementary Fig. 15), Fourier shell correlation (FSC) map resolution, all-atom clash scores, and other refinement parameters, are summarized in Supplementary Table 1.

### Metal ion assignment

For building the divalent metal ions, we include only the metal ions whose cryo-EM maps were visible at a contour level ≥10 standard deviations (SD) (Supplementary Fig. 2a) with a cryo-EM resolvability Q-score^51^ ≥0.7 (Supplementary Fig. 2b). Given the coordination distances between metal ions and closest phosphate groups^24^, the directly coordinated metal ions and hydrated metal ions can be further defined (Fig. 4b, c). Given the buffer conditions for each structure, the only probable ions are Na^+^ and Ca^2+^/Mg^2+^. We analyzed metal ions using the CheckMyMetal program^52^ by assessing coordination geometry, contact, distance, B-factor, and occupancy of the metal ions in our structures (summarized in Supplementary Table 3). Although we cannot completely rule out the possibility of monovalent ions like sodium (Na^+^) in some ambiguous cases, given the saturating concentrations of divalent ions in the buffer and that ordered Na^+^ ions are rarely observed or reported in RNA structures, we then model them as divalent ions. All assigned metal ions in our structures exhibit high cryo-EM volume occupancy and Q-scores (Supplementary Fig. 2).

### Water molecule assignment

For mapping water molecules near the catalytic site, we utilized a sharpened map of the apoES in complex with the LB-pre-tRNA substrate, which has the highest map resolution in this study (Supplementary Table 1). The auto-sharpen map module in Phenix (1.21.2)^50^ was used for the cryo-EM map sharpening. For the 2.78 Å map, the overall b-sharpen applied was 71.30 Å^2^. We modeled water molecules in the position at least 20 Å away from the two catalytic metal ions (MeA and MeB), with their cryo-EM maps visible at a contour level of ≥5 standard deviations (SD). We identified a total of 23 water molecules based on these criteria and further validated them by measuring the distance between the water oxygen and the RNA backbone oxygen. All measured distances are in a range from 2.0 to 3.5 Å (Supplementary Fig. 6).

### Mass photometry measurement

The mass photometry experiments were performed on the mass photometer TwoMP (Refeyn Ltd., Oxford, UK). The data acquisition was performed with AcquireMP (version 2023 R1.1) software, and data analysis and figure making were performed with DiscoverMP (version 2023 R1.2) software. High precision cover glasses (coverslips) of size 24 × 50 mm of thickness No. 1.5H (Paul Marienfeld GmbH and Co KG, Lauda-Konigshofen, Germany) and six-well pre-cuts made of CultureWell silicone gaskets (Grace Bio-Labs, Bend, Oregon, USA) were used. The coverslips were rinsed with deionized water and isopropyl alcohol (IPA) (HPLC grade, Sigma-Aldrich Inc., St. Louis, MO) in a sequence water—IPA—water—IPA—water and dried in a stream of ultrapure nitrogen. The instrument calibration was performed with a mixture of β-amylase from sweet potato (Sigma-Aldrich) and thyroglobulin from bovine thyroid (Sigma-Aldrich) dissolved in the same cryo-EM buffer containing 25 mM Tris HCl, pH 7.5, 100 mM NaCl, and 5 mM CaCl_2_. Before placing coverslips on the mass photometer stage, a drop of immersion oil Immersol 518 F (Carl Zeiss Jena, Oberkochen, Germany) was placed on the microscope lens.

The apoE WT and apoE TLm enzymes of 0.2 μM were mixed with pre-tRNA substrate at a series of enzyme to substrate molar ratios (E/Ss), ranging from 0.06 to 2.0. The droplet-dilution procedure of probe preparation was applied. The autofocusing was performed with pure buffer added to the silicone gasket well, then the drop of RNA solution was added and thoroughly mixed by pipette pumping to achieve a final well content concentration of 2 nM. The data acquisition image was set for a regular size. The movie was recorded for 60 s. The data analysis was performed in histogram mode with manually adjusted selection of observed peaks and application of a nonlinear fit with a Gaussian distribution function.

The mass distributions based on number of counts of particles at different E/S ratios (Supplementary Fig. 16) can be used to derive the dissociation constant of RNase P RNA to the pre-tRNA by a nonlinear regression fit by GraphPad Prism (Version 10.6.1) with the One-site specific binding equilibrium model, $[Y]=\frac{{B}_{max }[X]}{{K}_{D}+[X]}$, where *B*_max_ is the saturation plateau at the maximum binding point in the unit of μM; *K*_D_ is the dissociation constant.

### Reporting summary

Further information on research design is available in the Nature Portfolio Reporting Summary linked to this article.

## Supplementary information

Supplementary Information

Reporting Summary

Transparent Peer Review file

## Source data

Source Data

## Data Availability

Cryo-EM reconstructions and structural models for RNase P RNA-alone (apoE), RNA-substrate complex (apoES), and RNA-product complex (apoEP), generated in this study, have been deposited in the Electron Microscopy Data Bank (EMDB) at https://www.ebi.ac.uk/emdb/ and Protein Data Bank (PDB) at http://www.pdb.org, respectively. Accession numbers for EMDB are: EMD-70888 (apoE consensus, 5 mM Ca^2+^), EMD-70891 (apoE class 0, 5 mM Ca^2+^), EMD-70892 (apoE class 1, 5 mM Ca^2+^), EMD-70893 (apoE class 2, 5 mM Ca^2+^), EMD-70896 (apoE consensus 5 mM Mg^2+^), EMD-70897 (apoE consensus 10 mM Mg^2+^), EMD-70933 (apoES pre-tRNA), EMD-70935 (apoEP mat-tRNA 5 mM Ca^2+^), EMD-70936 (apoEP mat-tRNA 10 mM Ca^2+^), EMD-70937 (apoES nc-pre-tRNA), EMD-70940 (apoES LB-pre-tRNA), EMD-70994 (TLm apoE class 0), EMD-70995 (TLm apoE class 1), EMD-70996 (TLm apoE class 2). Corresponding accession codes for PDB are: 9OV3 (apoE consensus 5 mM Ca^2+^), 9OV6 (apoE class 0, 5 mM Ca^2+^), 9OV7 (apoE class 1, 5 mM Ca^2+^), 9OV8 (apoE class 2, 5 mM Ca^2+^), 9OVB (apoE consensus 5 mM Mg^2+^), 9OVC (apoE consensus 10 mM Mg^2+^), 9OWJ (apoES pre-tRNA), 9OWL (apoEP mat-tRNA 5 mM Ca^2+^), 9OWM (apoEP mat-tRNA 10 mM Ca^2+^), 9OWN (apoES nc-pre-tRNA), 9OWQ (apoES LB-pre-tRNA), 9OY2 (TLm apoE class 0), 9OY3 (TLm apoE class 1), and 9OY4 (TLm apoE class 2). Accession codes of PDBs for the initial structural building of RNase P RNA and tRNA: 2A64 (Ribonuclease P RNA from *Bacillus stearothermophilus*), 4MGN (*G. kaustophilus* glyQS T box riboswitch Stem I in complex with tRNA) and 1NBS (Specificity domain of *Bacillus subtilis* ribonuclease P RNA). Source Data are provided as a Source Data file. Source data are provided with this paper.
