# Supplementary material for: Structural basis for protein-free catalysis by ribonuclease P ribozyme
Source: Nat Commun. 2026 Apr 15;17:5209. doi: 10.1038/s41467-026-71597-4 (PMC13254310; doi:10.1038/s41467-026-71597-4)
Supplement: Supplementary file 1 — Supplementary Information [file 41467_2026_71597_MOESM1_ESM.pdf]

Supplementary Information

Structural basis for protein-free catalysis by ribonuclease P ribozyme

Yun-Tzai Lee[1], Maximilia F. S. Degenhardt[1], Ilias Skeparnias[2], Szu-Yun Chen[1], Bapurao A. Bhoge[1], Sergey G. Tarasov[3], Marzena A. Dyba[3], Jinwei Zhang[2], Jason R. Stagno[1], Yun-Xing Wang[1]

[1]Protein-Nucleic Acid Interaction Section, Center for Structural Biology, National Cancer Institute, Frederick, Maryland 21702, USA.

[2]Laboratory of Molecular Biology, National Institute of Diabetes and Digestive and Kidney Diseases, Bethesda, MD 20892, USA.

[3]Biophysics Resource, Center for Structural Biology, National Cancer Institute, Frederick, Maryland 21702, USA.

Corresponding authors: Yun-Xing Wang, wangyunx@nih.gov

Jason R. Stagno, jason.stagno@nih.gov

Jinwei Zhang, jinwei.zhang@nih.gov

Supplementary Figures:

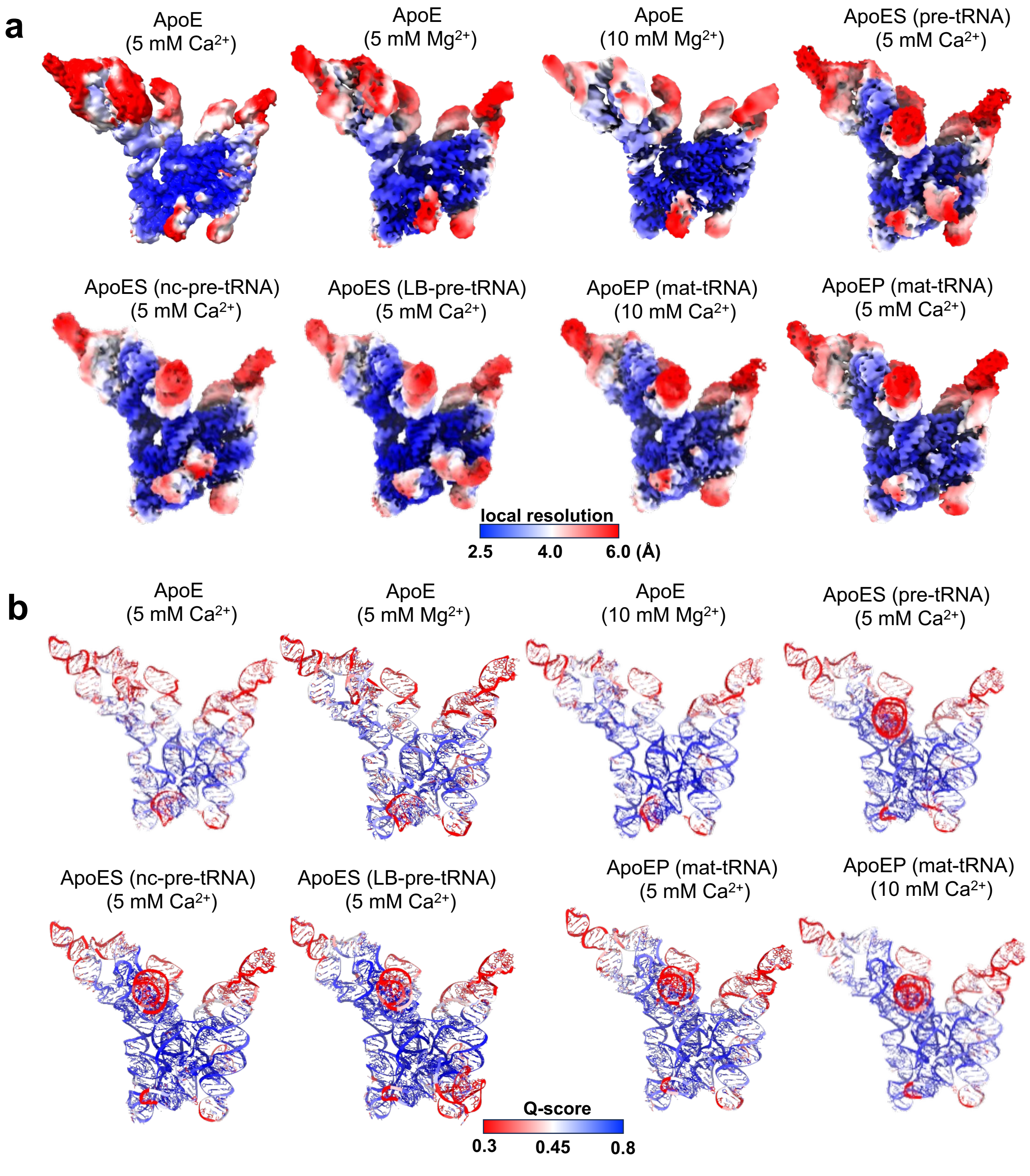

Supplementary Figure 1 | Local structural resolution of cryo-EM volumes and Q-score assessments for corresponding all-atom models. a, Local resolution estimation and b, per-nucleotide Q-score mapping of the cryo-EM consensus volumes of the 8 wild-type RNase P in this study. The local resolution and Q-score were mapped on all volumes from blue, white, and red in an ascending and descending manner on the scale bar, respectively.

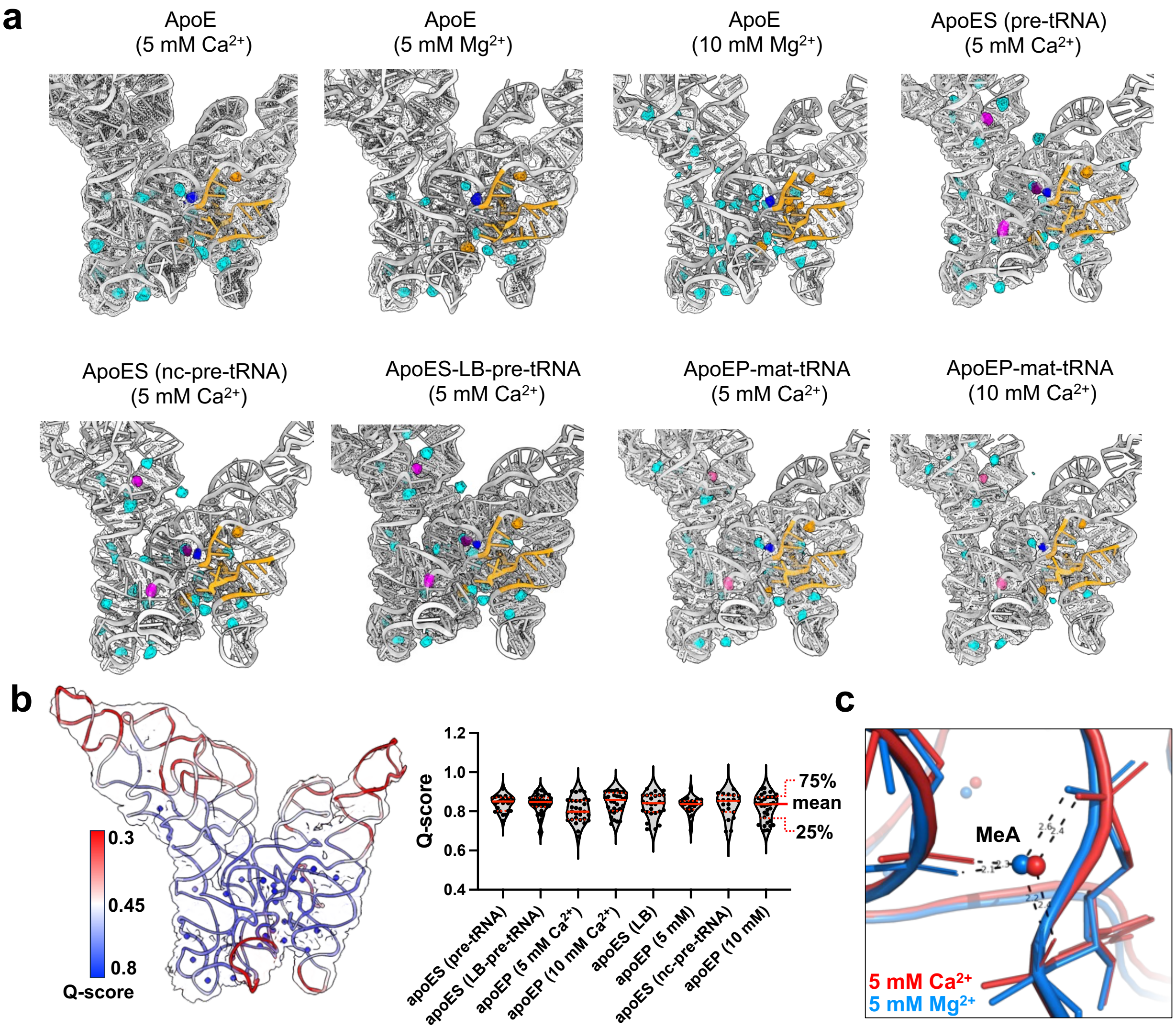

Supplementary Figure 2 | Divalent metal ion density maps and Q-scores in the cryo-EM structures. a, divalent metal ions of all the wild-type RNase P structures, and the structures related to Fig. 4 with the same color scheme, are superimposed with their cryo-EM maps at a contour level threshold of 10 standard deviation (σ). The RNase P protein component, rnpA, binding site is colored orange. b, Q-score of the metal ions in all structures. Statistics of the Q-score distribution of all structures displayed in Violin plots and overlaid with all corresponding data points. c, The position of catalytic metal ion A under varied types of divalent metal ions (Mg^{2+} and Ca^{2+}).

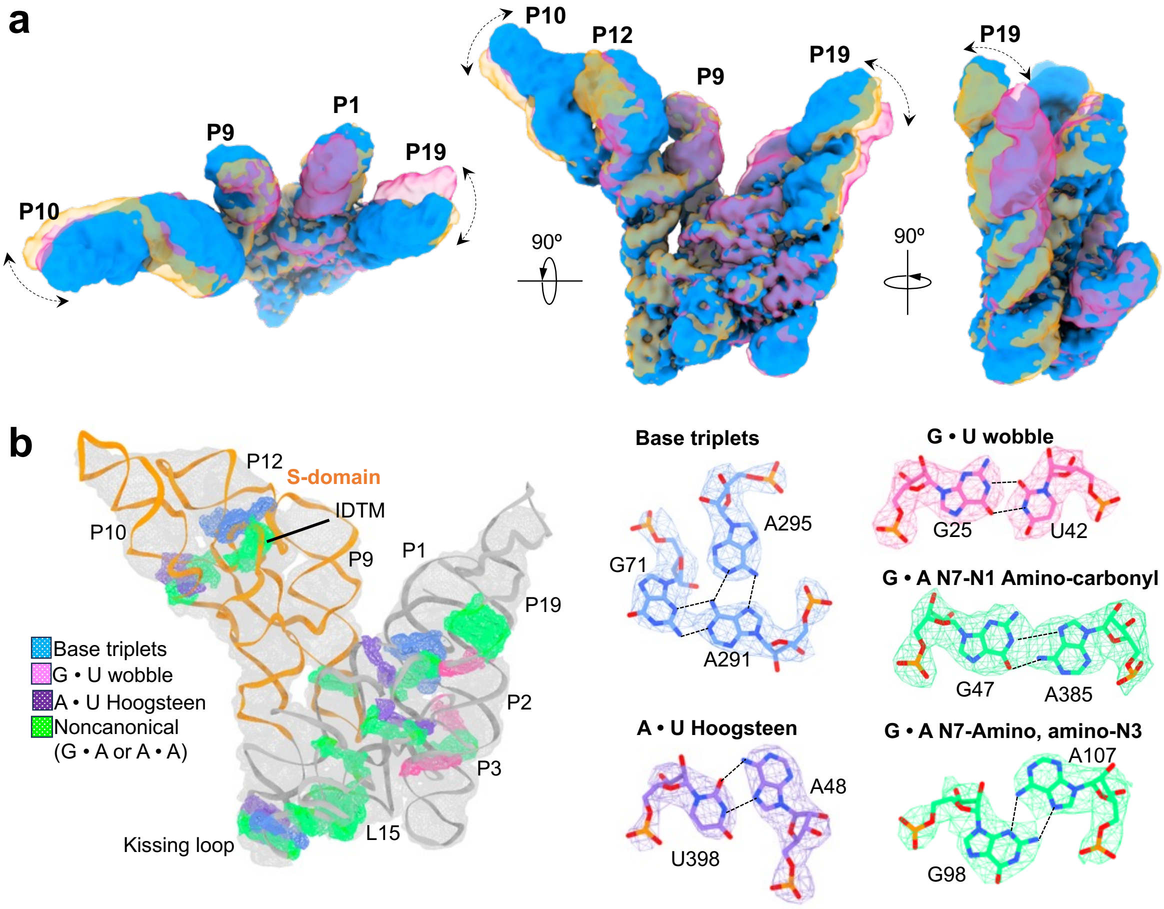

Supplementary Figure 3 | Structural details and variability of RNase P ribozyme (apoE). a, Three distinct conformations of the apoE, with different surface colors, were subclassified and determined using three-dimensional classification in cryoSPARC[1] (Methods). The two conformations of apoE (magenta and orange), aligned against the structure, generated from the majority of particles (blue). b, Local structural details of noncanonical base pairings observed in apoE structure, with different color schemes.

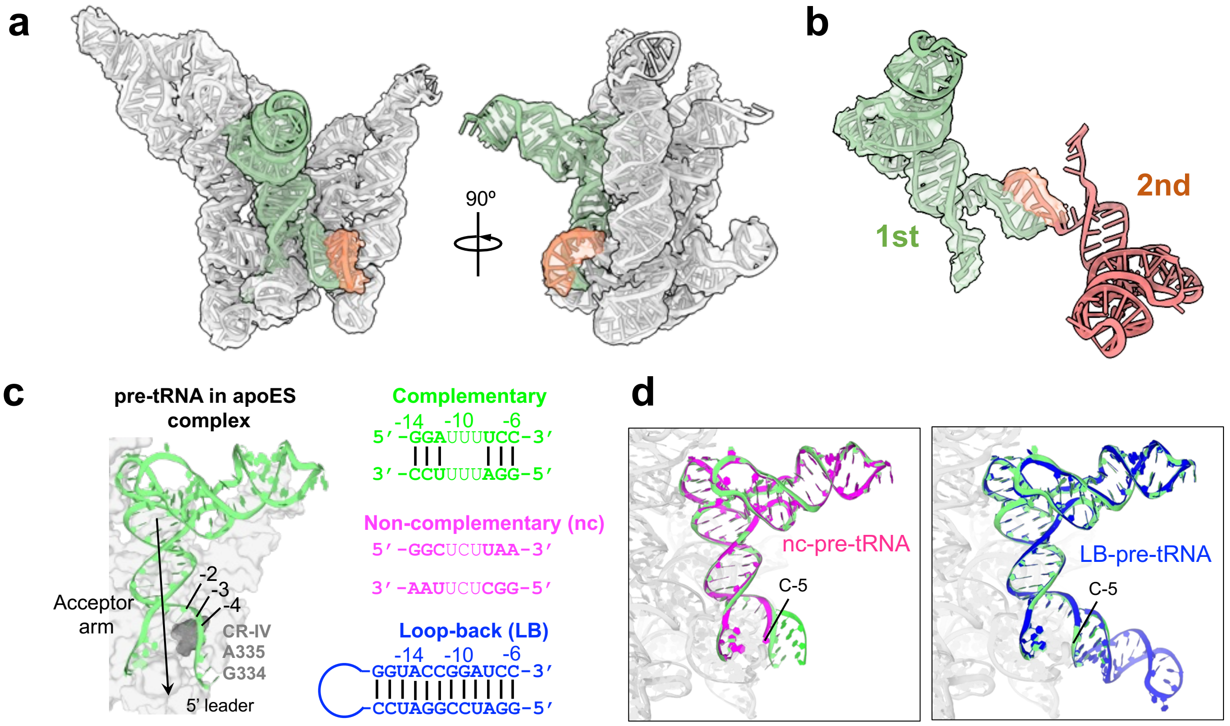

Supplementary Figure 4 | The presence of the second pre-tRNA in RNase P apoES and impact on RNase P apoE structure. a, In the apoES structure, the pre-tRNA 5′-leader conformation promotes annealing with a second pre-tRNA molecule through sequence complementarity in the region upstream (-14 to -6) that extends beyond the RPR-tRNA interface. b, Two pre-tRNA models annealed by the 5′-leader sequence complementarity. c, Cognate pre-tRNA binding interface in apoES and designs of non-complementary (nc) and loop-back (LB) pre-tRNA variants for preventing pre-tRNA dimer annealing *via* 5′ leader. d, ApoES structures show similar interactions with the 5′-leader of pre-tRNA variants (nc- and LB-pre-tRNA) up to C-5.

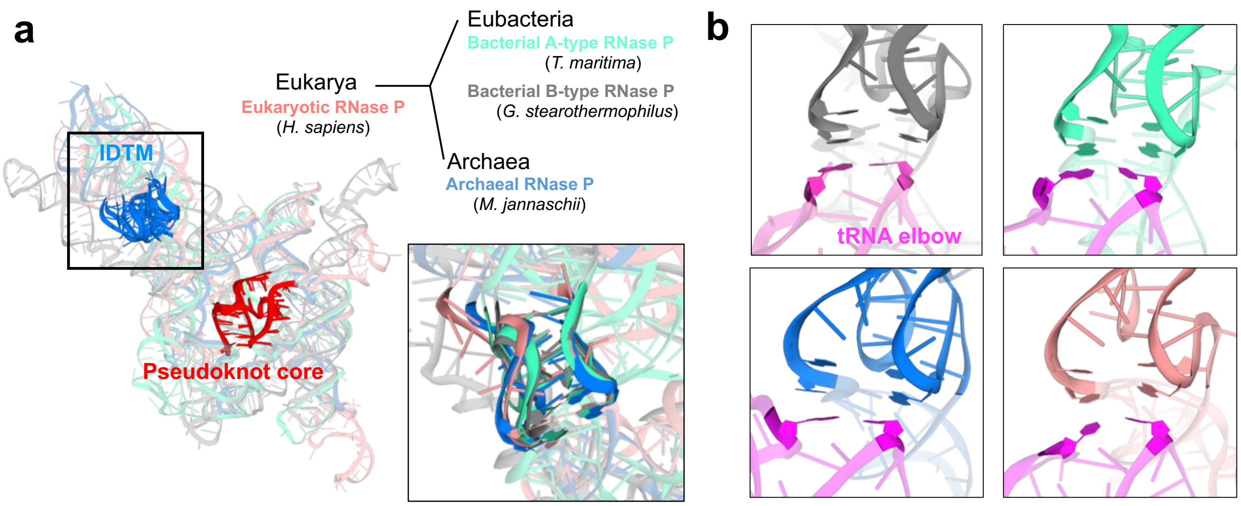

Supplementary Figure 5 | Structural conservation of interdigitated double T-loop motifs (IDTM) of RNase P RNA homologs. a, Structures of RNase P RNA homologs (PDB IDs: 6AHU[2], 6K0B[3], 3Q1R[4]) across the three kingdoms of life aligned against the pseudoknot core region, highlighted in red. Structural alignment of all IDTMs, shown in the inset panel. b, IDTM interaction of the RNase P homologs with the tRNA elbow region (magenta).

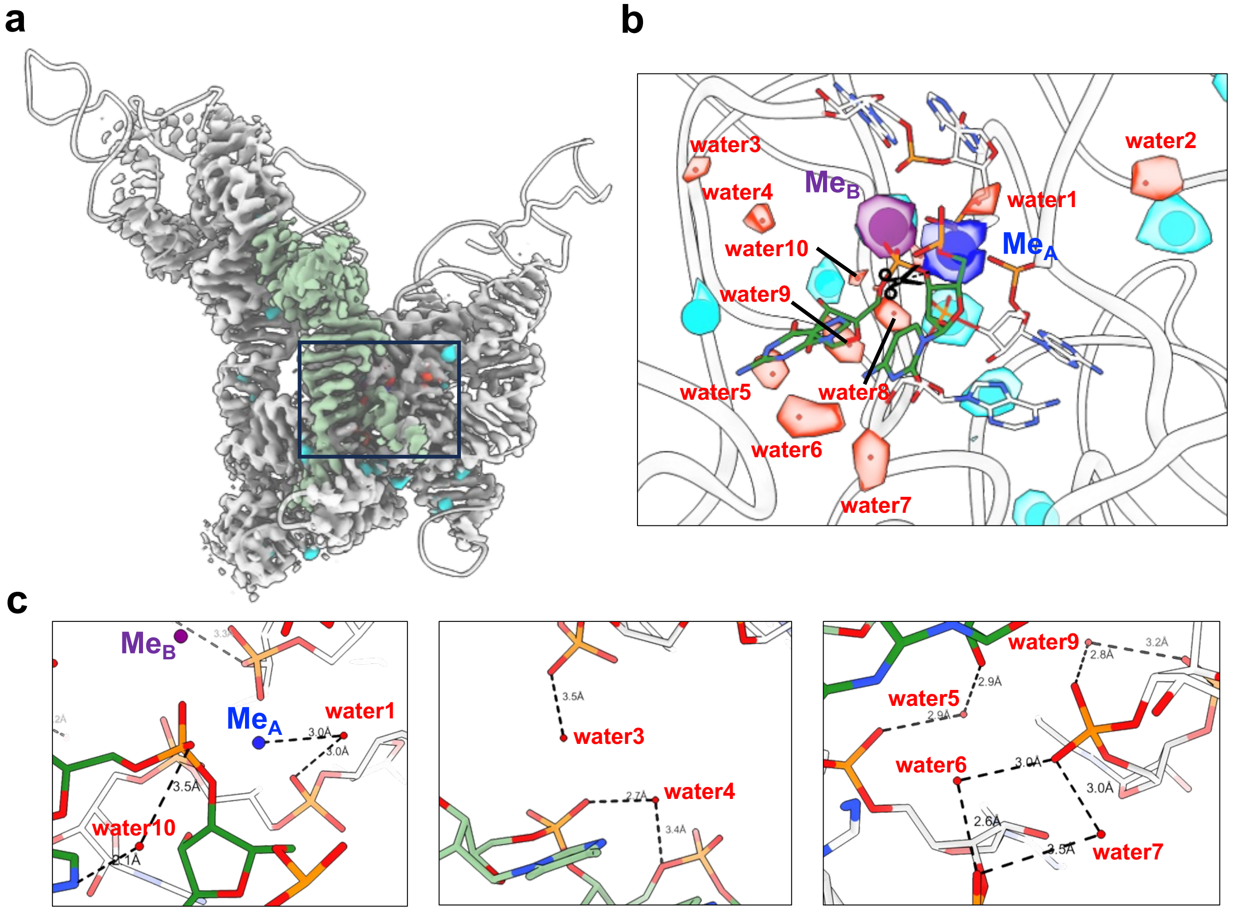

Supplementary Figure 6 | Water molecule mapping using cryo-EM sharpening volume. a, Structures of RNase P apoES structure with water molecules superimposed with the cryo-EM sharpening volume at a contour level of 5 standard deviation (σ). Volume for water molecules highlighted in red surface. b, Divalent metal ions and water molecules in the catalytic center of RNase P. Two catalytic metal ions are colored in blue (Me_A) and purple (Me_B). c, Exemplified distance measurements between water oxygens and RNA backbone oxygens, or the metal ion.

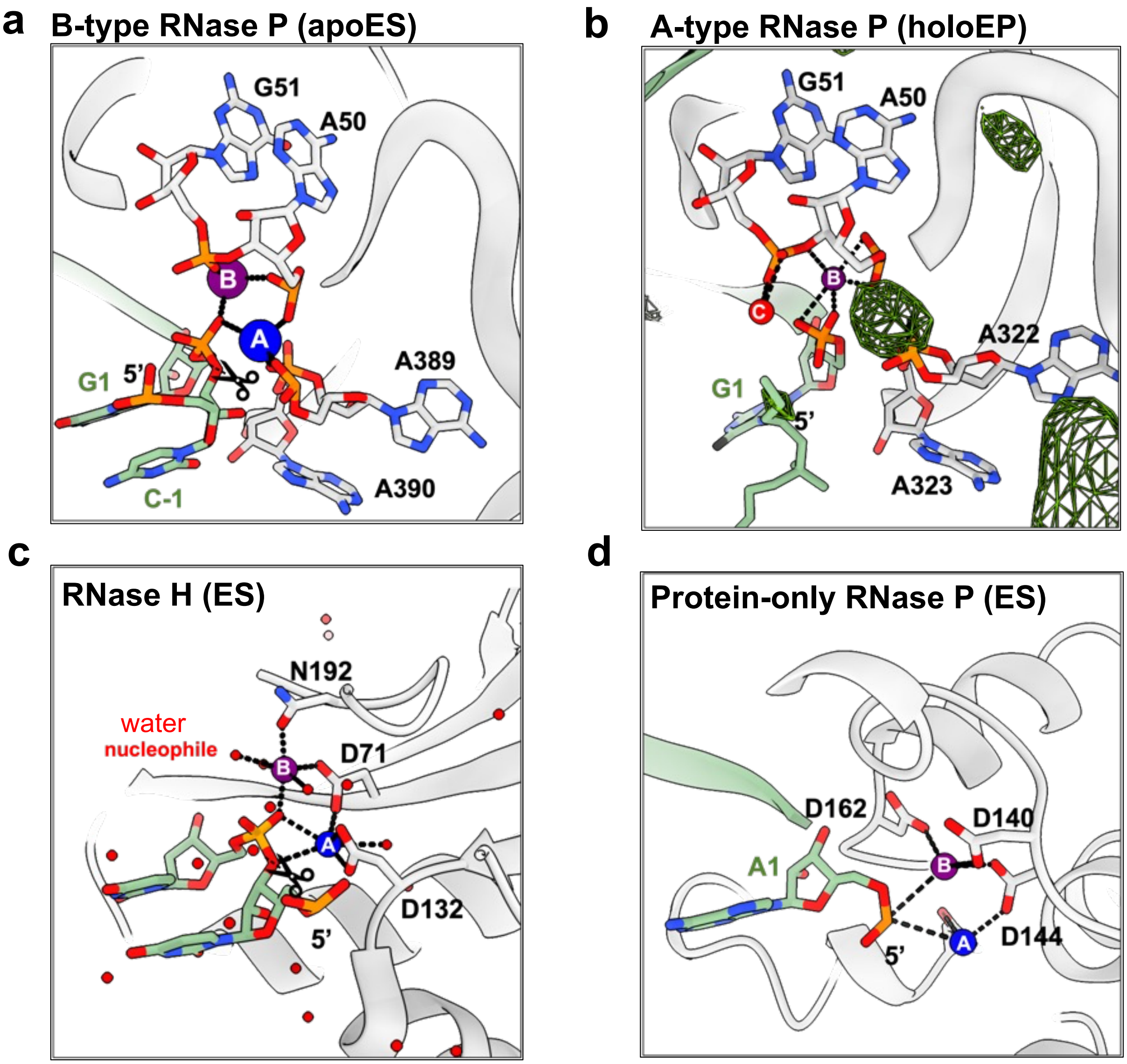

Supplementary Figure 7 | Structural evidence of RNase P and protein ribonucleases, supporting the two-metal-ion mechanism. a, The catalytic site of the B-type RNase P apoES complex structure, determined in this study. The two divalent metal ions, A and B, in the catalytic center are colored in blue and purple, respectively. b, A-type RNase P holoES complex structure determined in a previous study (PDB ID: 3Q1R[4]), and a difference density map of a potential metal ion A shown in green mesh. Two metal ions observed in c, RNase H (PDB ID: 1ZBL[5]) and d, Protein-only RNase P (PDB ID: 8KDA[6]).

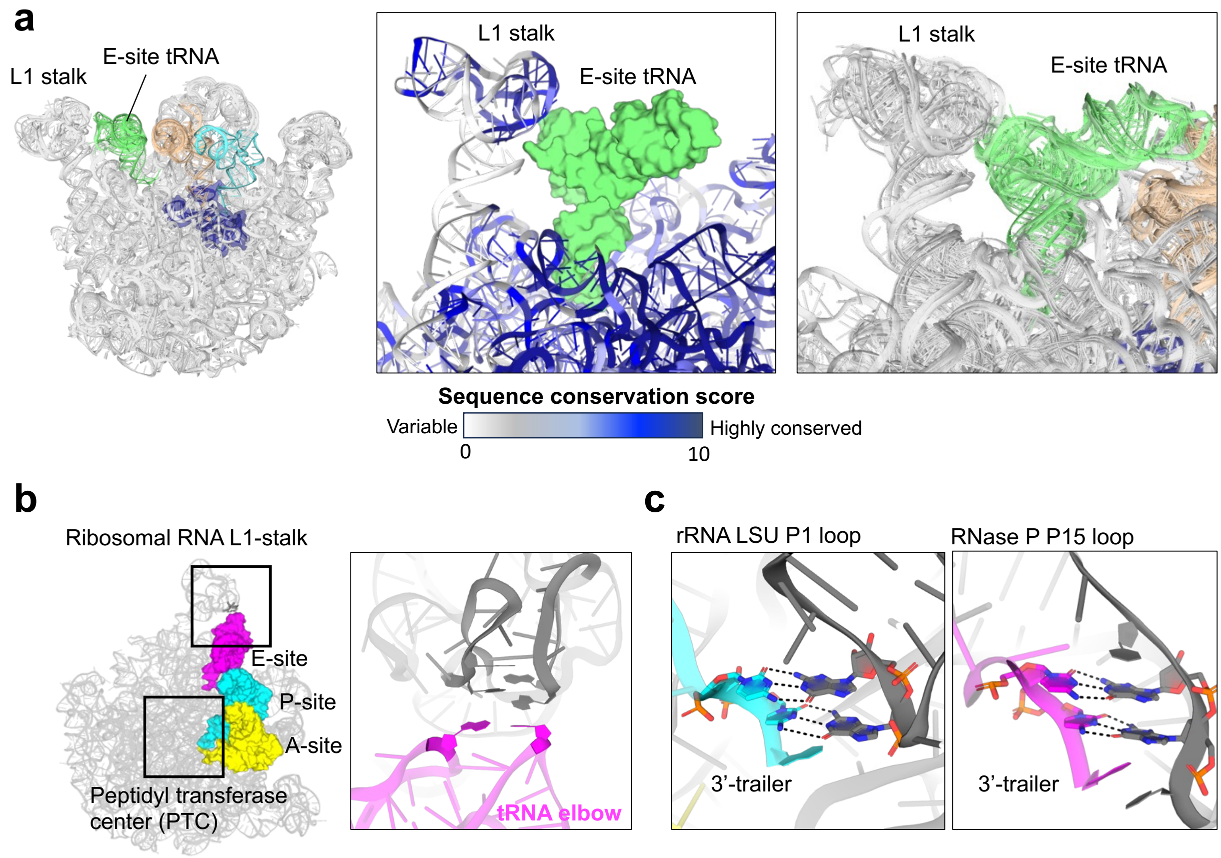

Supplementary Figure 8 | Mechanistic resemblance of tRNA recognition between RNase P RNA and the ribosomal RNA large subunit (rRNA LSU). a, Sequence conservation mapping on rRNA LSU structures. b, Comparison of IDTM-tRNA interaction between RNase P and L1 stalk in ribosomal RNA large subunit (LSU). c, tRNA 3′-trailer interaction with P1 loop and P15 loop in *Gst* RNase P and ribosomal RNA large subunit, respectively.

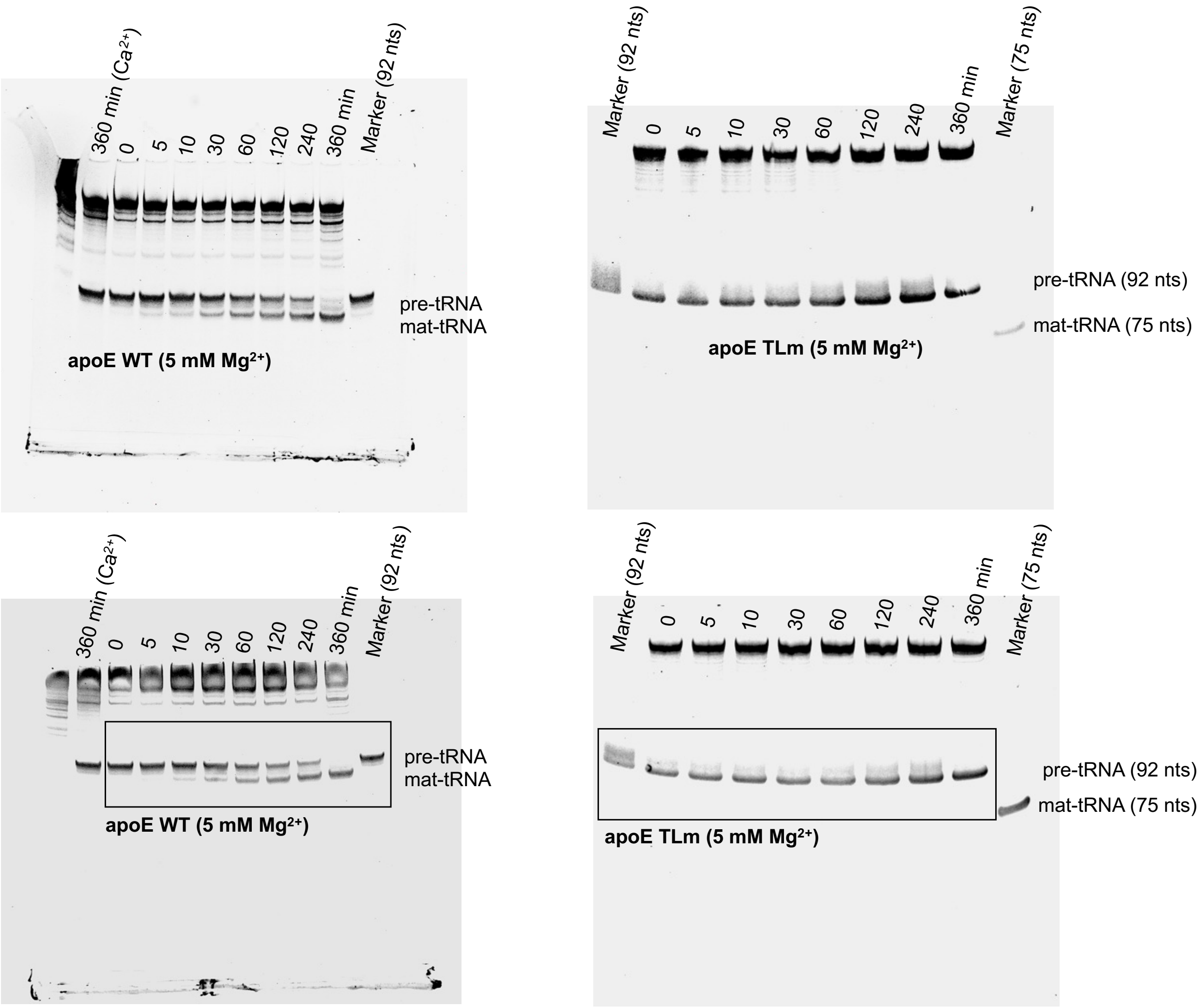

Supplementary Figure 9 | Time-course experiments of nucleolytic cleavage of precursor tRNA by RNase P, analyzed by 8% urea denaturing polyacrylamide gel electrophoresis (PAGE). Impact of the tetraloop mutation (TLm, right) on RNase P ribozyme activity, as compared to WT (left). All reactions were performed at 5 mM Mg^{2+}. For each polyacrylamide gel, the band intensity of the remaining pre-tRNA in each lane was quantified and used to derive the nucleolytic cleavage rates, shown in Fig. 3e. Markers are pre-tRNA and mature tRNA with 27.9 and 24.4 kilodaltons for their theoretical molecular weights, respectively.

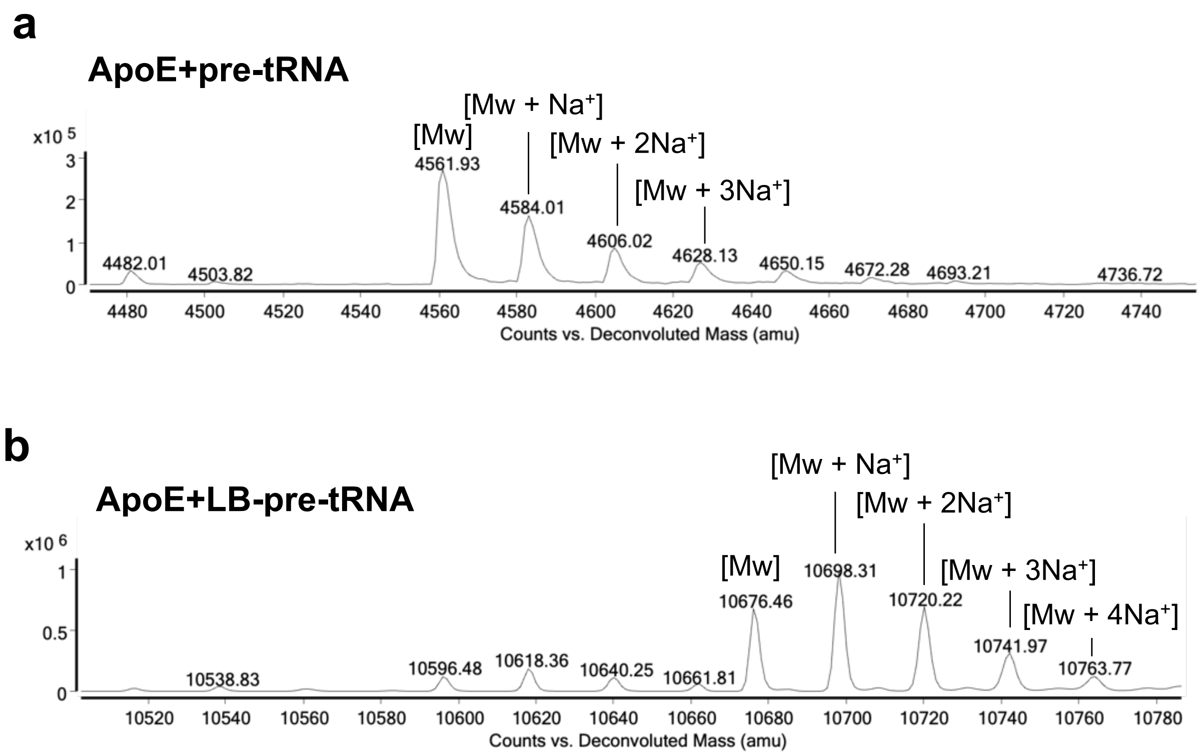

Supplementary Figure 10 | Mass spectrometry assessment to confirm the molecular mass of the 5′ cleavage product of pre-tRNA, produced by RNase P apoenzyme. a, Ionization states and deconvoluted molecular mass distribution of the 5′ cleavage product of pre-tRNA by apoE. b, Ionization states and deconvoluted molecular mass distribution of the 5′ cleavage product of the loop-back (LB) pre-tRNA by apoE.

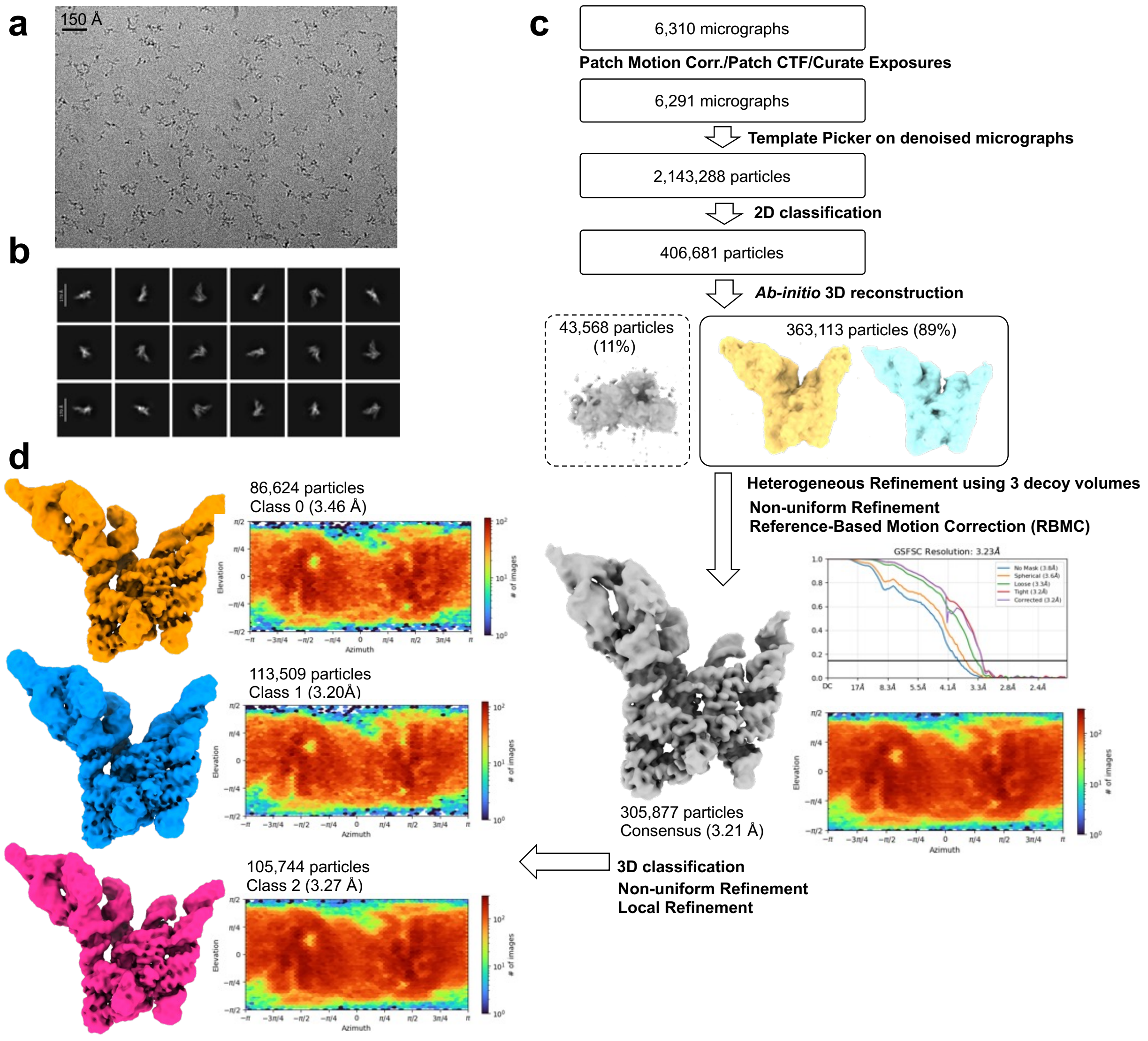

Supplementary Figure 11 | Cryo-EM workflow and analysis of RNase P RNA ribozyme (apoE). a, Representative cryo-EM micrograph after motion correction with a scale bar of 150 Å. b, Representative 2D class averages of extracted particles. c, CryoSPARC[1] workflow from image processing to 3D reconstruction and refinement, including reference-based motion correction (RBMC). d, The consensus volume of apoE was sub-classified into three distinct volumes. The Euler angle distribution of the particle images for the three cryo-EM maps is shown on the right.

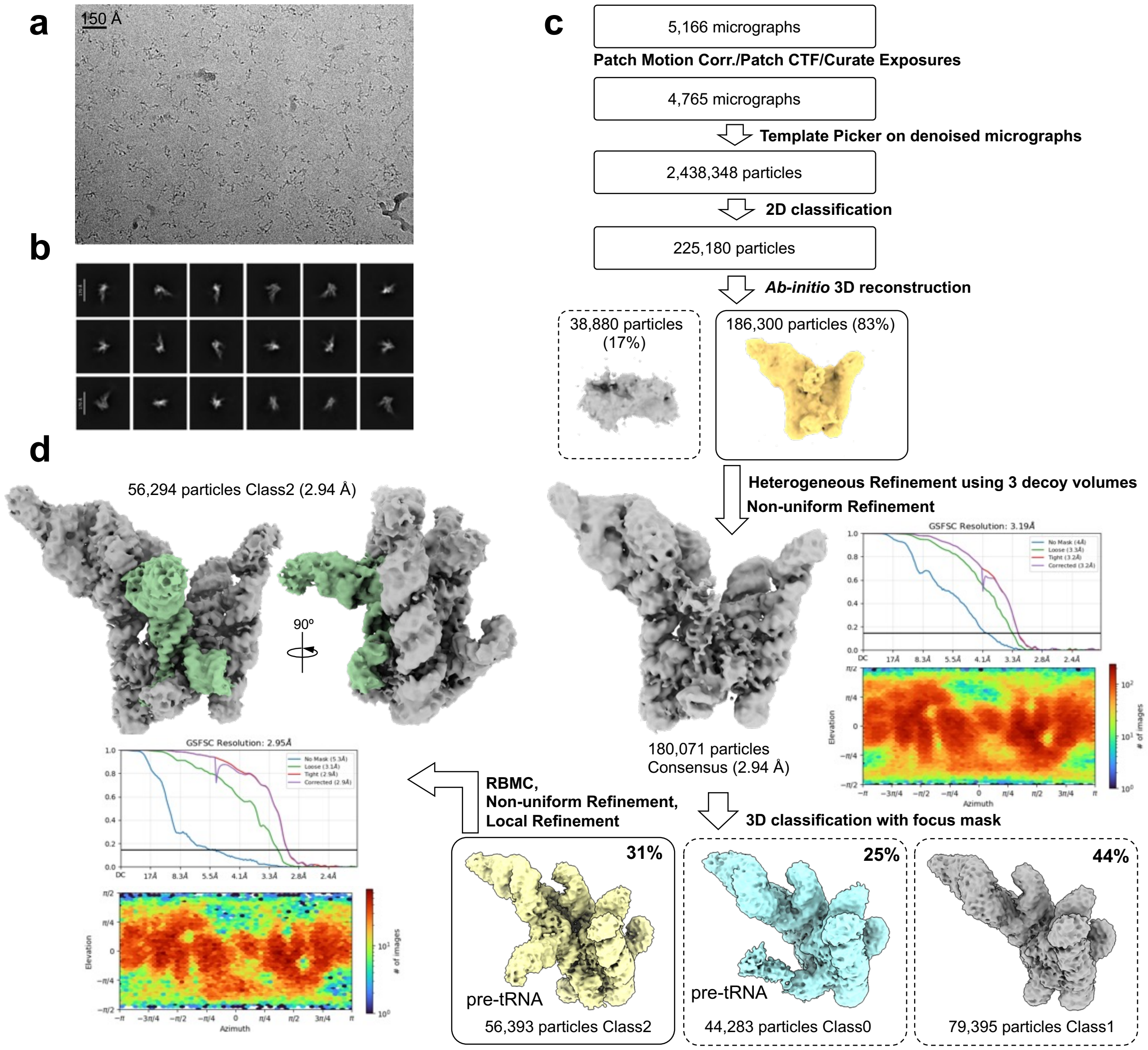

Supplementary Figure 12 | Cryo-EM workflow and analysis of RNase P ribozyme in complex with pre-tRNA (apoES). a, Representative cryo-EM micrograph after motion correction with a scale bar of 150 Å. b, Representative 2D class averages of extracted particles. c, CryoSPARC[1] workflow from image processing to 3D reconstruction and refinement. The consensus volume particle stack was subclassified by 3D classification using a focus mask around the tRNA to purify the particles containing substrate. d, The purified stack of apoES particles was polished by reference-based motion correction (RBMC), followed by non-uniform and local refinement. The Euler angle distribution of the particle images for the cryo-EM map is shown at the bottom.

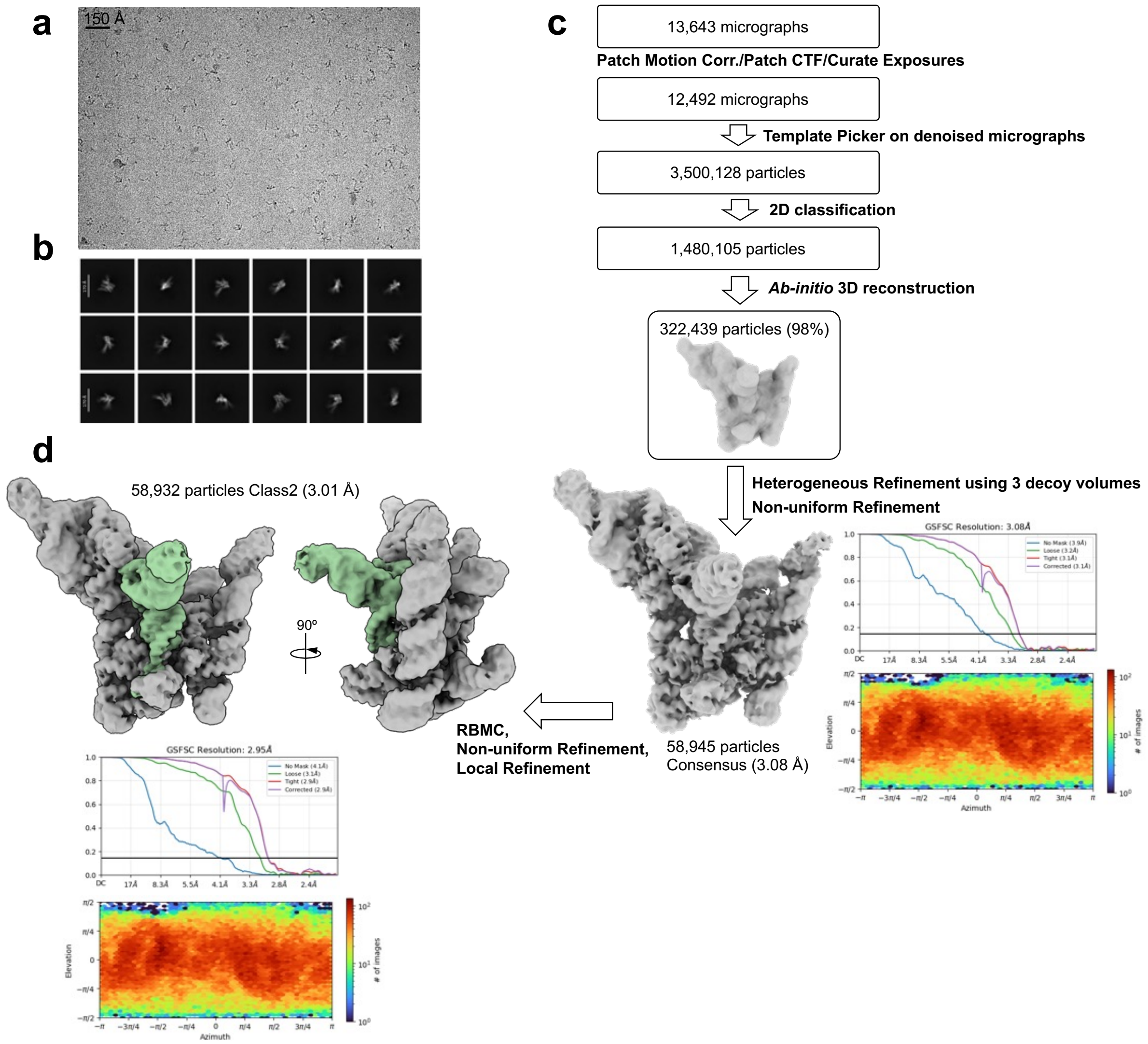

Supplementary Figure 13 | Cryo-EM workflow and analysis of RNase P ribozyme in complex with mat-tRNA (apoEP). a, Representative cryo-EM micrograph after motion correction with a scale bar of 150 Å. b, Representative 2D class averages of extracted particles. c, CryoSPARC[1] workflow from image processing to 3D reconstruction and refinement. The consensus volume particle stack was subclassified by 3D classification using a focus mask around the tRNA to purify the particles containing substrate. d, The purified stack of apoES particles was polished by reference-based motion correction (RBMC), followed by non-uniform and local refinement. The Euler angle distribution of the particle images for the cryo-EM map is shown at the bottom.

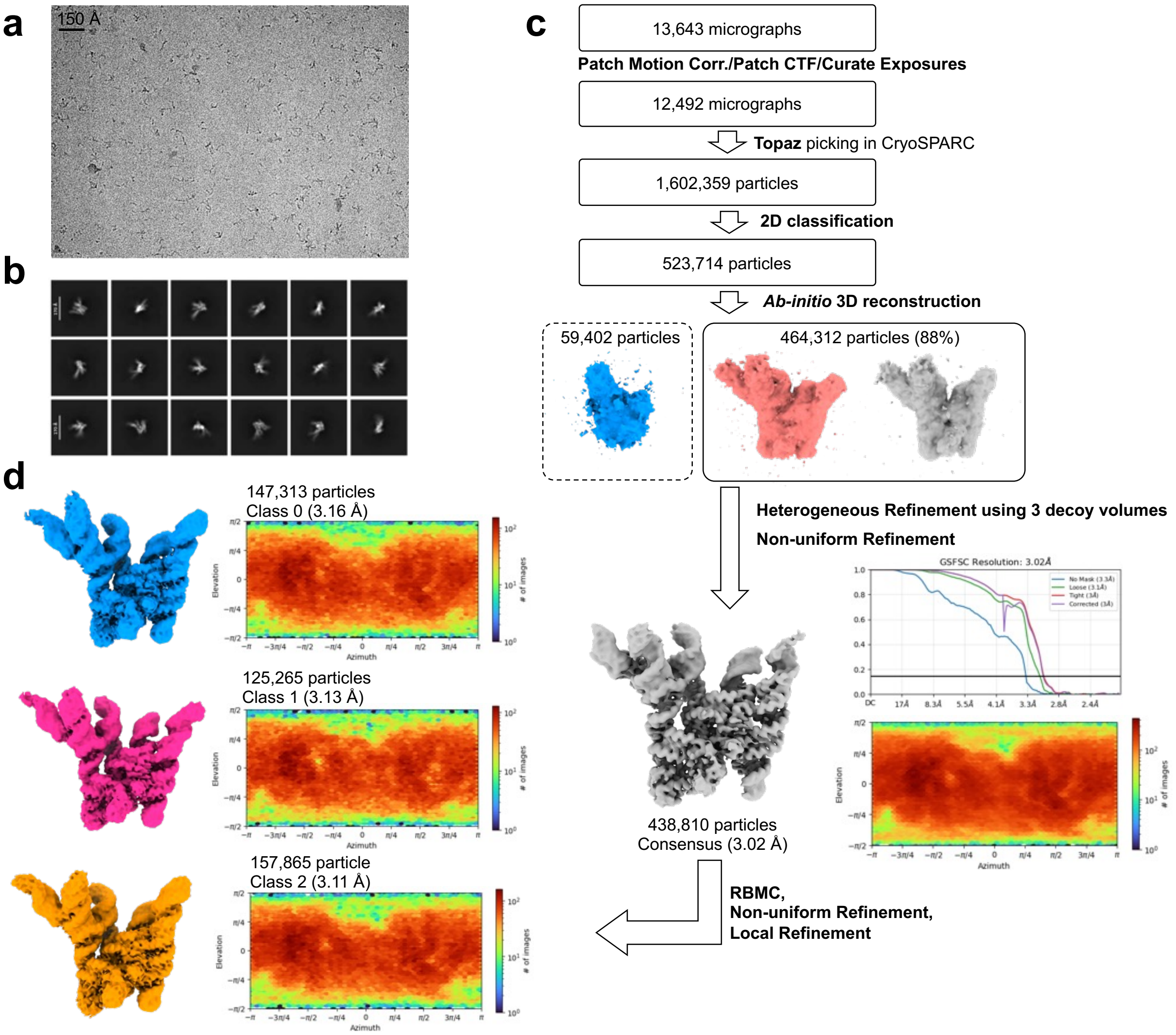

Supplementary Figure 14 | Cryo-EM workflow and analysis of RNase P RNA tetraloop mutant (apoE-TLm). a, Representative cryo-EM micrograph after motion correction with a scale bar of 150 Å. b, Representative 2D class averages of extracted particles. c, CryoSPARC[1] workflow from image processing to 3D reconstruction and refinement. The consensus volume particle stack was subclassified by 3D classification into three distinct sub-volumes with differences in the S-domain and P19. d, The purified stacks of particles were polished by reference-based motion correction (RBMC), followed by non-uniform and local refinement. The Euler angle distribution of the particle images for the three cryo-EM maps is shown on the right.

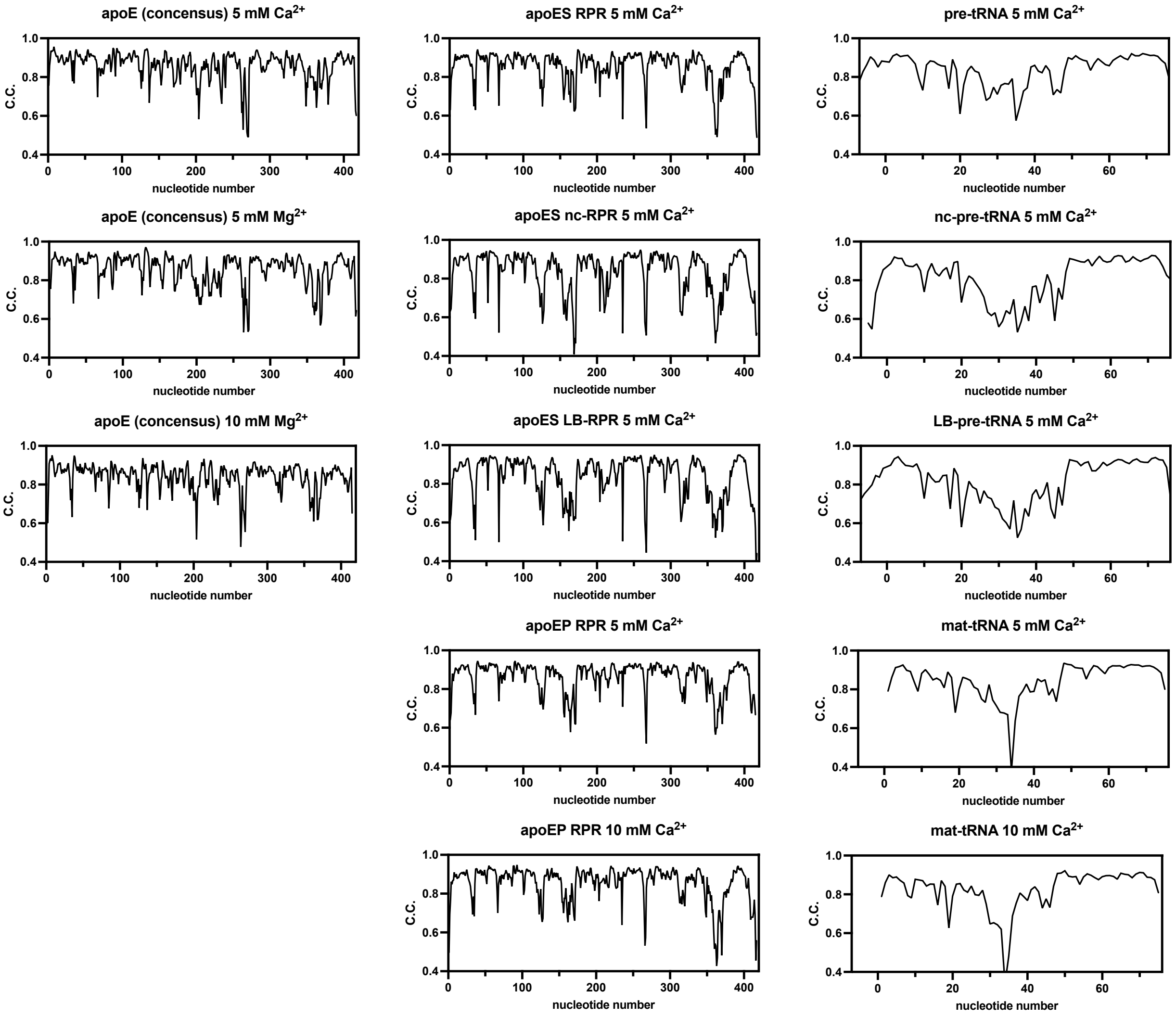

Supplementary Figure 15 | Per-nucleotide correlation coefficient of the RNase P structures and their complex with precursor tRNA (pre-tRNA) and mature tRNA (mat-tRNA) after cryo-EM real-space refinement. a-c, Correlation coefficient (C.C.) plots of the apoE structures in 5-mM Ca^{2+}, 5-mM Mg^{2+}, and 10-mM Mg^{2+}. **d-f,** C.C. plots of the apoES structures in complex with pre-tRNA, nc-pre-tRNA, and LB-tRNA. **g,h,** C.C. plots of the apoES structures in complex with mat-tRNA in 5 mM and 10 mM Ca^{2+}.

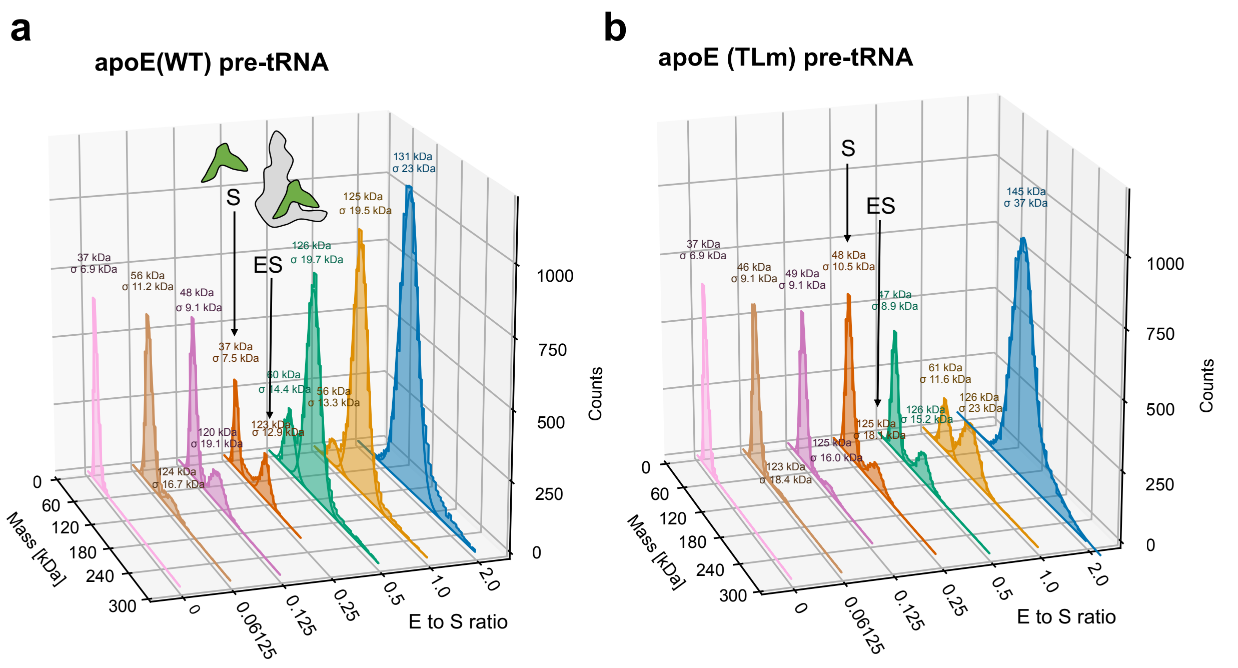

Supplementary Figure 16 | Mass photometry assessments of pre-tRNA substrate binding to apoE wild-type (WT) and tetraloop mutant (TLm). Mass distributions through the number of counts of free pre-tRNA substrate and ES complex at varied E to S ratios, utilized to derive dissociation constants (K_D) of a, apoE WT, and b, TLm to the pre-tRNA, related to Fig. 3f.

Supplementary Tables:

Supplementary Table 1. Cryo-EM data collection, processing, structural refinement, and validation statistics of wild-type RNase P in the catalytic cycle.

	**ApoE (consensus) 5 mM Ca^{2+}**	**ApoE (class 0)**	**ApoE (class 1)**	**ApoE (class 2)**	**ApoES pre-tRNA**	**ApoES (nc) pre-tRNA**	**ApoES (LB) pre-tRNA**	**ApoEP mat-tRNA 5 mM Ca^{2+}**	**ApoEP mat-tRNA 10 mM Ca^{2+}**	**ApoE (consensus) 5 mM Mg^{2+}**	**ApoE (consensus) 10 mM Mg^{2+}**
**Data collection& processing**											
**Magnification**	150,000	150,000	150,000	150,000	150,000	150,000	150,000	150,000	150,000	150,000	150,000
**Voltage (kV)**	200	200	200	200	200	200	200	200	200	200	300
**Electron exposure ($e^-/Å^2$)**	57	57	57	57	57	57	57	57	57	57	55
**Defocus range (μm)**	-0.5 to -1.5	-0.5 to -1.5	-0.5 to -1.5	-0.5 to -1.5	-0.5 to -1.5	-0.5 to -1.5	-0.5 to -1.5	-0.5 to -1.5	-0.5 to -1.5	-0.5 to -1.5	-0.5 to -1.5
**Pixel size (Å)**	1.038	1.038	1.038	1.038	1.038	1.038	1.038	1.038	1.038	1.038	0.824
**Particle numbers**	369,486	86,624	105,744	113,509	180,071	131,843	248,927	58,945	110,322	334,369	1,301,239
**Map resolution (Å)**<br>**FSC threshold**	3.19<br>0.143	3.46<br>0.143	3.27<br>0.143	3.20<br>0.143	2.94<br>0.143	2.86<br>0.143	2.78<br>0.143	2.99<br>0.143	2.95<br>0.143	3.21<br>0.143	2.97<br>0.143
**EMDB**	EMD-70888	EMD-70891	EMD-70892	EMD-70893	EMD-70933	EMD-70937	EMD-70940	EMD-70935	EMD-70936	EMD-70896	EMD-70897
**Refinement**											
**Model composition**<br>**Non-hydrogen atoms**<br>**RNA residue**<br>**Mg^{2+} or Ca^{2+}**	 8988<br>417<br>26	 8989<br>417<br>27	 8988<br>417<br>26	 8987<br>417<br>25	 10866<br>505<br>35	 10717<br>498<br>32	 11309<br>526<br>31	 10598<br>492<br>38	 10591<br>492<br>31	 8989<br>417<br>27	 9003<br>417<br>45
***B* factors ($Å^2$) mean**<br>**RNA**<br>**Mg^{2+} or Ca^{2+}**	 286.04<br>95.32	 281.44<br>87.56	 269.30<br>83.71	 198.45<br>93.91	 198.15<br>99.22	 128.34<br>70.09	 118.30<br>60.80	 159.87<br>95.62	 152.45<br>95.59	 248.98<br>80.37	 184.82<br>87.10
**R.m.s. deviations**<br>**Bond lengths (Å)**<br>**Bond angles (°)**	 0.004<br>0.703	 0.004<br>0.671	 0.004<br>0.709	 0.004<br>0.682	 0.003<br>0.585	 0.003<br>0.596	 0.003<br>0.602	 0.003<br>0.606	 0.003<br>0.606	 0.004<br>0.663	 0.004<br>0.661
**Validation**<br>**MolProbity score**<br>**Clashscore**<br>**CC_{Mask}**<br>**CC_{Box}**<br>**Rotamer outliers (%)**	 2.58<br>7.93<br>0.87<br>0.92<br>0	 2.66<br>9.70<br>0.88<br>0.92<br>0	 2.66<br>9.78<br>0.88<br>0.91<br>0	 2.61<br>8.59<br>0.88<br>0.91<br>0	 2.59<br>8.21<br>0.88<br>0.90<br>0	 2.59<br>7.95<br>0.88<br>0.90<br>0	 2.61<br>8.59<br>0.88<br>0.90<br>0	 2.56<br>7.48<br>0.88<br>0.91<br>0	 2.56<br>7.42<br>0.88<br>0.90<br>0	 2.56<br>7.56<br>0.87<br>0.92<br>0	 2.46<br>5.77<br>0.86<br>0.90<br>0
**PDB**	9OV3	9OV6	9OV7	9OV8	9OWJ	9OWN	9OWQ	9OWL	9OWM	9OVB	9OVC

Footnote: Apoenzyme (ApoE), Apoenzyme-substrate complex (apoES), Apoenzyme-product complex (apoEP), correlation coefficient (CC), Fourier shell correlation (FSC), loop-back (LB),
non-complementary (nc).

Supplementary Table 2. Cryo-EM data collection, processing, structural refinement, and validation statistics of RNase P tetraloop mutant (TLm) in the catalytic cycle.

	TLm ApoE (class 0)	TLm ApoE (class 1)	TLm ApoE (class 2)
**Magnification**	150,000	150,000	150,000
**Voltage (kV)**	200	200	200
**Electron exposure ($e^{-}/Å^2$)**	57	57	57
**Defocus range (μm)**	-0.5 to -1.5	-0.5 to -1.5	-0.5 to -1.5
**Pixel size (Å)**	1.038	1.038	1.038
**Particle numbers**	147,313	125,265	157,865
**Map resolution (Å)**<br>**FSC threshold**	3.14<br>0.143	3.46<br>0.143	3.27<br>0.143
**EMDB**	EMD-70994	EMD-70995	EMD-70996
**Model composition**<br>**Non-hydrogen atoms**<br>**RNA residue**<br>**Mg^{2+} or Ca^{2+}**	 8974<br>417<br>21	 8975<br>417<br>22	 8973<br>417<br>20
***B* factors ($Å^2$) mean**<br>**RNA**<br>**Mg^{2+} or Ca^{2+}**	 271.82<br>59.66	 153.63<br>67.75	 201.38<br>64.33
**R.m.s. deviations**<br>**Bond lengths (Å)**<br>**Bond angles (°)**	 0.004<br>0.716	 0.004<br>0.670	 0.004<br>0.707
**Validation**<br>**MolProbity score**<br>**Clashscore**<br>**CC_{Mask}**<br>**CC_{Box}**<br>**Rotamer outliers (%)**	 2.63<br>8.98<br>0.81<br>0.89<br>0	 2.53<br>6.97<br>0.83<br>0.89<br>0	 2.60<br>8.31<br>0.86<br>0.89<br>0
**PDB**	9OY2	9OY3	9OY4

Footnote: Apoenzyme (ApoE), Apoenzyme-substrate complex (apoES), Apoenzyme-product complex (apoEP), correlation coefficient (CC), Fourier shell correlation (FSC), loop-back (LB),
non-complementary (nc), Tetraloop mutant (TLm).

Supplementary Table 3. Metal ion binding site validation. Metal ion analysis for assigned metal ions using the CheckMyMetal program[7]. Metal ion coordination geometry, contact, distance, B-factor, occupancy, and ranking are listed in the table.

**ApoE (consensus) in 5 mM Ca^{2+}; PBD ID: 9OV3**									
**Metal ID**	***Occupancy***	***Bfactor(env.)*[1]**	***Atomic contacts***	***Valence*[2]**	***nVECSUM*[3]**	***Geometry*[1,4]**	***gRMSD(°)*[1]**	***Vacancy*[1]**	**Ranking (points)**
**CA 1**	1.0	**86.0 (0.0)**		N/A	N/A	**Free**	N/A	N/A	Ca(0), Na(0), Mg(0)
**CA 2**	1.0	*104.0 (133.5)*		N/A	N/A	**Free**	N/A	N/A	Ca(0), Na(0), Mg(0)
**CA 3**	1.0	*126.2 (74.3)*	**O_1**	**0.02**	**1.0**	**Poorly Coordinated**	N/A	N/A	Ca(0), Na(0), Mg(0)
**CA 4**	1.0	85.1 (87.6)	**O_1**	**0.3**	**1.0**	**Poorly Coordinated**	N/A	N/A	Ca(0), Na(0), Mg(0)
**CA 5**	1.0	94.8 (82.1)	**O_1**	**0.2**	**1.0**	**Poorly Coordinated**	N/A	N/A	Ca(0), Na(0), Mg(0)
**CA 6**	1.0	88.1 (91.9)	O_4	**0.17**	**0.66**	Octahedral	*20.2°*	**33%**	Ca(4), Na(4), Mg(4)
**CA 7**	1.0	70.5 (74.4)	**N_1**	**0.05**	**1.0**	**Poorly Coordinated**	N/A	N/A	Ca(0), Na(0), Mg(0)
**CA 8**	1.0	120.0 (135.5)	O_3	**0.7**	**0.7**	Octahedral	*19.7°*	**50%**	Na(5), Ca(4), Mg(4)
**CA 9**	1.0	76.3 (88.5)	**N_1**	**0.05**	**1.0**	**Poorly Coordinated**	N/A	N/A	Ca(0), Na(0), Mg(0)
**CA 10**	1.0	76.5 (82.5)	*O_2*	**0.6**	**0.68**	Octahedral	6.5°	**66%**	Mg(4), Ca(3), Na(3)
**CA 11**	1.0	89.3 (85.9)	**O_1**	**0.3**	**1.0**	**Poorly Coordinated**	N/A	N/A	Ca(0), Na(0), Mg(0)
**CA 12**	1.0	69.4 (78.9)	O_3	**0.9**	**0.54**	**Trigonal Bipyramidal**	7.4°	**40%**	Na(4), Ca(2), Mg(2)
**CA 13**	1.0	79.6 (76.6)	*O_2*	**0.6**	**0.78**	Octahedral	12.6°	**66%**	Na(4), Ca(3), Mg(3)
**CA 14**	1.0	108.9 (119.2)	*O_2*	**0.6**	**0.79**	Octahedral	*15.2°*	**66%**	Na(4), Ca(3), Mg(3)
**CA 15**	1.0	94.0 (98.8)		N/A	N/A	**Free**	N/A	N/A	Ca(0), Na(0), Mg(0)
**CA 16**	1.0	92.2 (80.3)	O_3	**0.6**	**0.69**	Octahedral	13.4°	**50%**	Na(5), Ca(4), Mg(4)
**CA 17**	1.0	*67.9 (86.1)*	O_4	*1.3*	**0.33**	Octahedral	*16.0°*	**33%**	Na(6), Ca(5), Mg(4)
**CA 18**	1.0	*71.7 (113.1)*	**O_2N_1**	**0.19**	**0.56**	**Trigonal Bipyramidal**	*14.3°*	**40%**	Na(1), Ca(0), Mg(0)
**CA 19**	1.0	*128.2 (83.8)*		N/A	N/A	**Free**	N/A	N/A	Ca(0), Na(0), Mg(0)
**CA 20**	1.0	**109.3 (0.0)**		N/A	N/A	**Free**	N/A	N/A	Ca(0), Na(0), Mg(0)
**CA 21**	1.0	**91.6 (0.0)**		N/A	N/A	**Free**	N/A	N/A	Ca(0), Na(0), Mg(0)
**CA 22**	1.0	*117.4 (169.4)*		N/A	N/A	**Free**	N/A	N/A	Ca(0), Na(0), Mg(0)

**CA 23**	1.0	**108.2 (0.0)**		N/A	N/A	**Free**	N/A	N/A	Ca(0), Na(0), Mg(0)
**CA 24**	1.0	*123.0 (171.1)*		N/A	N/A	**Free**	N/A	N/A	Ca(0), Na(0), Mg(0)
**CA 25**	1.0	*83.2 (106.1)*	**O_1N_1**	**0.5**	**0.86**	Octahedral	2.5°	**66%**	Ca(2), Na(3), Mg(3)
**ApoES-pre-$tRNA^{Gly}$ complex; PBD ID: 9OWJ**									
**Metal ID**	***Occupancy***	***Bfactor(env.)[1]***	***Atomic contacts***	***Valence[2]***	***nVECSUM[3]***	***Geometry[1,4]***	***gRMSD(°)[1]***	***Vacancy[1]***	**Ranking (points)**
**CA 1**	1.0	90.6 (78.3)		N/A	N/A	**Free**	N/A	N/A	Ca(0), Na(0), Mg(0)
**CA 2**	1.0	*109.3 (77.8)*		N/A	N/A	**Free**	N/A	N/A	Ca(0), Na(0), Mg(0)
**CA 3**	1.0	66.3 (68.8)	O_3	**0.9**	**0.67**	Octahedral	11.1°	**50%**	Na(5), Mg(4), Ca(4)
**CA 4**	1.0	*99.1 (79.8)*	**O_1**	**0.3**	**1.0**	**Poorly Coordinated**	N/A	N/A	Na(0), Mg(0), Ca(0)
**CA 5**	1.0	89.0 (81.4)	O_3	**0.3**	**0.67**	**Trigonal Bipyramidal**	9.9°	**40%**	Na(3), Mg(2), Ca(2)
**CA 6**	1.0	97.3 (97.3)	*O_2*	**0.6**	**0.75**	Octahedral	7.8°	**66%**	Na(4), Mg(3), Ca(3)
**CA 7**	1.0	82.4 (77.4)	O_3	**1.2**	**0.35**	Octahedral	*15.5°*	**50%**	Na(6), Mg(4), Ca(4)
**CA 8**	1.0	**105.5 (0.0)**		N/A	N/A	**Free**	N/A	N/A	Ca(0), Na(0), Mg(0)
**CA 9**	1.0	64.3 (59.5)	*O_2*	**0.4**	**0.84**	Octahedral	*14.4°*	**66%**	Na(3), Mg(3), Ca(3)
**CA 10**	1.0	108.3 (104.4)	*O_2*	**0.3**	**0.88**	Octahedral	**25.5°**	**66%**	Na(3), Mg(3), Ca(3)
**CA 11**	1.0	103.3 (99.5)	*O_2*	**0.5**	**0.56**	**Tetrahedral**	2.8°	**50%**	Na(2), Mg(1), Ca(1)
**CA 12**	1.0	96.8 (85.0)	**O_1N_1**	**0.19**	**0.93**	Octahedral	**29.3°**	**66%**	Na(3), Mg(2), Ca(2)
**CA 13**	1.0	96.1 (108.7)	**O_1N_1**	**0.3**	**0.94**	Octahedral	*19.0°*	**66%**	Na(3), Mg(3), Ca(2)
**CA 14**	1.0	93.6 (81.5)		N/A	N/A	**Free**	N/A	N/A	Ca(0), Na(0), Mg(0)
**CA 15**	1.0	71.5 (73.4)	O_4	**0.8**	**0.45**	Octahedral	12.4°	**66%**	Na(5), Mg(4), Ca(4)
**CA 16**	1.0	**109.5 (0.0)**		N/A	N/A	**Free**	N/A	N/A	Ca(0), Na(0), Mg(0)
**CA 17**	1.0	**91.9 (0.0)**		N/A	N/A	**Free**	N/A	N/A	Ca(0), Na(0), Mg(0)
**CA 18**	1.0	*121.9 (104.1)*		N/A	N/A	**Free**	N/A	N/A	Ca(0), Na(0), Mg(0)
**CA 19**	1.0	*96.5 (65.4)*		N/A	N/A	**Free**	N/A	N/A	Ca(0), Na(0), Mg(0)
**CA 20**	1.0	*101.3 (74.3)*	**O_1**	**0.06**	**1.0**	**Poorly Coordinated**	N/A	N/A	Ca(0), Na(0), Mg(0)
**CA 21**	1.0	185.8 (160.0)	*O_2*	**0.17**	**0.82**	Octahedral	*17.1°*	**66%**	Ca(3), Na(3), Mg(3)
**CA 22**	1.0	97.7 (86.4)		N/A	N/A	**Free**	N/A	N/A	Ca(0), Na(0), Mg(0)

**CA 23**	1.0	114.3 (125.3)	*O_2*	**0.6**	**0.67**	Octahedral	5.4°	**66%**	Na(4), Mg(3), Ca(3)
**CA 24**	1.0	**104.0 (0.0)**		N/A	N/A	**Free**	N/A	N/A	Ca(0), Na(0), Mg(0)
**CA 25**	1.0	94.8 (96.4)	**O_1**	**0.03**	**1.0**	**Poorly Coordinated**	N/A	N/A	Ca(0), Na(0), Mg(0)
**CA 26**	1.0	114.4 (105.7)	*O_2*	**0.6**	**0.79**	Octahedral	*14.9°*	**66%**	Na(4), Mg(3), Ca(3)
**CA 27**	1.0	*86.9 (70.5)*		N/A	N/A	**Free**	N/A	N/A	Ca(0), Na(0), Mg(0)
**CA 28**	1.0	65.7 (68.1)	O_4	**1.1**	**0.45**	**Tetrahedral**	**34.3°**	0	Na(5), Mg(2), Ca(2)
**CA 29**	1.0	**147.3 (0.0)**		N/A	N/A	**Free**	N/A	N/A	Ca(0), Na(0), Mg(0)
**CA 30**	1.0	99.3 (96.4)	**O_4N_1**	**0.8**	**0.41**	**Trigonal Bipyramidal**	*20.9°*	0	Na(2), Mg(0), Ca(0)
**CA 31**	1.0	96.3 (95.3)	*O_2*	**0.6**	**0.62**	**Tetrahedral**	3.7°	**50%**	Na(3), Mg(1), Ca(1)
**CA 32**	1.0	73.1 (63.8)	**O_1**	**0.03**	**1.0**	**Poorly Coordinated**	N/A	N/A	Ca(0), Na(0), Mg(0)
**CA 33**	1.0	*83.2 (67.9)*	O_5	*1.4*	**0.28**	Octahedral	*21.3°*	*16%*	Na(6), Ca(5), Mg(4)
**CA 34**	1.0	*97.2 (78.8)*	**O_1**	**0.3**	**1.0**	**Poorly Coordinated**	N/A	N/A	Na(0), Mg(0), Ca(0)
**ApoES-nc-pre-tRNA^{Gly} complex; PBD ID: 9OWN**									
**Metal ID**	***Occupancy***	***Bfactor(env.)***[1]	***Atomic contacts***	***Valence***[2]	***nVECSUM***[3]	***Geometry***[1,4]	***gRMSD(°)***[1]	***Vacancy***[1]	**Ranking (points)**
**CA 1**	1.0	**69.6 (0.0)**		N/A	N/A	**Free**	N/A	N/A	**Ca(0)**, Na(0), Mg(0)
**CA 2**	1.0	*73.9 (50.3)*	**O_1**	**0.05**	**1.0**	**Poorly Coordinated**	N/A	N/A	**Ca(0)**, Na(0), Mg(0)
**CA 3**	1.0	48.6 (47.2)	O_3	**0.9**	**0.63**	Octahedral	9.2°	**50%**	Na(5), Mg(4), **Ca(4)**
**CA 4**	1.0	*72.1 (54.5)*	**O_1**	**0.3**	**1.0**	**Poorly Coordinated**	N/A	N/A	Na(0), Mg(0), **Ca(0)**
**CA 5**	1.0	*72.5 (50.8)*	O_4	**0.3**	**0.66**	Octahedral	*14.6°*	**33%**	Na(4), Mg(4), **Ca(4)**
**CA 6**	1.0	62.0 (57.7)	*O_2*	**0.6**	**0.79**	Octahedral	*13.9°*	**66%**	Na(4), Mg(3), **Ca(3)**
**CA 7**	1.0	49.7 (54.5)	O_3	**1.1**	**0.36**	Octahedral	*20.2°*	**50%**	Na(6), Mg(4), **Ca(4)**
**CA 8**	1.0	**68.1 (0.0)**		N/A	N/A	**Free**	N/A	N/A	**Ca(0)**, Na(0), Mg(0)
**CA 9**	1.0	*50.1 (35.3)*	*O_2*	**0.2**	**0.83**	Octahedral	**22.0°**	**66%**	**Ca(3)**, Na(3), Mg(3)
**CA 10**	1.0	78.2 (68.1)	O_4	**0.3**	**0.64**	**Tetrahedral**	**32.4°**	0	Na(3), Mg(2), **Ca(2)**
**CA 11**	1.0	79.7 (69.1)	*O_2*	**0.4**	**0.67**	**Tetrahedral**	3.3°	**50%**	Na(2), Mg(1), **Ca(1)**
**CA 12**	1.0	*57.2 (43.5)*	O_3	**0.4**	**0.7**	Octahedral	**24.6°**	**50%**	Na(4), Mg(4), **Ca(4)**
**CA 13**	1.0	*79.8 (49.6)*	**O_1**	**0.3**	**1.0**	**Poorly Coordinated**	N/A	N/A	Na(0), Mg(0), **Ca(0)**

**CA 14**	1.0	58.8 (64.6)	**O_2N_1**	**0.19**	**0.57**	**Trigonal Bipyramidal**	*16.8°*	**40%**	Na(1), Mg(0), **Ca(0)**
**CA 15**	1.0	**71.3 (22.4)**		N/A	N/A	**Free**	N/A	N/A	**Ca(0)**, Na(0), Mg(0)
**CA 16**	1.0	48.6 (47.0)	O_4	**0.9**	**0.37**	Octahedral	*14.2°*	**66%**	Na(5), Mg(4), **Ca(4)**
**CA 17**	1.0	**76.0 (0.0)**		N/A	N/A	**Free**	N/A	N/A	**Ca(0)**, Na(0), Mg(0)
**CA 18**	1.0	**66.0 (0.0)**		N/A	N/A	**Free**	N/A	N/A	**Ca(0)**, Na(0), Mg(0)
**CA 19**	1.0	75.7 (73.3)		N/A	N/A	**Free**	N/A	N/A	**Ca(0)**, Na(0), Mg(0)
**CA 20**	1.0	*68.1 (39.7)*	**O_1**	**0.09**	**1.0**	**Poorly Coordinated**	N/A	N/A	**Ca(0)**, Na(0), Mg(0)
**CA 21**	1.0	**83.2 (42.1)**	*O_2*	**0.09**	**0.84**	Octahedral	**24.6°**	**66%**	**Ca(3)**, Na(3), Mg(3)
**CA 22**	1.0	*130.7 (153.0)*	**O_1**	**0.03**	**1.0**	**Poorly Coordinated**	N/A	N/A	**Ca(0)**, Na(0), Mg(0)
**CA 23**	1.0	79.3 (80.5)	*O_2*	**0.6**	**0.73**	Octahedral	3.2°	**66%**	Na(4), Mg(3), **Ca(3)**
**CA 24**	1.0	**82.4 (0.0)**		N/A	N/A	**Free**	N/A	N/A	**Ca(0)**, Na(0), Mg(0)
**CA 25**	1.0	**77.8 (0.0)**		N/A	N/A	**Free**	N/A	N/A	**Ca(0)**, Na(0), Mg(0)
**CA 26**	1.0	79.4 (75.1)	*O_2*	**0.4**	**0.87**	Octahedral	3.0°	**66%**	Na(3), Mg(3), **Ca(3)**
**CA 27**	1.0	80.4 (70.5)	*O_2*	**0.5**	**0.81**	Octahedral	*16.7°*	**66%**	Na(4), Mg(3), **Ca(3)**
**CA 28**	1.0	35.9 (36.3)	O_4	*1.3*	**0.4**	Octahedral	*14.0°*	**33%**	Na(6), **Ca(5)**, Mg(4)
**CA 29**	1.0	**89.4 (0.0)**		N/A	N/A	**Free**	N/A	N/A	**Ca(0)**, Na(0), Mg(0)
**CA 30**	1.0	64.9 (61.8)	*O_2*	**0.4**	**0.71**	Octahedral	7.3°	**66%**	Na(3), Mg(3), **Ca(3)**
**CA 31**	1.0	41.2 (44.6)	O_5	*1.4*	0.076	**Tetrahedral**	*15.7°*	*25%*	Na(5), **Ca(3)**, Mg(2)
**ApoES-LB-pre-tRNA^{Gly} complex; PBD ID: 9OWQ**									
**Metal ID**	***Occupancy***	***Bfactor(env.)***[1]	***Atomic contacts***	***Valence***[2]	***nVECSUM***[3]	***Geometry***[1,4]	***gRMSD(°)***[1]	***Vacancy***[1]	**Ranking (Points)**
**CA 1**	1.0	**57.6 (0.0)**		N/A	N/A	**Free**	N/A	N/A	**Ca(0)**, Na(0), Mg(0)
**CA 2**	1.0	*61.8 (42.2)*		N/A	N/A	**Free**	N/A	N/A	**Ca(0)**, Na(0), Mg(0)
**CA 3**	1.0	34.4 (33.9)	O_3	**1.0**	**0.65**	Octahedral	11.2°	**50%**	Na(6), Mg(4), **Ca(4)**
**CA 4**	1.0	*60.4 (38.4)*	**O_1**	**0.3**	**1.0**	**Poorly Coordinated**	N/A	N/A	Na(0), Mg(0), **Ca(0)**
**CA 5**	1.0	*64.8 (38.6)*	**O_1**	**0.04**	**1.0**	**Poorly Coordinated**	N/A	N/A	**Ca(0)**, Na(0), Mg(0)
**CA 6**	1.0	45.2 (42.0)	*O_2*	**0.6**	**0.77**	Octahedral	11.4°	**66%**	Na(4), Mg(3), **Ca(3)**
**CA 7**	1.0	41.9 (41.5)	O_3	*1.3*	**0.41**	Octahedral	*13.6°*	**50%**	Na(6), **Ca(5)**, Mg(4)

**CA 8**	1.0	*75.7 (44.1)*		N/A	N/A	**Free**	N/A	N/A	**Ca(0)**, Na(0), Mg(0)
**CA 9**	1.0	*43.8 (30.8)*	*O_2*	**0.4**	**0.82**	Octahedral	*17.1°*	**66%**	Na(3), Mg(3), **Ca(3)**
**CA 10**	1.0	60.3 (55.9)	O_3	**0.6**	**0.73**	Octahedral	**25.2°**	**50%**	Na(5), Mg(4), **Ca(4)**
**CA 11**	1.0	*64.0 (52.1)*	*O_2*	**0.4**	**0.66**	**Tetrahedral**	5.7°	**50%**	Na(2), Mg(1), **Ca(1)**
**CA 12**	1.0	*47.8 (34.8)*	*O_2*	**0.6**	**0.68**	Octahedral	3.7°	**66%**	Na(4), Mg(3), **Ca(3)**
**CA 13**	1.0	58.9 (66.9)	**O_2N_1**	**0.8**	**0.53**	**Trigonal Bipyramidal**	12.0°	**40%**	Na(2), Mg(0), **Ca(0)**
**CA 14**	1.0	**55.9 (28.4)**		N/A	N/A	**Free**	N/A	N/A	**Ca(0)**, Na(0), Mg(0)
**CA 15**	1.0	41.6 (41.4)	O_4	**0.8**	**0.44**	Octahedral	9.3°	**66%**	Na(5), Mg(4), **Ca(4)**
**CA 16**	1.0	*72.1 (45.2)*		N/A	N/A	**Free**	N/A	N/A	**Ca(0)**, Na(0), Mg(0)
**CA 17**	1.0	**65.4 (27.7)**		N/A	N/A	**Free**	N/A	N/A	**Ca(0)**, Na(0), Mg(0)
**CA 18**	1.0	**70.4 (0.0)**		N/A	N/A	**Free**	N/A	N/A	**Ca(0)**, Na(0), Mg(0)
**CA 19**	1.0	**61.5 (33.0)**	**O_1**	**0.12**	**1.0**	**Poorly Coordinated**	N/A	N/A	**Ca(0)**, Na(0), Mg(0)
**CA 20**	1.0	**73.1 (29.0)**	**O_1**	**0.06**	**1.0**	**Poorly Coordinated**	N/A	N/A	**Ca(0)**, Na(0), Mg(0)
**CA 21**	1.0	124.6 (143.7)	**O_1**	**0.2**	**1.0**	**Poorly Coordinated**	N/A	N/A	**Ca(0)**, Na(0), Mg(0)
**CA 22**	1.0	76.7 (66.1)	*O_2*	**0.6**	**0.75**	Octahedral	7.4°	**66%**	Na(4), Mg(3), **Ca(3)**
**CA 23**	1.0	**74.5 (0.0)**		N/A	N/A	**Free**	N/A	N/A	**Ca(0)**, Na(0), Mg(0)
**CA 24**	1.0	*84.5 (63.3)*		N/A	N/A	**Free**	N/A	N/A	**Ca(0)**, Na(0), Mg(0)
**CA 25**	1.0	*71.9 (59.1)*	*O_2*	**0.5**	**0.75**	Octahedral	6.8°	**66%**	Na(3), Mg(3), **Ca(3)**
**CA 26**	1.0	**65.1 (32.5)**	*O_2*	**0.07**	**0.76**	Octahedral	4.0°	**66%**	**Ca(3)**, Na(3), Mg(3)
**CA 27**	1.0	30.5 (27.8)	O_4	*1.4*	**0.39**	Octahedral	*14.1°*	**33%**	Na(6), **Ca(5)**, Mg(4)
**CA 28**	1.0	58.6 (51.0)	*O_2*	**0.4**	**0.79**	Octahedral	3.0°	**66%**	Na(3), Mg(3), **Ca(3)**
**CA 29**	1.0	*44.0 (31.5)*		N/A	N/A	**Free**	N/A	N/A	**Ca(0)**, Na(0), Mg(0)
**CA 30**	1.0	49.6 (44.6)	**O_3N_1**	**0.6**	*0.17*	Octahedral	**30.1°**	**33%**	Na(5), Mg(4), **Ca(2)**
**CA 31**	1.0	*48.5 (33.0)*	O_5	*1.3*	**0.27**	Octahedral	*21.5°*	*16%*	Na(6), **Ca(5)**, Mg(4)
**ApoE-mature $tRNA^{Gly}$ complex in 5 mM Ca^{2+}; PBD ID: 9OWL**									
**Metal ID**	***Occupancy***	***Bfactor(env.)***[1]	***Atomic contacts***	***Valence***[2]	***nVECSUM***[3]	***Geometry***[1,4]	***gRMSD(°)***[1]	***Vacancy***[1]	**Ranking (Points)**
**CA 1**	1.0	**81.8 (0.0)**		N/A	N/A	**Free**	N/A	N/A	**Ca(0)**, Na(0), Mg(0)

**CA 2**	1.0	*103.3 (82.9)*		N/A	N/A	**Free**	N/A	N/A	**Ca(0)**, Na(0), Mg(0)
**CA 3**	1.0	87.1 (81.7)	$\mathbf{O_1}$	**0.3**	**1.0**	**Poorly Coordinated**	N/A	N/A	Na(0), Mg(0), **Ca(0)**
**CA 4**	1.0	*129.0 (95.6)*	$\mathbf{O_1}$	**0.3**	**1.0**	**Poorly Coordinated**	N/A	N/A	Na(0), Mg(0), **Ca(0)**
**CA 5**	1.0	87.2 (80.6)		N/A	N/A	**Free**	N/A	N/A	**Ca(0)**, Na(0), Mg(0)
**CA 6**	1.0	86.1 (93.1)	O_2	**0.6**	**0.76**	Octahedral	8.6°	**66%**	Na(4), Mg(3), **Ca(3)**
**CA 7**	1.0	73.3 (73.4)	O_3	**1.2**	**0.33**	Octahedral	9.3°	**50%**	Na(6), Mg(4), **Ca(4)**
**CA 8**	1.0	**83.4 (0.0)**		N/A	N/A	**Free**	N/A	N/A	**Ca(0)**, Na(0), Mg(0)
**CA 9**	1.0	58.1 (55.9)	O_2	**0.4**	**0.81**	Octahedral	*16.9°*	**66%**	Na(3), Mg(3), **Ca(3)**
**CA 10**	1.0	91.3 (95.7)	O_3	**0.5**	**0.8**	Octahedral	**27.5°**	**50%**	Na(4), Mg(4), **Ca(4)**
**CA 11**	1.0	*126.9 (108.9)*		N/A	N/A	**Free**	N/A	N/A	**Ca(0)**, Na(0), Mg(0)
**CA 12**	1.0	95.4 (89.8)	O_2	**0.7**	**0.77**	Octahedral	10.3°	**66%**	Na(4), Mg(3), **Ca(3)**
**CA 13**	1.0	96.7 (83.5)	O_2	**0.6**	**0.72**	Octahedral	2.0°	**66%**	Na(4), Mg(3), **Ca(3)**
**CA 14**	1.0	*97.8 (75.3)*	$\mathbf{O_1}$	**0.4**	**1.0**	**Poorly Coordinated**	N/A	N/A	Na(0), Mg(0), **Ca(0)**
**CA 15**	1.0	*86.7 (108.5)*	$\mathbf{O_2N_1}$	**0.4**	**0.57**	**Trigonal Bipyramidal**	11.2°	**40%**	Na(1), Mg(0), **Ca(0)**
**CA 16**	1.0	86.6 (75.9)		N/A	N/A	**Free**	N/A	N/A	**Ca(0)**, Na(0), Mg(0)
**CA 17**	1.0	108.0 (97.9)	$\mathbf{O_1}$	**0.13**	**1.0**	**Poorly Coordinated**	N/A	N/A	**Ca(0)**, Na(0), Mg(0)
**CA 18**	1.0	88.5 (96.9)	O_3	**0.3**	**0.75**	**Tetrahedral**	**38.3°**	*25%*	Na(3), Mg(2), **Ca(2)**
**CA 19**	1.0	68.4 (71.2)	O_4	**0.8**	**0.55**	Octahedral	9.8°	**66%**	Na(5), Mg(4), **Ca(4)**
**CA 20**	1.0	*121.0 (88.6)*		N/A	N/A	**Free**	N/A	N/A	**Ca(0)**, Na(0), Mg(0)
**CA 21**	1.0	**98.9 (0.0)**		N/A	N/A	**Free**	N/A	N/A	**Ca(0)**, Na(0), Mg(0)
**CA 22**	1.0	*117.4 (96.0)*		N/A	N/A	**Free**	N/A	N/A	**Ca(0)**, Na(0), Mg(0)
**CA 23**	1.0	113.1 (109.8)		N/A	N/A	**Free**	N/A	N/A	**Ca(0)**, Na(0), Mg(0)
**CA 24**	1.0	113.9 (113.3)	O_2	**0.6**	**0.77**	Octahedral	10.1°	**66%**	Na(4), Mg(3), **Ca(3)**
**CA 25**	1.0	111.7 (103.3)	O_2	**0.5**	**0.89**	Octahedral	*21.5°*	**66%**	Na(3), Mg(3), **Ca(3)**
**CA 26**	1.0	**78.7 (0.0)**		N/A	N/A	**Free**	N/A	N/A	**Ca(0)**, Na(0), Mg(0)
**CA 27**	1.0	67.7 (69.0)	O_4	*1.6*	*0.18*	**Tetrahedral**	*16.4°*	0	Na(5), **Ca(3),** Mg(2)
**CA 28**	1.0	*100.0 (84.2)*	$\mathbf{O_1}$	**0.03**	**1.0**	**Poorly Coordinated**	N/A	N/A	**Ca(0)**, Na(0), Mg(0)

**CA 29**	1.0	116.3 (111.0)	**O_1**	**0.3**	**1.0**	**Poorly Coordinated**	N/A	N/A	Na(0), Mg(0), **Ca(0)**
**CA 30**	1.0	*111.8 (70.7)*		N/A	N/A	**Free**	N/A	N/A	**Ca(0)**, Na(0), Mg(0)
**CA 31**	1.0	*92.0 (66.4)*	**O_1N_1**	**0.09**	**0.71**	Octahedral	9.2°	**66%**	Na(2), Mg(2), **Ca(2)**
**CA 32**	1.0	*102.0 (83.2)*		N/A	N/A	**Free**	N/A	N/A	**Ca(0)**, Na(0), Mg(0)
**CA 33**	1.0	*97.9 (70.6)*		N/A	N/A	**Free**	N/A	N/A	**Ca(0)**, Na(0), Mg(0)
**CA 34**	1.0	*91.7 (59.9)*		N/A	N/A	**Free**	N/A	N/A	**Ca(0)**, Na(0), Mg(0)
**CA 35**	1.0	58.2 (64.1)	O_4	*1.4*	**0.31**	Octahedral	9.1°	**33%**	Na(6), **Ca(5)**, Mg(4)
**CA 36**	1.0	*94.2 (75.3)*	**O_1**	**0.3**	**1.0**	**Poorly Coordinated**	N/A	N/A	Na(0), Mg(0), **Ca(0)**
**CA 37**	1.0	**108.2 (0.0)**		N/A	N/A	**Free**	N/A	N/A	**Ca(0)**, Na(0), Mg(0)
**ApoE-mature tRNA^{Gly} complex in 10 mM Ca^{2+}; PBD ID: 9OWM**									
**Metal ID**	***Occupancy***	***Bfactor(env.)***[1]	***Atomic contacts***	***Valence***[2]	***nVECSUM***[3]	***Geometry***[1,4]	***gRMSD(°)***[1]	***Vacancy***[1]	**Ranking (Points)**
**CA 1**	1.0	80.2 (75.6)		N/A	N/A	**Free**	N/A	N/A	**Ca(0)**, Na(0), Mg(0)
**CA 2**	1.0	*91.4 (69.2)*	**O_1**	**0.03**	**1.0**	**Poorly Coordinated**	N/A	N/A	**Ca(0)**, Na(0), Mg(0)
**CA 3**	1.0	*92.9 (75.8)*	**O_1**	**0.3**	**1.0**	**Poorly Coordinated**	N/A	N/A	Na(0), Mg(0), **Ca(0)**
**CA 4**	1.0	*105.6 (89.9)*	**O_1**	**0.2**	**1.0**	**Poorly Coordinated**	N/A	N/A	Na(0), Mg(0), **Ca(0)**
**CA 5**	1.0	88.4 (82.6)	O_3	**0.3**	**0.69**	Octahedral	12.2°	**50%**	Na(4), Mg(4), **Ca(4)**
**CA 6**	1.0	85.1 (91.7)	*O_2*	**0.7**	**0.71**	Octahedral	0.4°	**66%**	Na(4), Mg(3), **Ca(3)**
**CA 7**	1.0	*106.0 (86.8)*	**N_1**	**0.4**	**1.0**	**Poorly Coordinated**	N/A	N/A	Na(0), Mg(0), **Ca(0)**
**CA 8**	1.0	66.5 (68.4)	O_3	**1.2**	**0.37**	Octahedral	12.1°	**50%**	Na(6), Mg(4), **Ca(4)**
**CA 9**	1.0	**94.3 (0.0)**		N/A	N/A	**Free**	N/A	N/A	**Ca(0)**, Na(0), Mg(0)
**CA 10**	1.0	*64.9 (55.1)*	*O_2*	**0.4**	**0.86**	Octahedral	*17.5°*	**66%**	Na(3), Mg(3), **Ca(3)**
**CA 11**	1.0	95.4 (88.6)	*O_2*	**0.6**	**0.8**	Octahedral	*15.7°*	**66%**	Na(4), Mg(3), **Ca(3)**
**CA 12**	1.0	*126.3 (98.8)*		N/A	N/A	**Free**	N/A	N/A	**Ca(0)**, Na(0), Mg(0)
**CA 13**	1.0	89.6 (86.6)	*O_2*	**0.5**	**0.85**	Octahedral	*16.5°*	**66%**	Na(3), Mg(3), **Ca(3)**
**CA 14**	1.0	85.4 (73.8)	O_4	**0.4**	**0.72**	**Tetrahedral**	**34.2°**	0	Na(3), Mg(2), **Ca(2)**
**CA 15**	1.0	*77.7 (93.5)*	**O_2N_1**	**0.4**	**0.75**	**Trigonal Bipyramidal**	9.6°	**40%**	Na(1), Mg(0), **Ca(0)**
**CA 16**	1.0	132.7 (124.5)	*O_2*	**0.1**	**0.91**	Octahedral	**40.4°**	**66%**	**Ca(3)**, Na(3), Mg(3)

**CA 17**	1.0	*90.8 (65.5)*		N/A	N/A	**Free**	N/A	N/A	**Ca(0)**, Na(0), Mg(0)
**CA 18**	1.0	151.2 (144.4)	O_3	**0.6**	**0.66**	**Trigonal Bipyramidal**	*17.6°*	**40%**	Na(4), Mg(2), **Ca(2)**
**CA 19**	1.0	98.4 (90.0)	**O_1**	**0.13**	**1.0**	**Poorly Coordinated**	N/A	N/A	**Ca(0)**, Na(0), Mg(0)
**CA 20**	1.0	77.7 (83.6)	*O_2*	**0.19**	**0.69**	Octahedral	6.4°	**66%**	**Ca(3)**, Na(3), Mg(3)
**CA 21**	1.0	71.6 (64.1)	O_3	**0.6**	**0.62**	Octahedral	*19.6°*	**50%**	Na(5), Mg(4), **Ca(4)**
**CA 22**	1.0	*94.9 (71.4)*		N/A	N/A	**Free**	N/A	N/A	**Ca(0)**, Na(0), Mg(0)
**CA 23**	1.0	**83.6 (0.0)**		N/A	N/A	**Free**	N/A	N/A	**Ca(0)**, Na(0), Mg(0)
**CA 24**	1.0	*106.8 (86.9)*		N/A	N/A	**Free**	N/A	N/A	**Ca(0)**, Na(0), Mg(0)
**CA 25**	1.0	**109.9 (0.0)**		N/A	N/A	**Free**	N/A	N/A	**Ca(0)**, Na(0), Mg(0)
**CA 26**	1.0	101.5 (104.0)	*O_2*	**0.5**	**0.69**	Octahedral	2.8°	**66%**	Na(3), Mg(3), **Ca(3)**
**CA 27**	1.0	*105.3 (89.7)*	*O_2*	**0.6**	**0.8**	Octahedral	*16.2°*	**66%**	Na(4), Mg(3), **Ca(3)**
**CA 28**	1.0	90.0 (84.5)	**O_3N_1**	**0.6**	**0.44**	**Tetrahedral**	**34.6°**	0	Na(2), Mg(0), **Ca(0)**
**CA 29**	1.0	*95.3 (81.7)*		N/A	N/A	**Free**	N/A	N/A	**Ca(0)**, Na(0), Mg(0)
**CA 30**	1.0	132.9 (122.6)	O_3	**0.8**	**0.32**	Octahedral	**28.9°**	**50%**	Na(5), Mg(4), **Ca(4)**
**CA 31**	1.0	70.8 (63.7)	O_5	*1.6*	*0.17*	Octahedral	12.7°	**50%**	Na(6), **Ca(5)**, Mg(4)
**ApoE (consensus) in 5 mM Mg^{2+}; PBD ID: 9OVB**									
**Metal ID**	***Occupancy***	***Bfactor(env.)[1]***	***Atomic contacts***	***Valence[2]***	***nVECSUM[3]***	***Geometry[1,4]***	***gRMSD(°)[1]***	***Vacancy[1]***	**Ranking (Points)**
**MG 1**	1.0	**91.4 (0.0)**		N/A	N/A	**Free**	N/A	N/A	**Mg(0)**, Na(0)
**MG 2**	1.0	*100.7 (127.4)*		N/A	N/A	**Free**	N/A	N/A	**Mg(0)**, Na(0)
**MG 3**	1.0	**36.0 (0.0)**		N/A	N/A	**Free**	N/A	N/A	**Mg(0)**, Na(0)
**MG 4**	1.0	56.7 (55.0)	**O_1**	**0.12**	**1.0**	**Poorly Coordinated**	N/A	N/A	Na(0), **Mg(0)**
**MG 5**	1.0	**35.9 (78.9)**		N/A	N/A	**Free**	N/A	N/A	**Mg(0)**, Na(0)
**MG 6**	1.0	*53.3 (66.6)*	**N_1**	**0.4**	**1.0**	**Poorly Coordinated**	N/A	N/A	Na(1), **Mg(0)**
**MG 7**	1.0	127.6 (136.4)	O_3	**0.5**	**0.88**	Octahedral	*18.7°*	**50%**	Na(5), **Mg(4)**
**MG 8**	1.0	*50.3 (71.0)*	**N_1**	**0.14**	**1.0**	**Poorly Coordinated**	N/A	N/A	Na(0), **Mg(0)**
**MG 9**	1.0	57.8 (64.7)	*O_2*	**0.6**	**0.76**	Octahedral	3.4°	**66%**	Na(5), **Mg(3)**
**MG 10**	1.0	*62.7 (74.6)*	**O_1**	**0.4**	**1.0**	**Poorly Coordinated**	N/A	N/A	Na(1), **Mg(0)**

**MG 11**	1.0	79.8 (82.9)	O_4	**0.8**	**0.37**	Octahedral	7.6°	**66%**	Na(6), **Mg(4)**
**MG 12**	1.0	*49.5 (65.8)*	O_4	**0.8**	**0.28**	**Tetrahedral**	**25.3°**	0	Na(5), **Mg(2)**
**MG 13**	1.0	**59.5 (0.0)**		N/A	N/A	**Free**	N/A	N/A	**Mg(0)**, Na(0)
**MG 14**	1.0	55.5 (61.2)	*O_2*	**0.8**	**0.62**	**Tetrahedral**	6.6°	**50%**	Na(4), **Mg(1)**
**MG 15**	1.0	70.9 (72.4)	O_4	*1.3*	**0.32**	Octahedral	*16.8°*	**33%**	Mn(6), **Mg(5)**
**MG 16**	1.0	**95.4 (0.0)**		N/A	N/A	**Free**	N/A	N/A	**Mg(0)**, Na(0)
**MG 17**	1.0	*118.0 (93.6)*	*O_1N_1*	**0.2**	**0.8**	Octahedral	12.6°	**66%**	Na(3), **Mg(3)**
**MG 18**	1.0	**106.0 (0.0)**		N/A	N/A	**Free**	N/A	N/A	**Mg(0)**, Na(0)
**MG 19**	1.0	**78.9 (0.0)**		N/A	N/A	**Free**	N/A	N/A	**Mg(0)**, Na(0)
**MG 20**	1.0	*53.9 (66.9)*		N/A	N/A	**Free**	N/A	N/A	**Mg(0)**, Na(0)
**MG 21**	1.0	*123.0 (173.5)*		N/A	N/A	**Free**	N/A	N/A	**Mg(0)**, Na(0)
**MG 22**	1.0	*106.3 (135.3)*		N/A	N/A	**Free**	N/A	N/A	**Mg(0)**, Na(0)
**MG 23**	1.0	*84.6 (133.5)*		N/A	N/A	**Free**	N/A	N/A	**Mg(0)**, Na(0)
**MG 24**	1.0	**56.1 (0.0)**		N/A	N/A	**Free**	N/A	N/A	**Mg(0)**, Na(0)
**MG 25**	1.0	135.0 (134.8)	**O_1**	**0.03**	**1.0**	**Poorly Coordinated**	N/A	N/A	**Mg(0)**, Na(0)
**MG 26**	1.0	**86.2 (0.0)**		N/A	N/A	**Free**	N/A	N/A	**Mg(0)**, Na(0)
**MG 27**	1.0	139.1 (123.4)	*O_2*	**0.3**	**0.76**	**Tetrahedral**	5.7°	**50%**	Na(3), **Mg(1)**
**ApoE (consensus) in 10 mM Mg^{2+}; PBD ID: 9OVC**									
**Metal ID**	***Occupancy***	***Bfactor(env.)***[1]	***Atomic contacts***	***Valence***[2]	***nVECSUM***[3]	***Geometry***[1,4]	***gRMSD(°)***[1]	***Vacancy***[1]	**Ranking (Points)**
**MG 1**	1.0	87.0 (89.7)		N/A	N/A	**Free**	N/A	N/A	**Mg(0)**, Na(0)
**MG 2**	1.0	*78.1 (92.0)*		N/A	N/A	**Free**	N/A	N/A	**Mg(0)**, Na(0)
**MG 3**	1.0	*55.6 (66.2)*		N/A	N/A	**Free**	N/A	N/A	**Mg(0)**, Na(0)
**MG 4**	1.0	86.7 (86.6)	**O_1**	**0.03**	**1.0**	**Poorly Coordinated**	N/A	N/A	**Mg(0)**, Na(0)
**MG 5**	1.0	*63.1 (79.0)*		N/A	N/A	**Free**	N/A	N/A	**Mg(0)**, Na(0)
**MG 6**	1.0	*65.0 (48.4)*	**N_1**	**0.4**	**1.0**	**Poorly Coordinated**	N/A	N/A	Na(1), **Mg(0)**
**MG 7**	1.0	128.0 (145.7)	**O_1**	**0.4**	**1.0**	**Poorly Coordinated**	N/A	N/A	Na(1), **Mg(0)**
**MG 8**	1.0	93.4 (100.0)	**N_1**	**0.02**	**1.0**	**Poorly Coordinated**	N/A	N/A	**Mg(0)**, Na(0)

**MG 9**	1.0	65.4 (69.6)	*O_2*	**0.7**	**0.7**	Octahedral	2.1°	**66%**	Na(5), **Mg(3)**
**MG 10**	1.0	72.2 (63.4)	**O_1**	**0.4**	**1.0**	**Poorly Coordinated**	N/A	N/A	Na(1), **Mg(0)**
**MG 11**	1.0	54.8 (59.4)	O_3	**0.6**	**0.63**	**Tetrahedral**	*16.5°*	*25%*	Na(5), **Mg(2)**
**MG 12**	1.0	72.2 (71.3)	*O_2*	**0.4**	**0.56**	**Linear**	*19.1°*	0	Na(2), **Mg(1)**
**MG 13**	1.0	**69.3 (0.0)**		N/A	N/A	**Free**	N/A	N/A	**Mg(0)**, Na(0)
**MG 14**	1.0	*53.1 (63.7)*	*O_2*	**0.5**	**0.79**	Octahedral	4.5°	**66%**	Na(5), **Mg(3)**
**MG 15**	1.0	*50.6 (59.3)*	O_4	**1.1**	**0.39**	Octahedral	11.6°	**33%**	Na(5), **Mg(4)**
**MG 16**	1.0	**96.0 (0.0)**		N/A	N/A	**Free**	N/A	N/A	**Mg(0)**, Na(0)
**MG 17**	1.0	*94.1 (116.9)*	**N_1**	**0.04**	**1.0**	**Poorly Coordinated**	N/A	N/A	**Mg(0)**, Na(0)
**MG 18**	1.0	*69.6 (56.9)*		N/A	N/A	**Free**	N/A	N/A	**Mg(0)**, Na(0)
**MG 19**	1.0	**55.9 (0.0)**		N/A	N/A	**Free**	N/A	N/A	**Mg(0)**, Na(0)
**MG 20**	1.0	80.4 (72.2)	*O_2*	**0.6**	**0.72**	Octahedral	0.7°	**66%**	Na(5), **Mg(3)**
**MG 21**	1.0	81.9 (70.8)		N/A	N/A	**Free**	N/A	N/A	**Mg(0)**, Na(0)
**MG 22**	1.0	*73.1 (50.8)*	**O_1**	**0.03**	**1.0**	**Poorly Coordinated**	N/A	N/A	**Mg(0)**, Na(0)
**MG 23**	1.0	82.1 (76.9)	**O_1**	**0.4**	**1.0**	**Poorly Coordinated**	N/A	N/A	Na(1), **Mg(0)**
**MG 24**	1.0	69.9 (67.2)	**N_1**	**0.03**	**1.0**	**Poorly Coordinated**	N/A	N/A	**Mg(0)**, Na(0)
**MG 25**	1.0	*85.1 (57.0)*		N/A	N/A	**Free**	N/A	N/A	**Mg(0)**, Na(0)
**MG 26**	1.0	*80.3 (62.6)*	**O_1**	**0.1**	**1.0**	**Poorly Coordinated**	N/A	N/A	**Mg(0)**, Na(0)
**MG 27**	1.0	**97.8 (0.0)**		N/A	N/A	**Free**	N/A	N/A	**Mg(0)**, Na(0)
**MG 28**	1.0	*111.8 (135.1)*	**O_1**	**0.04**	**1.0**	**Poorly Coordinated**	N/A	N/A	**Mg(0)**, Na(0)
**MG 29**	1.0	107.3 (114.7)	*O_1N_1*	**0.5**	**0.95**	Octahedral	*17.8°*	**66%**	Na(4), **Mg(3)**
**MG 30**	1.0	**118.5 (0.0)**		N/A	N/A	**Free**	N/A	N/A	**Mg(0)**, Na(0)
**MG 31**	1.0	**104.5 (0.0)**		N/A	N/A	**Free**	N/A	N/A	**Mg(0)**, Na(0)
**MG 32**	1.0	*105.8 (142.6)*	**O_1**	**0.3**	**1.0**	**Poorly Coordinated**	N/A	N/A	Na(1), **Mg(0)**
**MG 33**	1.0	**100.5 (0.0)**		N/A	N/A	**Free**	N/A	N/A	**Mg(0)**, Na(0)
**MG 34**	1.0	96.0 (86.2)	**O_1**	**0.18**	**1.0**	**Poorly Coordinated**	N/A	N/A	Na(0), **Mg(0)**
**MG 35**	1.0	*51.8 (68.1)*	*O_1N_1*	**0.7**	**0.69**	Octahedral	5.7°	**66%**	Na(5), **Mg(3)**

**MG 36**	1.0	*104.0 (82.8)*		N/A	N/A	**Free**	N/A	N/A	**Mg(0)**, Na(0)
**MG 37**	1.0	**110.6 (0.0)**		N/A	N/A	**Free**	N/A	N/A	**Mg(0)**, Na(0)
**MG 38**	1.0	124.2 (132.4)		N/A	N/A	**Free**	N/A	N/A	**Mg(0)**, Na(0)
**MG 39**	1.0	*94.7 (79.4)*		N/A	N/A	**Free**	N/A	N/A	**Mg(0)**, Na(0)
**MG 40**	1.0	**94.3 (0.0)**		N/A	N/A	**Free**	N/A	N/A	**Mg(0)**, Na(0)
**MG 41**	1.0	103.9 (101.0)		N/A	N/A	**Free**	N/A	N/A	**Mg(0)**, Na(0)
**MG 42**	1.0	114.6 (106.4)		N/A	N/A	**Free**	N/A	N/A	**Mg(0)**, Na(0)
**MG 43**	1.0	93.4 (89.4)		N/A	N/A	**Free**	N/A	N/A	**Mg(0)**, Na(0)
**MG 44**	1.0	115.1 (115.6)		N/A	N/A	**Free**	N/A	N/A	**Mg(0)**, Na(0)

Supplementary references:

1 Punjani, A., Rubinstein, J. L., Fleet, D. J. & Brubaker, M. A. cryoSPARC: algorithms for rapid unsupervised cryo-EM structure determination. Nat Methods 14, 290–296 (2017). https://doi.org/10.1038/nmeth.4169

2 Wu, J. et al. Cryo-EM Structure of the Human Ribonuclease P Holoenzyme. Cell 175, 1393–1404 e1311 (2018). https://doi.org/10.1016/j.cell.2018.10.003

3 Wan, F. et al. Cryo-electron microscopy structure of an archaeal ribonuclease P holoenzyme. Nat Commun 10, 2617 (2019). https://doi.org/10.1038/s41467-019-10496-3

4 Reiter, N. J. et al. Structure of a bacterial ribonuclease P holoenzyme in complex with tRNA. Nature 468, 784–789 (2010). https://doi.org/10.1038/nature09516

5 Nowotny, M., Gaidamakov, S. A., Crouch, R. J. & Yang, W. Crystal structures of RNase H bound to an RNA/DNA hybrid: Substrate specificity and metal-dependent catalysis. Cell 121, 1005–1016 (2005). https://doi.org/10.1016/j.cell.2005.04.024

6 Teramoto, T. et al. Structural basis of transfer RNA processing by bacterial minimal RNase P. Nat Commun 16, 5456 (2025). https://doi.org/10.1038/s41467-025-60002-1

7 Zheng, H. et al. Validation of metal-binding sites in macromolecular structures with the CheckMyMetal web server. Nat Protoc 9, 156–170 (2014). https://doi.org/10.1038/nprot.2013.172
