# Supplementary material for: Structural basis for protein-free catalysis by ribonuclease P ribozyme
Source: Nat Commun. 2026 Apr 15;17:5209. doi: 10.1038/s41467-026-71597-4 (PMC13254310; doi:10.1038/s41467-026-71597-4)
Supplement: Supplementary file 2 — Reporting Summary [file 41467_2026_71597_MOESM2_ESM.pdf]

nature portfolio

Corresponding author(s): Jinwei Zhang, Jason R. Stagno, Yun-Xing Wang

Last updated by author(s): Mar 17, 2026

Reporting Summary

Nature Portfolio wishes to improve the reproducibility of the work that we publish. This form provides structure for consistency and transparency in reporting. For further information on Nature Portfolio policies, see our Editorial Policies and the Editorial Policy Checklist.

Statistics

For all statistical analyses, confirm that the following items are present in the figure legend, table legend, main text, or Methods section.

n/a	Confirmed	
☐	☒	The exact sample size (*n*) for each experimental group/condition, given as a discrete number and unit of measurement
☐	☒	A statement on whether measurements were taken from distinct samples or whether the same sample was measured repeatedly
☒	☐	The statistical test(s) used AND whether they are one- or two-sided<br>*Only common tests should be described solely by name; describe more complex techniques in the Methods section.*
☒	☐	A description of all covariates tested
☒	☐	A description of any assumptions or corrections, such as tests of normality and adjustment for multiple comparisons
☐	☒	A full description of the statistical parameters including central tendency (e.g. means) or other basic estimates (e.g. regression coefficient) AND variation (e.g. standard deviation) or associated estimates of uncertainty (e.g. confidence intervals)
☒	☐	For null hypothesis testing, the test statistic (e.g. *F*, *t*, *r*) with confidence intervals, effect sizes, degrees of freedom and *P* value noted<br>*Give P values as exact values whenever suitable.*
☒	☐	For Bayesian analysis, information on the choice of priors and Markov chain Monte Carlo settings
☒	☐	For hierarchical and complex designs, identification of the appropriate level for tests and full reporting of outcomes
☒	☐	Estimates of effect sizes (e.g. Cohen's *d*, Pearson's *r*), indicating how they were calculated

Our web collection on statistics for biologists contains articles on many of the points above.

Software and code

Policy information about availability of computer code

Data collection	Thermo Scientific Smart EPU Software (2.9) for cryo-EM data collection;<br>Mass Hunter Qualitative Analysis software (v.B.07.00) with Bioconfirm Workflow for mass spectrometry data collection;<br>AcquireMP (version 2023 R1.1) software for mass photometry data acquisition.
Data analysis	All software data analysis is publicly accessible.<br>Cryo-EM data processing: CryoSPARC (v.4.7.1);<br>Structural visualization: ChimeraX (1.9);<br>Metal ion validation: CheckMyMetal online program: https://cmm.minorlab.org/;<br>DiscoverMP (version 2023 R1.2) software for mass photometry data analysis and figure making;<br>Initial RNA model building: ModeRNA (2.6), PyMol (open-source v.1.20.);<br>Model refinement and validation: Coot (0.9.8.96), Phenix (1.21.2), SimRNA (3.2), ISOLDE (1.10.1);<br>RNA secondary structure drawing: Adobe Illustrator 2025.<br>Statistical figure drawing and plotting: GraphPad Prism (Version 10.6.1), Bio-Rad Image Lab 6.1 software, ImageJ (2.14.0).<br>Nonlinear regression fit: GraphPad Prism (Version 10.6.1).

For manuscripts utilizing custom algorithms or software that are central to the research but not yet described in published literature, software must be made available to editors and reviewers. We strongly encourage code deposition in a community repository (e.g. GitHub). See the Nature Portfolio guidelines for submitting code & software for further information.

Data

Policy information about availability of data

All manuscripts must include a data availability statement. This statement should provide the following information, where applicable:

- Accession codes, unique identifiers, or web links for publicly available datasets
- A description of any restrictions on data availability
- For clinical datasets or third party data, please ensure that the statement adheres to our policy

Cryo-EM reconstructions and structural models for RNase P RNA-alone (apoE), RNA-substrate complex (apoES), and RNA-product complex (apoEP), generated in this study, have been deposited in the Electron Microscopy Data Bank (EMDB) at https://www.ebi.ac.uk/emdb/ and Protein Data Bank (PDB) at http://www.pdb.org, respectively. Accession numbers for EMDB are:

EMD-70888 [https://www.ebi.ac.uk/emdb/EMD-70888] (apoE consensus, 5 mM Ca2+),
EMD-70891 [https://www.ebi.ac.uk/emdb/EMD-70891] (apoE class 0, 5 mM Ca2+),
EMD-70892 [https://www.ebi.ac.uk/emdb/EMD-70892] (apoE class 1, 5 mM Ca2+),
EMD-70893 [https://www.ebi.ac.uk/emdb/EMD-70893] (apoE class 2, 5 mM Ca2+),
EMD-70896 [https://www.ebi.ac.uk/emdb/EMD-70896] (apoE consensus 5 mM Mg2+),
EMD-70897 [https://www.ebi.ac.uk/emdb/EMD-70897] (apoE consensus 10 mM Mg2+),
EMD-70933 [https://www.ebi.ac.uk/emdb/EMD-70933] (apoES pre-tRNA),
EMD-70935 [https://www.ebi.ac.uk/emdb/EMD-70935] (apoEP mat-tRNA 5 mM Ca2+),
EMD-70936 [https://www.ebi.ac.uk/emdb/EMD-70936] (apoEP mat-tRNA 10 mM Ca2+),
EMD-70937 [https://www.ebi.ac.uk/emdb/EMD-70937] (apoES nc-pre-tRNA),
EMD-70940 [https://www.ebi.ac.uk/emdb/EMD-70940] (apoES LB-pre-tRNA),
EMD-70994 [https://www.ebi.ac.uk/emdb/EMD-70994] (TLm apoE class 0),
EMD-70995 [https://www.ebi.ac.uk/emdb/EMD-70995] (TLm apoE class 1),
EMD-70996 [https://www.ebi.ac.uk/emdb/EMD-70996] (TLm apoE class 2).

Corresponding accession codes for PDB are:
9OV3 [https://www.rcsb.org/structure/unreleased/9OV3] (apoE consensus 5 mM Ca2+),
9OV6 [https://www.rcsb.org/structure/unreleased/9OV6] (apoE class 0, 5 mM Ca2+),
9OV7 [https://www.rcsb.org/structure/unreleased/9OV7] (apoE class 1, 5 mM Ca2+),
9OV8 [https://www.rcsb.org/structure/unreleased/9OV8] (apoE class 2, 5 mM Ca2+),
9OVB [https://www.rcsb.org/structure/unreleased/9OVB] (apoE consensus 5 mM Mg2+),
9OVC [https://www.rcsb.org/structure/unreleased/9OVC] (apoE consensus 10 mM Mg2+),
9OWJ [https://www.rcsb.org/structure/unreleased/9OWJ] (apoES pre-tRNA),
9OWL [https://www.rcsb.org/structure/unreleased/9OWL] (apoEP mat-tRNA 5 mM Ca2+),
9OWM [https://www.rcsb.org/structure/unreleased/9OWM] (apoEP mat-tRNA 10 mM Ca2+),
9OWN [https://www.rcsb.org/structure/unreleased/9OWN] (apoES nc-pre-tRNA),
9OWQ [https://www.rcsb.org/structure/unreleased/9OWQ] (apoES LB-pre-tRNA),
9OY2 [https://www.rcsb.org/structure/unreleased/9OY2] (TLm apoE class 0),
9OY3 [https://www.rcsb.org/structure/unreleased/9OY3] (TLm apoE class 1),
9OY4 [https://www.rcsb.org/structure/unreleased/9OY4] (TLm apoE class 2).

Accession codes of PDBs for the initial structural building of RNase P RNA and tRNA:
2A64 [https://www.rcsb.org/structure/2A64] (Ribonuclease P RNA from Bacillus stearothermophilus),
4GMN [https://www.rcsb.org/structure/4MGN] (G. kaustophilus glyQS T box riboswitch Stem I in complex with tRNA),
1NBS [https://www.rcsb.org/structure/1NBS] (Specificity domain of Bacillus subtilis ribonuclease P RNA).

Research involving human participants, their data, or biological material

Policy information about studies with human participants or human data. See also policy information about sex, gender (identity/presentation), and sexual orientation and race, ethnicity and racism.

Reporting on sex and gender	N/A
Reporting on race, ethnicity, or other socially relevant groupings	N/A
Population characteristics	N/A
Recruitment	N/A
Ethics oversight	N/A

Note that full information on the approval of the study protocol must also be provided in the manuscript.

Field-specific reporting

Please select the one below that is the best fit for your research. If you are not sure, read the appropriate sections before making your selection.

☒ Life sciences ☐ Behavioural & social sciences ☐ Ecological, evolutionary & environmental sciences

For a reference copy of the document with all sections, see nature.com/documents/nr-reporting-summary-flat.pdf

Life sciences study design

All studies must disclose on these points even when the disclosure is negative.

Sample size	Sample sizes were not predetermined. The number of particles used for cryo-EM 3D reconstruction was decided by acquisition time that are sufficient for target structural resolution of 2.5-3.0 angstrom. The number of metal ions analyzed were limited by the structural resolution of the cryo-EM maps.
Data exclusions	In cryo-EM data processing, particles were excluded during 2D classification and 3D reconstruction as a standard metrics in the feild.
Replication	Enzymatic assays for RNase P wild type and tetraloop mutant are duplicated and repeated after 24 hours.<br>In cryo-EM structural reconstruction, the full data set was divided by two halves. The two halves of the data were used independently to reconstruct the cryo-EM map, ensuring the map was reproducible up to 2.5-3.0 angstrom.
Randomization	This is irrelevant to this study as there were no groupings.
Blinding	Blinding was irrelevant for this study. Structures of RNase P RNA and its substrate and product were known for all researchers who are involved in solving the structures.

Reporting for specific materials, systems and methods

We require information from authors about some types of materials, experimental systems and methods used in many studies. Here, indicate whether each material, system or method listed is relevant to your study. If you are not sure if a list item applies to your research, read the appropriate section before selecting a response.

Materials & experimental systems

n/a	Involved in the study	
☒	☐	Antibodies
☒	☐	Eukaryotic cell lines
☒	☐	Palaeontology and archaeology
☒	☐	Animals and other organisms
☒	☐	Clinical data
☒	☐	Dual use research of concern
☒	☐	Plants

Methods

n/a	Involved in the study	
☒	☐	ChIP-seq
☒	☐	Flow cytometry
☒	☐	MRI-based neuroimaging

Plants

Seed stocks	*Report on the source of all seed stocks or other plant material used. If applicable, state the seed stock centre and catalogue number. If plant specimens were collected from the field, describe the collection location, date and sampling procedures.*
Novel plant genotypes	*Describe the methods by which all novel plant genotypes were produced. This includes those generated by transgenic approaches, gene editing, chemical/radiation-based mutagenesis and hybridization. For transgenic lines, describe the transformation method, the number of independent lines analyzed and the generation upon which experiments were performed. For gene-edited lines, describe the editor used, the endogenous sequence targeted for editing, the targeting guide RNA sequence (if applicable) and how the editor was applied.*
Authentication	*Describe any authentication procedures for each seed stock used or novel genotype generated. Describe any experiments used to assess the effect of a mutation and, where applicable, how potential secondary effects (e.g. second site T-DNA insertions, mosiacism, off-target gene editing) were examined.*
