# Supplementary material for: Structural basis for protein-free catalysis by ribonuclease P ribozyme
Source: Nat Commun. 2026 Apr 15;17:5209. doi: 10.1038/s41467-026-71597-4 (PMC13254310; doi:10.1038/s41467-026-71597-4)
Supplement: Supplementary file 3 — Transparent Peer Review file [file 41467_2026_71597_MOESM3_ESM.pdf]

nature portfolio

Peer Review File

Structural basis for protein-free catalysis by ribonuclease P ribozyme

Corresponding Author: Dr Yun-Xing Wang

This file contains all reviewer reports in order by version, followed by all author rebuttals in order by version.

Version 0:

Reviewer comments:

Reviewer #1

(Remarks to the Author)
While the crystal structure of the bacterial RNase P has been determined about two decades ago, the advent of cryo-EM has opened new opportunities to shed light on its details and to provide novel insight into its function. The authors of this manuscript have capitalized on this opportunity and solved the structure of a B-type bacterial RNase P RNA in several states of the catalytic cycle. While the RNase P holoenzyme is a ribonucleoprotein complex and the molecule studied in the manuscript lacks the protein component, the results of this study are still relevant and interesting, given that the RNA component alone is capable of catalysis under special conditions.

The structures presented in the manuscript are important and reveal novel details of the B-type bacterial RNase P RNA structure and function. Of special note is the observation of the conformational changes occurring as catalysis progresses: this is a very important contribution to the field that alone makes the manuscript worthy of publication.

However, this reviewer has several concerns (below) that must be addressed before this work is, in the view of this reviewer, suitable for publication. The major concern is the rather reckless (or, at the very least, poorly justified in the text) assignment of ion identities to all potentially suitable peaks in the maps. The authors should either strongly justify these assignments in each and every case, or scale down the related claims (see Auffinger et al. RNA 2021); the results are interesting even without the incorporation of potentially less-than-rigorous ion assignments.

Comments:

Ln 27-28. Consider re-wording for clarity.

Ln 48-49. Evolutionary relevance of the activity is not unknown.

Ln 53. Weak activity has been reported for RNA alone: Kikovska et al. PNAS 2007. Role of individual proteins are described in Perederina et al. NAR 2018. It would be appropriate to cite both.

Ln 88. While technically this is a correct statement, it appears to be somewhat misleading. It must be mentioned that structures of the S-domain in isolation, as well as S-domain as a part of RNP have been reported, and the corresponding references must be provided.

Ln 148-149. The structural basis of the S-domain's role has been discussed in Krasilnikov et al. Science 2004.

Ln 151. Ext. Data Fig. 5: References must be provided.

Ln 152-153. The role of the TL/TLR, including its role in the stabilization of the IDTM region was discussed in Krasilnikov et al. Nature 2003 and Science 2004; this must be acknowledged.

Ln 168. A- and B-types RNases P differ in their structural arrangements. The broader implications of the suggested "allosteric switch" role of TL/TLR should be discussed taking into account these differences. Alternatively, it should be clearly stated that the findings discussed in the manuscript are limited to a specific type of RNase P, without unwarranted

generalizations.

Ln 181 and on. A major concern. Assigning Mg2+/Ca2+ to all 10+ sigma picks (and there are dozens of those) without proper justification is reckless (see Auffinger et al. RNA 2021). Proper and thorough justification should be given to the differentiation between Mg2+/Ca2+ and water/other metal ions (Na+, specifically); when it's not possible, this should be clearly stated. As is, these assignments are putative, at best.

Ln 271-272. This claim requires a rigorous proof of the ion's identity, which is not provided.
Ln 668 and below. It would be appropriate to acknowledge actual publications, not just PDB IDs.

Table 1. Geometry parameters, including geometry outliers, must be included in the table.

Provided maps and PDB files:

1. Refinement appears to be sloppy in many instances and has to be re-done; this would be evident if geometry parameters of the models were provided, but even the provided excessively high clash scores are telling.

2. There is evidence (such as B-factors set to 20.00) of post-refinement ("by hand") modifications of the models (mutations of residues?).

3. In the supplementary information, plots showing per-residue correlation coefficients for all models must be presented. This is especially important given the low local resolution in the peripheral parts of the molecules.

Reviewer #2

(Remarks to the Author)
This paper focuses on an important and timely biological topic: the mechanism of catalysis and substrate recognition by bacterial RNase P. This conserved ribonucleoprotein uses an RNA core to catalyze tRNA 5' end processing. The authors present several cryo-EM structures of the RNA core of RNase P in its "apo" form (i.e., without its protein components), WT/mutant and in complex with substrate, product, or various concentrations of Mg^{2+}/Ca^{2+}. A limited amount of functional data is also provided. The novel insights arising from these structures are suggested to include the first visualization of the S-domain, identification of several divalent metal ions that stabilize the structure, and molecular details for substrate recognition.
While the work is clearly of high interest, well executed, and generally well presented, a few major concerns remain.
On several occasions, statements do not seem to be clearly supported by the data presented or appear rather exaggerated to insist in the novelty of the study. For instance, in the abstract: "unusual regulatory mechanism", "distinct two-metal-ion catalytic mechanism", could the author explain what is "unusual" or "distinct" regarding theses mechanisms? The same is true regarding the evolutionary relevance of this "RNA only" RNAse P. While the author suggests this may bring important insights into the functioning of an ancestral protein-less ribozyme, it is unclear to what extent this is true given that the RNA has evolved along with its protein partners. The authors should either ton down this aspect, including modifying the title, or justify better the evolutionary implication of their work. For example, the statement p12 "Since RPR is the only naturally occurring trans-acting ribozyme, this finding is both intriguing and significant" what is their exact meaning for "naturally occurring"?
The rationale behind conducting structural studies in the presence of Mg or Ca is not sufficiently justified and statements such as "consistent with Ca2+ used in this study to prevent catalysis (Fig. 5b)" (p10) should be accompanied by relevant references or functional data. Moreover, the author should justify better why Ca2+ blocks catalysis and to what extent it is still believed to mimic accurately Mg ions in the active site. Importantly, considering the enzymatic assay presented in Fig 3, no product is visible, in the WT condition, before 10 min. Grid preparation typically falls within a fraction of this time. Can the authors explain why they did not report an APOE-pre-tRNA-Mg structure and had to use Ca2+, instead?
The Materials and Methods section should be more explicit about how the complexes between tRNA and RNase P were formed (e.g., molar ratios, incubation times and temperatures, buffer conditions). In addition, could the authors comment on the surprisingly higher proportion of particles in complex with mat-tRNA in the enzyme-product sample (at least 50% of particles, Supp. Fig. 5) compared with the enzyme–substrate sample (fewer than 30%, Supp. Fig. 4)? Although particle distributions are not an ideal metric for assessing complex formation, this discrepancy is surprising.
The model building seems generally of good quality but does not acknowledge clearly enough the contribution of previous structural studies on the same object in in guiding their modelling the metal ion present in the active site. In addition, could the authors comment on the fact that the RNA was refolded and purified in the presence of monovalent ions, known to be important for structure and stability of RNAs and that are missing from the structure. Could the author justify better their choice of modelling additional densities as Mg/Ca and never consider the possibility of monovalent ions, present in the buffers?
The authors set up a functional assay to test IDTM-TL/TLR interaction on activity of the enzyme and show that the mutations prevent t-RNA cleavage. They further interpret this result by failure of RNAse P to interact with its tRNA substrate by reporting the observation that mixing a threefold excess of tRNA with the enzyme did not yield stable complex as observed in cryo-EM. This method does not appear the most adequate to study interactions and this paper would clearly benefit from a systematic interactions' study of not only for TLm mutant but also toehold contacts. Unless, the authors can justify the observed interfaces by citing relevant previously published literature or raise major experimental difficulties.
The authors call upon coordination for the identification of active site ions in different conditions. While coordination is a safe way to identify different ion species present in different complexes, a more accurate comparison between coordination of Ca

and Mg is needed where possible; especially for those structures obtained in absence of any substrate and in presence of either the same or different divalent ion at different concentration. Figure 5 reports densities and coordination of MeA and MeB in the active site, this figure would benefit of a clearer legend pointing to the ionic conditions from which every sub figure was made (Ca 5mM/LB, Mg apoE etc etc). Fig 5a does not shows coordination distances, while Fig 5c does but the values do not correspond to any of the structure provided for review...
Minor
In the abstract, it is unclear what the authors are referring to with “cation traffic”.
P4:” We report here the high-resolution apoE structures of Geobacillus stearothermophilus (Gst) RNase P RNA and its complexes with pre-tRNA (apoES) and product mat-tRNA (apoEP).” Please modify high-resolution by measurable values.
P4: “Importantly, the cryo-EM volumes in our study are complete for the entire S-domain and sufficient for atomic modeling (Extended Data Fig. 1a and b).” "complete" should be changed.
P4: “With the three primary structures of apoE and its complexes with cognate pre-tRNA”: cognate is strictly used in case of codon anticodon interactions, in this case substrate is recognized via conserved three-dimensional structures as clearly stated in the manuscript.
Fig 2. Could the authors make this figure easier to read by making a more obvious connexon between panel j and the (1) … (6) notation and panels d. to i. .
P7 “These structures visualize how the RNase P could access and cleave partially structured regions between justaposed pre-tRNAs on polycistronic transcripts [PMID:17981836].” Should be corrected and the PMID code changed.
P13: “Studies of DNA polymerase28-30 and protein nucleases19 provided structural evidence of a third catalytic metal ion. Additionally, three-metal-ion mechanisms for catalysis have been proposed for DNA polymerase28-30 and Group I and II introns18,31,32.” It is unclear from this statement in the 3rd metal ion has been observed or hypothesized in DNA polymerase.
Extended data Fig 5 is missing PDB or relevant references in the caption or figure.
P26: “Samples included 2 uM”, could the author provide the volume or the quantity of matter instead of a concentration?

Reviewer #3

(Remarks to the Author)
I co-reviewed this manuscript with one of the reviewers who provided the listed reports. This is part of the Nature Communications initiative to facilitate training in peer review and to provide appropriate recognition for Early Career Researchers who co-review manuscripts.

Version 1:

Reviewer comments:

Reviewer #1

(Remarks to the Author)
The revised manuscript addresses all concerns raised by this reviewer, and, from this reviewer's perspective, suitable for publication.

Reviewer #2

(Remarks to the Author)
The revised version if largely satisfactory but a few minor points still require clarifications:

1/
The Materials and Methods section should be more explicit about how the complexes between tRNA and RNase P were formed (e.g., molar ratios, incubation times and temperatures, buffer conditions). In addition, could the authors comment on the surprisingly higher proportion of particles in complex with mat-tRNA in the enzyme-product sample (at least 50% of particles, Supp. Fig. 5) compared with the enzyme–substrate sample (fewer than 30%, Supp. Fig. 4)? Although particle distributions are not an ideal metric for assessing complex formation, this discrepancy is surprising.
Reply: We provided more experimental details in Methods (page 25, lines 557-561) for the formation of complexes (RNase P RNA to tRNA molar ratio (1:3), 30-minute incubation at 4 C; buffer condition: 25 mM Tris HCl buffer pH 7.5, 100 mM NaCl, 5 mM $CaCl_2$). Regarding the proportions of complexed particles in the apoES and apoEP data, we believe the reviewer may be looking at the three subclasses of apoES, each of which contains ~55-65 K particles. The consensus volume, however, contains ~180K particles, which is ~97% of the particles from the ab-initio reconstruction. Therefore, there is a significantly higher population of complexed particles in apoES data as compared to apoEP.

A minor point of disagreement on this. The 3 subclasses presented for ApoES (SuppFig 4) don’t show consistent density for the substrate: only class 2 displays evident signal for the substrate. Hence class 0 and class 1 can be considered as “unbound”. Unless mistaken, our initial comment on the proportion of E:S vs. E:P remains.

2/
The model building seems generally of good quality but does not acknowledge clearly enough the contribution of previous structural studies on the same object in guiding their modelling of the metal ion present in the active site. In addition, could the authors comment on the fact that the RNA was refolded and purified in the presence of monovalent ions, known to be

important for structure and stability of RNAs and that are missing from the structure. Could the author justify better their choice of modelling additional densities as Mg/Ca and never consider the possibility of monovalent ions, present in the buffers? Reply: Yes, we appreciate the reviewers' concerns about the metal ions, one of the notable results of our work. We did acknowledge and compare the metal ions in the catalytic site (MeA and MeB) in our RNase P structures with the ones in the bacterial A-type RNase P structure, the only RNase P structure with the catalytic metal ions modeled (Reiter, Osterman et al. 2010). Please see Extended Data Fig. 7a and 7b and the related description in the main text, page 12, lines 252-259. Although the metal ion A (MeA) was not modeled in the A-type RNase P structure(Reiter, Osterman et al. 2010) (PDB ID: 3Q1R), both catalytic metal ions (MeA and MeB) in our RNase P structures are consistent with those observed in other protein-only RNase structures (Extended Data Fig. 7a, 7c, and 7d).
To the best of our knowledge, there are no other reference structures to compare with for other types of metal ions of RNase P in terms of their position and coordination geometry. To support the scientific rigor of the identity of metal ions in the RNase P RNA structures (Fig. 4), we performed an analysis of the structures using the CheckMyMetal program (Zheng, Chordia et al. 2014), providing validations of ions (see Supplementary Table 3) by assessing coordination geometry, distance, occupancy, etc. Although we cannot completely rule out the possibility of Na+ in some ambiguous cases, we decided to model all as divalent ions, given the saturating concentrations of divalent ions in the buffer, the much higher affinity of divalent ions compared to monovalent ions, and the fact that ordered Na+ ions are rarely observed or reported in RNA structures. Moreover, all assigned metal ions in our structures have great cryo-EM volume occupancy and Q-score (Extended Data Fig. 2).

Please include more clearly the scientific justification for ion assignment as above in the Material and methods and/or main text.

Reviewer #3

(Remarks to the Author)
I co-reviewed this manuscript with one of the reviewers who provided the listed reports. This is part of the Nature Communications initiative to facilitate training in peer review and to provide appropriate recognition for Early Career Researchers who co-review manuscripts.

Open Access This Peer Review File is licensed under a Creative Commons Attribution 4.0 International License, which permits use, sharing, adaptation, distribution and reproduction in any medium or format, as long as you give appropriate credit to the original author(s) and the source, provide a link to the Creative Commons license, and indicate if changes were made.
In cases where reviewers are anonymous, credit should be given to 'Anonymous Referee' and the source.
The images or other third party material in this Peer Review File are included in the article's Creative Commons license, unless indicated otherwise in a credit line to the material. If material is not included in the article's Creative Commons license and your intended use is not permitted by statutory regulation or exceeds the permitted use, you will need to obtain permission directly from the copyright holder.
To view a copy of this license, visit https://creativecommons.org/licenses/by/4.0/

Reviewer #1 (Remarks to the Author):

While the crystal structure of the bacterial RNase P has been determined about two decades ago, the advent of cryo-EM has opened new opportunities to shed light on its details and to provide novel insight into its function. The authors of this manuscript have capitalized on this opportunity and solved the structure of a B-type bacterial RNase P RNA in several states of the catalytic cycle. While the RNase P holoenzyme is a ribonucleoprotein complex and the molecule studied in the manuscript lacks the protein component, the results of this study are still relevant and interesting, given that the RNA component alone is capable of catalysis under special conditions.

The structures presented in the manuscript are important and reveal novel details of the B-type bacterial RNase P RNA structure and function. Of special note is the observation of the conformational changes occurring as catalysis progresses: this is a very important contribution to the field that alone makes the manuscript worthy of publication.

However, this reviewer has several concerns (below) that must be addressed before this work is, in the view of this reviewer, suitable for publication. The major concern is the rather reckless (or, at the very least, poorly justified in the text) assignment of ion identities to all potentially suitable peaks in the maps. The authors should either strongly justify these assignments in each and every case, or scale down the related claims (see Auffinger et al. RNA 2021); the results are interesting even without the incorporation of potentially less-than-rigorous ion assignments.

Reply: We thank the reviewer for encouraging comments about our manuscript. We've toned down our statements and added references relevant to assigning/identifying metal ions in several places based on the reviewer's comments. Please see the following point-by-point responses. Given the buffer conditions for each structure, the only probable ions are Na^+ and Ca^{2+}/Mg^{2+}. We analyzed metal ions using the CheckMyMetal program (Zheng, Chordia et al. 2014) by assessing coordination geometry, contact, distance, B-factor, and occupancy of the metal ions in our structures (summarized in **Supplementary Table 3)**. Although we cannot completely rule out the possibility of Na^+ in some ambiguous cases, given the saturating concentrations of divalent ions in the buffer and that ordered Na^+ ions are rarely observed or reported in RNA structures, we modeled all solvent ion peaks as divalent ions. All assigned metal ions in our structures exhibit high cryo-EM volume occupancy and Q-scores (**Extended Data Fig. 2**).

- **Comments:**

Ln 27-28. Consider re-wording for clarity.

Reply: We have modified the statement in the abstract for clarity (**page 2, lines 27-28**).

"However, the structural basis for the ancient RNase P RNA component to act alone as a ribozyme, including catalytic metal-ion coordination, has not been determined."

Ln 48-49. The evolutionary relevance of the activity is not unknown.

Reply: We have modified the statement to clarify that we are referring to the significance of protein-free activity (**page 3, lines 45-48**).

"In bacteria and some archaea, the RNA component of RNase P (RPR) exhibits intrinsic catalytic activity in vitro even without its protein subunit, although the mechanism behind this protein-free ribozyme activity and its evolutionary significance are unclear."

Ln 53. Weak activity has been reported for RNA alone: Kikovska *et al.* PNAS 2007. Roles of individual proteins are described in Perederina et al. NAR 2018. It would be appropriate to cite both.

Reply: The references have been cited (**page 3, line 53**).

"In contrast, the eukaryotic RPRs require multiple protein subunits for catalytic activity both in vitro and in vivo (Kikovska, Svard et al. 2007, Perederina, Berezin et al. 2018)."

Ln 88. While technically this is a correct statement, it appears to be somewhat misleading. It must be mentioned that structures of the S-domain in isolation, as well as the S-domain as a part of RNP, have been reported, and the corresponding references must be provided.

Reply: We added a sentence and cited relevant references (**page 5, lines 89-92**).

"In the context of apoE, this is particularly significant, as no complete structure of any RNase P RNA alone has ever been reported. The structure of the S-domain was determined in isolation but was mostly unresolved in the full-length apoenzyme structure (Krasilnikov, Yang et al. 2003, Kazantsev, Krivenko et al. 2005)."

Ln 148-149. The structural basis of the S-domain's role has been discussed in Krasilnikov *et al.* Science 2004.

Rely: We toned down and rephrased the sentence (**page 8, lines 158-159**).

"Although the function of the S-domain has been discussed (Krasilnikov, Xiao et al. 2004), the structural basis for its regulatory role in RNase P activity has not been demonstrated."

Ln 151. Ext. Data Fig. 5: References must be provided.

Reply: We cited references regarding the bacterial, archaeal, and human RNase P IDTM structural comparison (Reiter, Osterman et al. 2010, Wu, Niu et al. 2018, Wan, Wang et al. 2019). (**page 8, lines 161**)

Ln 152-153. The role of the TL/TLR, including its contribution to stabilizing the IDTM region, was discussed in Krasilnikov et al. Nature 2003 and Science 2004; this must be acknowledged.

Reply: We cited the two references to support the TL/TLR role in stabilizing IDTM (Krasilnikov, Yang et al. 2003, Krasilnikov, Xiao et al. 2004) and rephrased the sentence (**page 8, lines 162-165**).

*"Despite its conservation and role in stabilization of IDTM in S domain (Krasilnikov, Yang et al. 2003, Krasilnikov, Xiao et al. 2004), the TL/TLR does not make direct contact with the substrate (**Fig. 2a**), and the structural interplay between the IDTM and TL/TLR for regulating substrate recognition is not clear."*

Ln 168. A- and B-type RNases Ps differ in their structural arrangements. The broader implications of the suggested "allosteric switch" role of TL/TLR should be discussed, taking into account these differences. Alternatively, it should be clearly stated that the findings discussed in the manuscript are limited to a specific type of RNase P, without unwarranted generalizations.

Reply: We agree with the reviewer that such allostery may be specific to **"B-type"** RNase P. We modified the sentence and cited the reference that supports the TL/TLR role in the stabilization of IDTM (**pages 9, lines 180-183**).

*"In summary, although the relative distance between TL/TLR and IDTM is greater than 40 Å (**Fig. 3a**), the TL/TLR interaction is intimately coupled with the proper folding of the IDTM. This coupling could be considered an allosteric switch for **B-type RNase P** substrate recognition (Krasilnikov, Yang et al. 2003, Krasilnikov, Xiao et al. 2004)."*

Ln 181 and so on. **A major concern**. Assigning Mg^{2+}/Ca^{2+} to all 10+ sigma picks (and there are dozens of those) without proper justification is reckless (see Auffinger et al. RNA 2021). Proper and thorough justification should be given to the differentiation between Mg^{2+}/Ca^{2+} and water/other metal ions (Na^{+}, specifically); when it's not possible, this should be clearly stated. As is, these assignments are putative, at best.

Reply: Please see our response for **lines 20-28** in this rebuttal and **Supplementary Table 3**. We provided the table to validate the presence and type of metal ions in our structures. We have also added the following sentence on **page 9** from **lines 195-199** to acknowledge uncertainty**.**

*“Some ions could not be identified unambiguously. However, given the much greater affinity of divalent ions, and that no ions showed any strong indication of being monovalent (**Supplementary Table 3**), all ions in the structures were modeled as Mg^{2+} or Ca^{2+}, based on buffer conditions (**Methods**).”*

Ln 271-272. This claim requires a rigorous proof of the ion’s identity, which is not provided.

Reply: Our study provides conclusive structural evidence of two metal ions (MeA and MeB) in the active site of our apoES structure (**Fig. 3d**). Based on all possible metrics and validation, these ions are unquestionably divalent, namely Mg^{2+} or $Ca^{2+,}$ depending on the buffer conditions (**Methods**). As for the third metal ion (MeC) proposed for the holoenzyme, this is based solely on the structure of A-type RNase P holoenzyme, which reported a metal ion in a position distinct from either MeA or MeB (**Extended Data Fig. 7b**). We do not make any claims to the identity of that ion, but only mention it here for discussion.

Ln 668 and below. It would be appropriate to acknowledge actual publications, not just PDB IDs.

Reply: We cited the references corresponding to the structures with PDB IDs: **2A64** (Kazantsev, Krivenko et al. 2005) and **1NBS** (Krasilnikov, Yang et al. 2003), **page 27**, **lines 623-624**.

Table 1. Geometry parameters, including geometry outliers, must be included in the table.

Reply: We now include geometry parameters and the number of geometry outliers in **Supplementary Tables 1 and 2**.

- **Provided maps and PDB files:**

1. Refinement appears to be sloppy in many instances and has to be redone; this would be evident if geometry parameters of the models were provided, but even the provided excessively high clash scores are telling.

Reply: We appreciate the reviewer’s efforts to report the most accurate structures possible. We’ve re-refined all structures with higher geometric constraints. The clash scores of all structures are now lower than 10 and are provided along with other statistics in **Supplementary Tables 1 and 2**.

2. There is evidence (such as B-factors set to 20.00) of post-refinement (“by hand”) modifications of the models (mutations of residues?).
Reply: We thank the reviewer for catching those. They have now been corrected in the re-refined structures.

3. In the supplementary information, plots showing per-residue correlation coefficients for all models must be presented. This is especially important given the low local resolution in the peripheral parts of the molecules.

Reply: We have provided the **per-residue correlation coefficient plots** for all structures in **Supplementary Fig. 7**.

Reviewer #2 (Remarks to the Author):

This paper focuses on an important and timely biological topic: the mechanism of catalysis and substrate recognition by bacterial RNase P. This conserved ribonucleoprotein uses an RNA core to catalyze tRNA 5' end processing. The authors present several cryo-EM structures of the RNA core of RNase P in its “apo” form (*i.e.*, without its protein components), WT/mutant, and in complex with substrate, product, or various concentrations of Mg^{2+}/Ca^{2+}. A limited amount of functional data is also provided. The novel insights arising from these structures are suggested to include the first visualization of the S-domain, identification of several divalent metal ions that stabilize the structure, and molecular details for substrate recognition.

While the work is clearly of high interest, well executed, and generally well presented, **a few major concerns remain**. On several occasions, statements do not seem to be clearly supported by the data presented or appear rather exaggerated to insist on the novelty of the study.

Reply: We thank the reviewer for constructive comments and praise of our work. Please see our point-by-point responses to the statements that require further support or clarification.

For instance, in the abstract: **"unusual regulatory mechanism", "distinct two-metal-ion catalytic mechanism",** could the author explain what is "unusual" or "distinct" regarding these mechanisms?

Reply: Regarding "unusual regulatory mechanism," we see the reviewer's point of unclear and unnecessary language. We have removed this phrase from the abstract. Regarding the two-metal-ion catalytic mechanism, the word "distinct" was meant to convey differences from what has been previously reported for the A-type RNase P holoenzyme structure (Reiter, Osterman et al. 2010). See the **Extended Data Fig. 7a and 7b** that compare the metal ion position in the RNase P catalytic site, as well as the related description in the main text, **page 12, lines 252-259**. However, as the abstract itself does not draw any direct comparisons, we agree that the word "distinct" is ambiguous here and has been removed (**page 2, line 33**).

The same is true regarding the evolutionary relevance of this "RNA-only" RNase P. While the author suggests this may bring important insights into the functioning of an ancestral protein-less ribozyme, it is unclear to what extent this is true, given that the RNA has evolved along with its protein partners. The authors should either tone down this aspect, including modifying the title, or better justify the evolutionary implications of their work. For example, the statement p12 "Since RPR is the only naturally occurring trans-acting ribozyme, this finding is both intriguing and significant." What is their exact meaning for "naturally occurring"?

Reply: We agree with the reviewer that, although an ancient protein-free RNase P is plausible evolutionarily, there is no direct evidence to support this notion, and it should be left as speculation. We have thus removed **"in a primordial RNA world"** from the title, and amended it to "***Structural basis for protein-free catalysis by ribonuclease P ribozyme***".

The sentence on **page 3, lines 45-48,** was also modified:
"In bacteria and some archaea, the RNA component of RNase P (RPR) exhibits intrinsic catalytic activity in vitro even without its protein subunit, although the mechanism behind this protein-free ribozyme activity and its evolutionary significance are unclear."

Regarding "naturally occurring", we were referring to RNase P holoenzymes as they are found in nature, not RPR alone. We have revised the statement (**page 13, lines 290-292**).

"Since RNase P is the only trans-acting ribozyme identified in nature, this finding is both intriguing and significant. It suggests a possible ancestral, protein-independent two-metal-ion mechanism for substrate-assisted catalysis."

The rationale behind conducting structural studies in the presence of Mg2+ or Ca2+ is not sufficiently justified and statements such as "consistent with Ca^{2+} used in this study to prevent catalysis (**Fig. 5b**)" (p10) should be accompanied by relevant references or functional data. Moreover, the author should justify better why Ca^{2+} blocks catalysis and to what extent it is still believed to mimic accurately Mg^{2+} ions in the active site. Importantly, considering the enzymatic assay presented in Fig. 3, no product is visible, in the WT condition, before 10 min. Grid preparation typically falls within a fraction of this time. Can the authors explain why they did not report an APOE-pre-tRNA-Mg structure and had to use Ca^{2+}, instead?

Reply: The reviewer makes an excellent point based on our activity assay in **Fig. 3**. However, the apoE and pre-tRNA concentrations required for cryo-EM grid preparation are "30 times" higher than those used for the activity assay. The elevated apoE and pre-tRNA substrate concentrations will accelerate the pre-tRNA cleavage. We conducted a time-course experiment using gel electrophoresis with the cryo-EM concentrations of both apoE and pre-tRNA substrate at 5 mM Mg^{2+} (**see gel image below**). It showed that the pre-tRNA substrate is mostly cleaved within 1 minute.

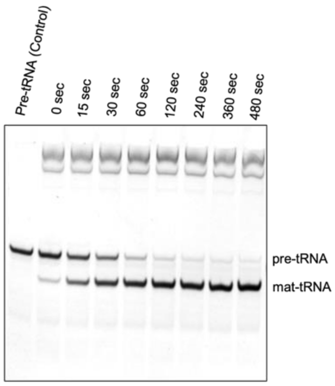

We have made our best effort to determine the apoE-pre-tRNA complex structure in a 5 mM Mg^{2+} buffer condition, but the particles from cryo-EM micrographs turned out to be highly heterogeneous, resulting in very low particle numbers for 3D reconstruction of the apoE-pre-tRNA complex. It is well established that RNA interactions with Ca^{2+} are very similar, but not identical, to those with Mg^{2+} in stabilization of RNA structure (Koculi, Hyeon et al. 2007, Hsieh, Koutmou et al. 2010). Both divalent cations preferentially bind to phosphate oxygens and can participate in both outer- and inner-sphere coordination to neutralize negative charge and promote stable and more compact folds. With respect to catalysis, however, Mg^{2+}-dependent RNase P activity is inhibited by Ca^{2+} (Frank and Pace 1997, Brannvall and Kirsebom 2001), due to their differences in size, charge density, bond lengths, and coordination geometry that are critical for precise active-site configuration.

The **Materials and Methods** section should be more explicit about how the complexes between tRNA and RNase P were formed (e.g., molar ratios, incubation times and temperatures, buffer conditions). In addition, could the authors comment on the surprisingly higher proportion of particles in complex with mat-tRNA in the enzyme-product sample (at least 50% of particles, **Supp. Fig. 5**) compared with the enzyme–substrate sample (fewer than 30%, **Supp. Fig. 4**)? Although particle distributions are not an ideal metric for assessing complex formation, this discrepancy is surprising.

Reply: We provided more experimental details in **Methods** (**page 25, lines 557-561**) for the formation of complexes (RNase P RNA to tRNA molar ratio (1:3), 30-minute incubation at 4°C; buffer condition: 25 mM Tris HCl buffer pH 7.5, 100 mM NaCl, 5 mM $CaCl_2$). Regarding the proportions of complexed particles in the apoES and apoEP data, we believe the reviewer may be looking at the three subclasses of apoES, each of which contains ~55-65 K particles. The consensus volume, however, contains ~180K particles, which is ~97% of the particles from the *ab-initio* reconstruction. Therefore, there is a significantly higher population of complexed particles in apoES data as compared to apoEP.

The model building seems generally of good quality but does not acknowledge clearly enough the contribution of previous structural studies on the same object in guiding their modelling of the metal ion present in the active site. In addition, could the authors comment on the fact that the RNA was refolded and purified in the presence of monovalent ions, known to be important for structure and stability of RNAs and that are missing from the structure. Could the author justify better their choice of modelling additional densities as Mg/Ca and never consider the possibility of monovalent ions, present in the buffers?

Reply: Yes, we appreciate the reviewers' concerns about the metal ions, one of the notable results of our work. We did acknowledge and compare the metal ions in the catalytic site (MeA and MeB) in our RNase P structures with the ones in the bacterial A-type RNase P structure, the only RNase P structure with the catalytic metal ions modeled (Reiter, Osterman et al. 2010). Please see **Extended Data Fig. 7a and 7b** and the related description in the **main text, page 12, lines 252-259.** Although the metal ion A (MeA) was not modeled in the A-type RNase P structure(Reiter, Osterman et al. 2010) (PDB ID: 3Q1R), both catalytic metal ions (MeA and MeB) in our RNase P structures are consistent with those observed in other protein-only RNase structures (**Extended Data Fig. 7a, 7c, and 7d**).

To the best of our knowledge, there are no other reference structures to compare with for other types of metal ions of RNase P in terms of their position and coordination geometry. To support the scientific rigor of the identity of metal ions in the RNase P RNA structures (**Fig. 4**), we performed an analysis of the structures using the CheckMyMetal program

(Zheng, Chordia et al. 2014), providing validations of ions (see **Supplementary Table 3**) by assessing coordination geometry, distance, occupancy, *etc*. Although we cannot completely rule out the possibility of Na^+ in some ambiguous cases, we decided to model all as divalent ions, given the saturating concentrations of divalent ions in the buffer, the much higher affinity of divalent ions compared to monovalent ions, and the fact that ordered Na^+ ions are rarely observed or reported in RNA structures. Moreover, all assigned metal ions in our structures have great cryo-EM volume occupancy and Q-score (**Extended Data Fig. 2**).

Regarding how we prepared the RNA sample for cryo-EM studies, we did not denature the RNA at any point during the purification (see our **Methods, page 22, lines 467-486; page 23, lines 503-521**). We have removed the incorrect word, "refolded", from **line 604**, which was the cause of confusion. After *in vitro* transcription (IVT) for 3 hours, the RNase P RNA, pre-tRNA substrate, and mat-tRNA product were purified individually using size exclusion chromatography with the buffer containing 25 mM Tris, pH 7.5, 100 mM NaCl, and 1 mM $CaCl_2$. RNase P RNA preparation followed a protocol involving a 200 mM-NaCl, 12-hour incubation step after IVT to prevent misfolding and denatured species (see **Methods, page 22, lines 467-486**). We prepared the apoES and apoEP complexes in the same buffer solution, but with a higher concentration of $CaCl_2$ of 5 mM for stabilization of apoES and apoEP complexes when we mixed the enzyme with substrate or with product.

The authors set up a functional assay to test IDTM-TL/TLR interaction on activity of the enzyme and show that the mutations prevent tRNA cleavage. They further interpret this result by failure of RNase P to interact with its tRNA substrate by reporting the observation that mixing a threefold excess of tRNA with the enzyme did not yield a stable complex as observed in cryo-EM. This method does not appear the most adequate to study interactions and this paper would clearly benefit from a systematic interactions' study of not only for TLm mutant but also toehold contacts. Unless, the authors can justify the observed interfaces by citing relevant previously published literature or raise major experimental difficulties.

Reply: We added one experimental result using mass photometry to address the reviewers' concern about how the TL mutation affects substrate binding and RNase P activity. Mass photometry was used to determine the ES population under different E/S ratios for both WT and TLm versions of apoE. These ratios were then used to derive the enzyme—substrate dissociation constant (K_D) for each. Per the results, the TL mutation weakens the RNase P RNA binding affinity to pre-tRNA by ~17-fold (see **Fig below**), explaining why the structure of apoES-TLm could not be determined at the threefold excess of pre-tRNA to the enzyme. This result has been included as **Fig. 3f** in the main text, and the corresponding narrative can be found on **pages 8-9, lines 178-180**. The experimental details can be found in the **Methods** (**pages 28-29, lines 663-693**). Additional data is shown in **Fig. 8** in **Supplementary Figures**.

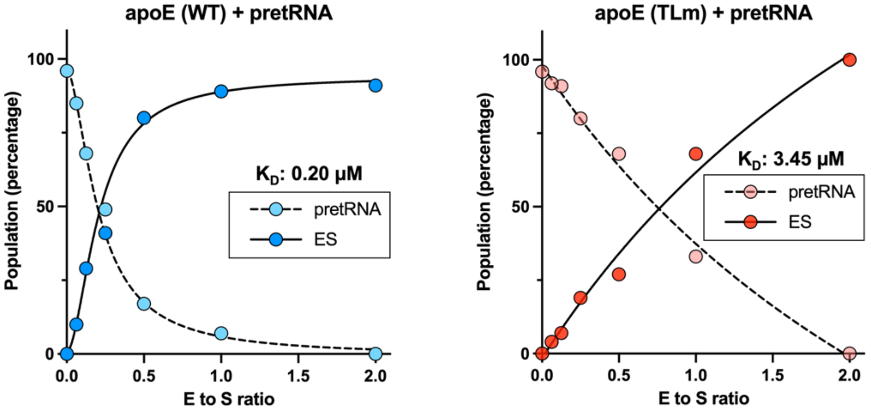

Although we did not conduct a systematic investigation for those six toeholds by mutations, their importance for RNase P can be justified because the nucleotides in the six toeholds are "phylogenetically sequence-conserved" (**Fig. 2a and 2b**), suggesting that they are all functionally important. In addition, we have included several references to support the importance of the toeholds for RNase P activity, all of which have been cited in the main text (**pages 6-7, lines 134-135**):

- For the IDTM-tRNA elbow region interaction (Zhang 2024).

- For the demarcator A256, equivalent to the nucleotide A248 of *E. coli* RNase P (Mao, Srivastava et al. 2023).
- For the pre-tRNA 3' CCA trailer-RNase P interaction (Oh and Pace 1994, Pascual and Vioque 1999, Wegscheid and Hartmann 2006).

The TL/TLR interaction is of particular interest because it is sequence-conserved but not in the substrate-binding interface. Its role has been discussed for the stabilization of IDTM for B-type RNase P (Krasilnikov, Yang et al. 2003, Krasilnikov, Xiao et al. 2004), which are now also referenced in the main text (**page 8, line 163**).

The authors call upon coordination for the identification of active site ions in different conditions. While coordination is a safe way to identify different ion species present in different complexes, a more accurate comparison between the coordination of Ca and Mg is needed, where possible, especially for those structures obtained in the absence of any substrate and in the presence of either the same or different divalent ions at different concentrations.

Reply: We agree with reviewer that we need to do a more accurate comparison for the active site metal ions (MeA and MeB) in the structures with and without a substrate under Ca^{2+} vs Mg^{2+} conditions. The MeB is only observed in apoES structure, and we cannot determine the apoES structure under Mg^{2+} conditions (see explanation in our previous response of **lines 202-224** in this rebuttal). As a result, the coordination geometry of MeB cannot be compared under the two different types of divalent ion conditions. MeA can be observed throughout the complete enzymatic cycles. We did not see significant differences in MeA in terms of its positioning and coordination geometry under various buffer conditions (see the **following Figure**).

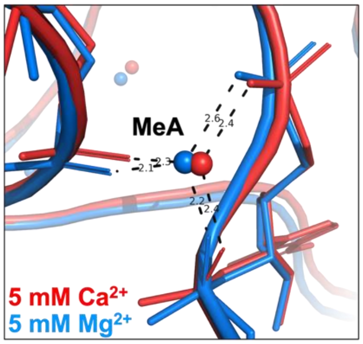

We included the detailed catalytic metal comparison for **MeA** between Ca^{2+} and Mg^{2+} in the apoE structures in **Extended Data Fig 2c**. The active site ions and their validation are included in **Supplementary Table 3**.

Figure 5 reports densities and coordination of MeA and MeB in the active site. This figure would benefit from a clearer legend pointing to the ionic conditions from which every sub-figure was made (Ca 5mM/LB, Mg apoE, *etc*).

Reply: We thank the reviewer for this helpful suggestion. All structures for making **Fig 5** were determined under 5 mM Ca^{2+}. We added a description in the main text (**Page 11; lines 230**) and in the **Fig. 5** legend to note these conditions (**main text, page 20, line 447**)

Fig. 5a does not show coordination distances, while **Fig. 5c** does, but the values do not correspond to any of the structures provided for review.

Reply: For clarity, we did not show the coordination distances in **Fig. 5a**, since this figure panel focuses on presenting peaks in the electron density corresponding to waters and metal ions. Instead, the distances are provided in **Fig. 5c**. The model used to prepare this figure is the apoES-pre-tRNA complex structure (**PDB ID: 9OWJ**).

Minor

In the abstract, it is unclear what the authors are referring to with “cation traffic”.

Reply: The cation traffic refers to “b-class metal ions” in **Fig. 4b** and one of the catalytic metal ions, “MeB”. These ions are not observed in other enzymatic states (apoE and apoEP). The “b-class” metal ions are trafficking, where they may be recruited from the surrounding environment or supplied by the substrate.

P4: “We report here the high-resolution apoE structures of *Geobacillus stearothermophilus* (Gst) RNase P RNA and its complexes with pre-tRNA (apoES) and product mat-tRNA (apoEP).” Please modify the high-resolution by measurable values.

Reply: We modified and provided a resolution range of our structures (**see main text page 4, lines 68-70**).

“We report here the apoE structures of Geobacillus stearothermophilus (Gst) RNase P RNA and its complexes with pre-tRNA (apoES) and product mat-tRNA (apoEP) ***at resolutions of 2.8-3.5 Å****.”*

P4: “Importantly, the cryo-EM volumes in our study are complete for the entire S-domain and sufficient for atomic modeling (**Extended Data Fig. 1a and b**).” "complete" should be changed.

Reply: We removed “complete” and rephrased (**see main text, pages 4-5, lines 88-89**).

*“Importantly, the entire S-domain could be modeled for all cryo-EM volumes in this study (****Extended Data Fig. 1a and b****).”*

P4: “With the three primary structures of apoE and its complexes with cognate pre-tRNA”: cognate is strictly used in case of codon anticodon interactions; in this case, substrate is recognized *via* conserved three-dimensional structures as clearly stated in the manuscript.

Reply: We removed “cognate” (**see main text, page 4, line 80)** and other places in the manuscript.

“With the three primary structures of apoE and its complexes with pre-tRNA substrate (apoES)…”

Fig 2. Could the authors make this figure easier to read by making a more obvious connection between panel j and the (1) … (6) notation and panels d. to i..

Reply: We thank the reviewer for this suggestion. We’ve remade **Fig. 2** by combining panel j with panels d. to i.

P7: “These structures visualize how the RNase P could access and cleave partially structured regions between juxtaposed pre-tRNAs on polycistronic transcripts [PMID:17981836].” Should be corrected and the PMID code changed.

Reply: We’ve corrected this. (**see main text, page 7, line 154**)

P13: “Studies of DNA polymerase 28-30 and protein nucleases 19 provided structural evidence of a third catalytic metal ion. Additionally, three-metal-ion mechanisms for catalysis have been proposed for DNA polymerase28-30 and Group I and II introns 18,31,32.” It is unclear from this statement in the 3rd metal ion has been observed or hypothesized in DNA polymerase.

Reply: The presence of three metal ions in DNA polymerase is observed and supported by direct structural evidence (Nakamura, Zhao et al. 2012, Chang, Lee Luo et al. 2022). What has been proposed is the mechanism of catalysis.

Extended data Fig. 5 is missing PDB or relevant references in the caption or figure.

Reply: We cited the references corresponding to the structures with PDB IDs: **2A64** (Kazantsev, Krivenko et al. 2005) and **1NBS** (Krasilnikov, Yang et al. 2003) in both the main text and legend.

P26: “Samples included 2 uM”, could the author provide the volume or the quantity of matter instead of a concentration?

Reply: Yes, we provided more experimental details to address the concern regarding how we prepare the sample for the experiments. We used a 2 μM, 15 μLiter injection volume for LC-mass spectrometry. Please see our revised **Methods (page 24, lines 545-546).**

References:

Brannvall, M. and L. A. Kirsebom (2001). "Metal ion cooperativity in ribozyme cleavage of RNA." Proc Natl Acad Sci U S A **98**(23): 12943-12947.

Chang, C., C. Lee Luo and Y. Gao (2022). "In crystallo observation of three metal ion promoted DNA polymerase misincorporation." Nat Commun **13**(1): 2346.

Frank, D. N. and N. R. Pace (1997). "In vitro selection for altered divalent metal specificity in the RNase P RNA." Proc Natl Acad Sci U S A **94**(26): 14355-14360.

Hsieh, J., K. S. Koutmou, D. Rueda, M. Koutmos, N. G. Walter and C. A. Fierke (2010). "A divalent cation stabilizes the active conformation of the B. subtilis RNase P x pre-tRNA complex: a role for an inner-sphere metal ion in RNase P." J Mol Biol **400**(1): 38-51.

Kazantsev, A. V., A. A. Krivenko, D. J. Harrington, S. R. Holbrook, P. D. Adams and N. R. Pace (2005). "Crystal structure of a bacterial ribonuclease P RNA." Proc Natl Acad Sci U S A **102**(38): 13392-13397.

Kikovska, E., S. G. Svard and L. A. Kirsebom (2007). "Eukaryotic RNase P RNA mediates cleavage in the absence of protein." Proc Natl Acad Sci U S A **104**(7): 2062-2067.

Koculi, E., C. Hyeon, D. Thirumalai and S. A. Woodson (2007). "Charge density of divalent metal cations determines RNA stability." J Am Chem Soc **129**(9): 2676-2682.

Krasilnikov, A. S., Y. Xiao, T. Pan and A. Mondragon (2004). "Basis for structural diversity in homologous RNAs." Science **306**(5693): 104-107.

Krasilnikov, A. S., X. Yang, T. Pan and A. Mondragon (2003). "Crystal structure of the specificity domain of ribonuclease P." Nature **421**(6924): 760-764.

Mao, G., A. S. Srivastava, S. Wu, D. Kosek and L. A. Kirsebom (2023). "Importance of residue 248 in Escherichia coli RNase P RNA mediated cleavage." Sci Rep **13**(1): 14140.

Nakamura, T., Y. Zhao, Y. Yamagata, Y. J. Hua and W. Yang (2012). "Watching DNA polymerase eta make a phosphodiester bond." Nature **487**(7406): 196-201.

Oh, B. K. and N. R. Pace (1994). "Interaction of the 3'-end of tRNA with ribonuclease P RNA." Nucleic Acids Res **22**(20): 4087-4094.

Pascual, A. and A. Vioque (1999). "Substrate binding and catalysis by ribonuclease P from cyanobacteria and Escherichia coli are affected differently by the 3' terminal CCA in tRNA precursors." Proc Natl Acad Sci U S A **96**(12): 6672-6677.

Perederina, A., I. Berezin and A. S. Krasilnikov (2018). "In vitro reconstitution and analysis of eukaryotic RNase P RNPs." Nucleic Acids Res **46**(13): 6857-6868.

Reiter, N. J., A. Osterman, A. Torres-Larios, K. K. Swinger, T. Pan and A. Mondragon (2010). "Structure of a bacterial ribonuclease P holoenzyme in complex with tRNA." Nature **468**(7325): 784-789.

Wan, F., Q. Wang, J. Tan, M. Tan, J. Chen, S. Shi, P. Lan, J. Wu and M. Lei (2019). "Cryo-electron microscopy structure of an archaeal ribonuclease P holoenzyme." Nat Commun **10**(1): 2617.

Wegscheid, B. and R. K. Hartmann (2006). "The precursor tRNA 3'-CCA interaction with Escherichia coli RNase P RNA is essential for catalysis by RNase P in vivo." RNA **12**(12): 2135-2148.

Wu, J., S. Niu, M. Tan, C. Huang, M. Li, Y. Song, Q. Wang, J. Chen, S. Shi, P. Lan and M. Lei (2018). "Cryo-EM Structure of the Human Ribonuclease P Holoenzyme." Cell **175**(5): 1393-1404 e1311.

Zhang, J. (2024). "Recognition of the tRNA structure: Everything everywhere but not all at once." Cell Chem Biol **31**(1): 36-52.

Zheng, H., M. D. Chordia, D. R. Cooper, M. Chruszcz, P. Muller, G. M. Sheldrick and W. Minor (2014). "Validation of metal-binding sites in macromolecular structures with the CheckMyMetal web server." Nat Protoc **9**(1): 156-170.

A minor point of disagreement on this. The 3 subclasses presented for ApoES (SuppFig 4) don't show consistent density for the substrate: only class 2 displays evident signal for the substrate. Hence class 0 and class 1 can be considered as "unbound". Unless mistaken, our initial comment on the proportion of E:S vs. E:P remains.

Response:

The reviewer is correct that the final particle stack corresponding to the apoES structure volume is ~30% of the total particles (~180K before splitting). However, the other two subclasses did show weak, diffuse density for pre-tRNA, but not in the "active" position. As the electron density of pre-tRNA in these subclasses was too poor to model, we did not include this in the final list of structures reported. Nevertheless, the percentage of "pre-tRNA-containing" particles is therefore much higher. Upon retracing our steps for this dataset, we did not, in fact, apply a focus mask around the pre-tRNA region as we did in other data sets. We repeated 3D classification using the proper mask, and the other 2 subclasses separated more cleanly. We now have the original "active" complex subclass 1 (~31%), subclass 2 containing no pre-tRNA (~44%), and subclass 3 containing pre-tRNA in the "non-active" position (~25%). As such, the total number of pre-tRNA-containing particles is **actually 56%.** We have updated the manuscript and **Supplementary Fig. 12** accordingly.

As for the apoEP complex, the incorrect workflow was shown in **Supplementary Fig. 13**, and we wouldn't have realized this error had it not been for the reviewer's comment. The apoEP data went through many more 2D/3D classification iterations than other datasets. The initial stack of total "clean" particles before sorting in the 3D was ~300K, and the number of particles in the final apoEP volume was ~59K. Therefore, the percentage of total particles for **apoEP is ~20%** as compared to ~**56% for apoES**. We have updated the manuscript and **Supplementary Figs. 12 and 13** accordingly. We thank the reviewer for pointing this out.

Please include more clearly the scientific justification for ion assignment as above in the Material and methods and/or main text.

Our response:

We include the scientific justification for ion assignment in the **Methods section**. Please see the main text on **page 31, lines 752-766**.
